# Uncertainty-aware bilevel energy storage planning for active distribution networks: A fixed-topology DistFlow-based MISOCP framework with voltage-security assessment

**DOI:** 10.1371/journal.pone.0353192

**Published:** 2026-10-09

**Authors:** Zhenhua Miao, Ziyan Li, Yongjie Wang

**Affiliations:** 1 School of Energy and Electrical Engineering, Qinghai University, Xining, Qinghai, China; 2 Qinghai Key Lab of Efficient Utilization of Clean Energy, Qinghai University, Xining, Qinghai, China; Beijing University of Technology, CHINA

## Abstract

Historical renewable-load variability, voltage-security risk, and investment-operation coupling can materially change the assessed value of distribution-level energy storage. This study develops an uncertainty-aware bilevel energy storage system (ESS) planning framework in which the upper level selects a discrete siting-sizing-duration plan and the lower level solves a fixed-topology, multi-scenario mixed-integer second-order cone program (MISOCP) in which the network physics are represented by a DistFlow-SOCP formulation. An ESS-aware topology robustness check is further introduced to examine whether ESS-induced topology changes affect the planning conclusions obtained under the common-topology comparison. Complete daily load, photovoltaic, and wind observations from Denmark in 2016 and 2017 are represented as joint 72-dimensional trajectories. Input-risk-stratified historical-day reduction is examined for K = 3, 5, 10, and 20, with K = 10 used for the principal 33-bus study and K = 20 used as a higher-fidelity scenario-set check. Exhaustive evaluation of 211 alternatives identifies a 1.00 MW/ 2.00 MWh ESS at bus 24. Under K10 and the baseline fixed topology, the proposed design reduces daily TOTEX from 73,151.02 to 51,826.24 CNY/day (29.2%) relative to No ESS, raises the minimum voltage from 0.94138 to 0.95405 p.u., and reduces cumulative undervoltage exposure from 0.013528 to 0.001740 p.u.·h. Cross-year and seven-day chronological tests retain the economic and security pattern. On the released 240-bus feeder, the reference design improves voltage exposure, loading, and losses at a small incremental economic cost. These results show that historical tail conditions, common-topology attribution, and physical security metrics are important for auditable ESS planning.

## 1. Introduction

High penetrations of photovoltaic (PV) generation, wind power, flexible demand, and converter-interfaced resources are changing the operating conditions of distribution feeders. Variability in demand and renewable availability can create sustained low-voltage states, reverse power flow, and costly corrective dispatch. Energy storage systems (ESSs) can shift energy, buffer renewable fluctuations, and support voltage, but their value depends on how uncertainty, network physics, and investment cost are represented [1–6].

Prior work has developed stochastic, robust, and bilevel formulations for storage planning and active distribution network operation [7–15]. Network-constrained methods based on branch-flow or second-order-cone models improve physical fidelity while retaining computational tractability [16–18]. However, three issues remain important for a transparent planning comparison. First, generated or average-day inputs can suppress the historical tail conditions that carry the voltage-security signal. Second, allowing topology to vary among storage strategies creates a moving baseline. Third, monetary penalties alone do not establish physical security improvement.

This study addresses these issues through a historical-data-driven, fixed-topology bilevel framework. The upper level evaluates a complete discrete ESS siting-sizing-duration domain, whereas the lower level solves a fixed-topology multi-scenario MISOCP with DistFlow-SOCP network physics on one common radial topology. Daily-equivalent total expenditure (TOTEX) combines operating and annualized investment costs. Voltage performance is reported independently through the minimum voltage, undervoltage hours (UVH), maximum depth, cumulative undervoltage exposure (UVE), and mean voltage margin.

The main contributions are: (i) joint 72-dimensional historical load-PV-wind scenarios with input-risk stratification and exact worst-day-retention audits and K = 3, 5, 10, and 20 fidelity checks; (ii) exhaustive evaluation of the 211-alternative 33-bus design domain, with PSO, GA, and DE used only as search benchmarks; (iii) deterministic, cross-year, and seven-day chronological robustness checks under a common topology; and (iv) engineering-scale case study on a released 240-bus feeder, including location comparisons and a separate severe-stress experiment.

The remainder of the paper is organized as follows. **The Materials and methods section** presents the systems, historical scenarios, fixed-topology lower-level model, and discrete upper-level design domain. **The Results section** reports the scenario, ablation, planning, robustness, 240-bus, and sensitivity results. **The Discussion and Conclusions sections** discuss the implications, limitations, and conclusions.

Table 1 positions the study relative to representative work on uncertainty-aware ADN planning, ESS siting-sizing, and voltage-security assessment.

**Table 1:** Comparison of representative studies related to uncertainty-aware ADN planning and ESS planning.

Study	Uncertainty treatment	Objective and ESS/network formulation	Validation	Key limitation/ distinction
Lu et al. (2020)	Data-driven distributionally robust model calibrated from measured data	Minimization of ESS planning and operating costs with coordinated ESS operation in a high-PV distribution network	High-PV distribution-system case study	Strong data-driven robustness, but does not isolate ESS effects under a common fixed topology or provide cross-year voltage-risk validation
Wang et al. (2021)	Correlated DER uncertainty and tail dependence via two-stage uncertainty quantification	Voltage-unbalance mitigation through fixed/mobile storage allocation in a three-phase active distribution network	123-bus three-phase ADN	Focuses on voltage-unbalance mitigation rather than integrated TOTEX-based siting/sizing and physical tail-risk assessment
Li et al. (2022)	Uncertain DG output represented in bilevel planning	Joint DG/ESS investment and operating-economy optimization through bilevel siting/sizing with lower-level ESS operation and binary PSO	PG&E 69-bus distribution system	Uses bilevel joint planning, but ESS benefits are not attributed under one common fixed topology
Yi et al. (2022)	Stochastic PV generation and load	Distribution-grid expansion/reinforcement and ESS allocation for dispatchability using relaxed OPF and Benders decomposition	Real 55-node Swiss ADN	Emphasizes expansion and dispatchability rather than fixed-topology ESS-benefit attribution and voltage-exposure assessment
Zhou et al. (2023)	Renewable/load sampling combined with contingency uncertainty	Expansion-cost and reliability optimization through bilevel joint planning of network assets, DG, and ESS	Seasonal/time-block expansion-planning case study	Network assets and topology change with investment decisions, so a common topology is not preserved across ESS plans
Yuan et al. (2024)	Two-stage stochastic-robust treatment with representative scenarios and a worst-case uncertainty set	Minimization of distributed ESS investment and operating costs using MISOCP subproblems and metaheuristic/global search	17-node test system	Emphasizes worst-case robustness; validation is smaller in scale and does not include common-topology cross-year assessment
Carpinelli et al. (2024)	Uncertain inputs handled through multi-objective trade-off and risk analysis	Multi-objective BESS siting/sizing in a network-constrained unbalanced active distribution system	IEEE unbalanced test system	Targets a robust multi-objective compromise rather than fixed-topology bilevel attribution of ESS effects
Zhan et al. (2025)	Renewable variability embedded in stochastic dynamic operation	Dynamic ADN operation through coordinated reconfiguration, SOP/ESS control, learning-based topology decisions, and lower-level SOCP	Modified IEEE 33- and 69-bus systems	Uses adaptive topology, whereas the present study fixes the reference topology to isolate ESS effects
**This study**	Historical Year A/Year B load-PV-wind records; input-risk-stratified historical-day reduction; CVaR-based diagnostic tail-risk evaluation	Daily-equivalent TOTEX minimization with separate voltage-security assessment; upper-level discrete ESS siting/sizing and lower-level fixed-topology MISOCP with DistFlow-SOCP network physics; PSO/GA/DE used as search benchmarks	33-bus main study with cross-year and 7-day interday robustness checks; released 240-bus engineering-scale study	Common-topology, auditable attribution of ESS economic and physical-security effects across historical uncertainty, multiple horizons, and network scales

## 2. Materials and methods

### 2.1. System description and framework overview

The framework is demonstrated on a modified IEEE 33-bus active distribution network and evaluated on a released 240-bus feeder. Both systems use a common fixed-topology protocol: a reference radial topology is identified or reconstructed once and is then held unchanged across the compared ESS strategies.

Fig 1 summarizes the historical-data-driven planning workflow.

**Fig 1:**
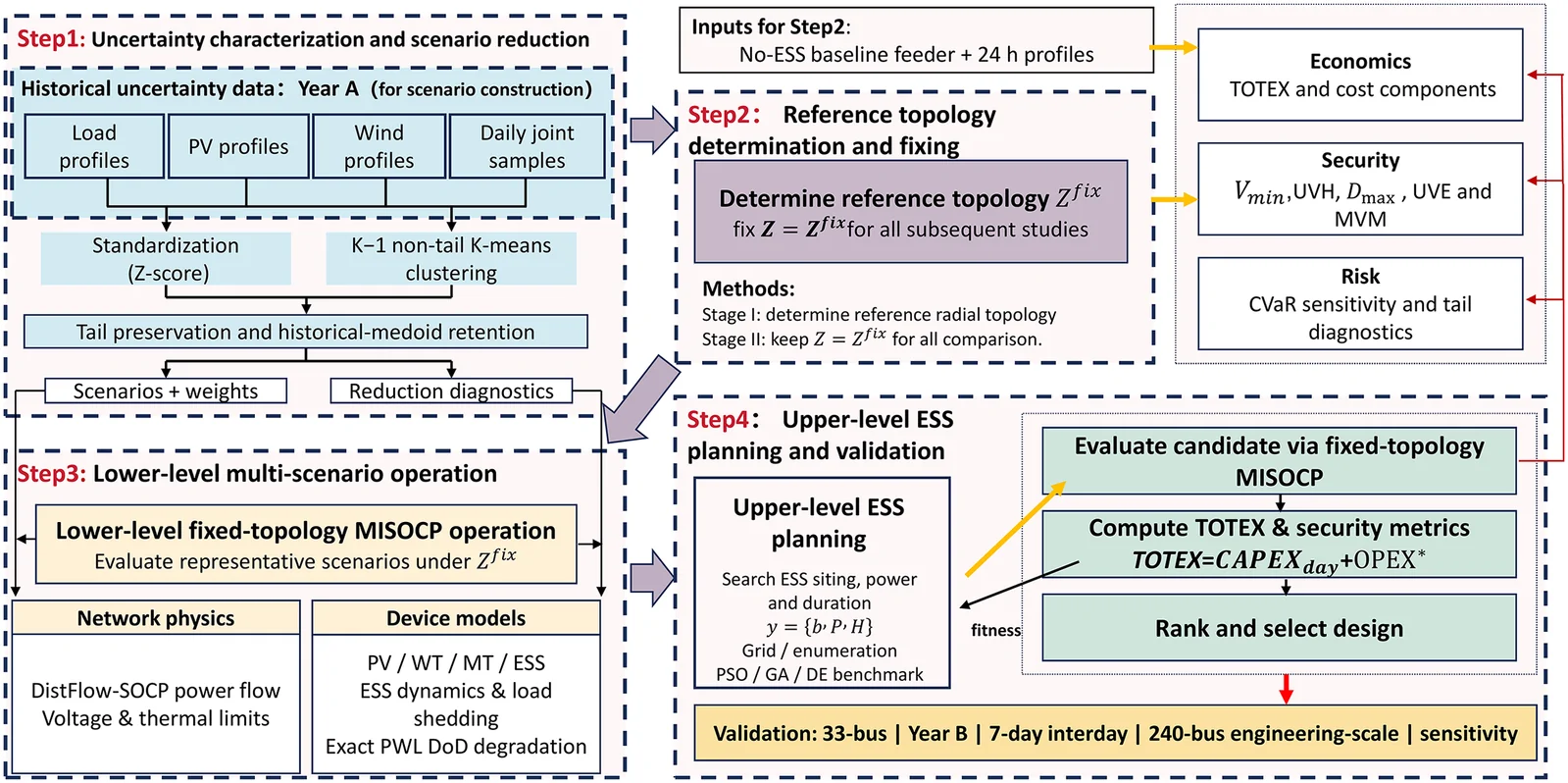
Overview of the uncertainty-aware bilevel ESS planning framework with fixed-topology DistFlow-SOCP network physics and MISOCP operational evaluation.

The workflow proceeds in four stages. First, uncertainty-aware scenario generation constructs representative operating conditions over the 24 h horizon. Second, a baseline no-ESS reconfiguration stage identifies the fixed reference topology. Third, the fixed-topology lower-level MISOCP evaluates multi-scenario feeder operation, combining DistFlow-SOCP network physics with exact PWL DoD degradation and other device constraints. Finally, the upper-level optimizer determines ESS siting and sizing by minimizing TOTEX through the lower-level operating consequences. This sequence links uncertainty representation, fixed-topology operation, and bilevel ESS planning without changing the comparison topology.

#### 2.1.1. Test system, baseline feeder, and ADN construction.

The case studies are conducted on the classical IEEE 33-bus radial distribution feeder reported by Baran and Wu [16], which is widely used for distribution-level operation, voltage-security, and reconfiguration studies. A one-time no-ESS topology-identification step is first performed over the 24 h horizon, and the resulting radial topology is used as the fixed reference topology for all subsequent ablation and ESS planning cases.

Fig 2 shows the one-time topology identification and the fixed radial topology used in all subsequent 33-bus comparisons.

**Fig 2:**
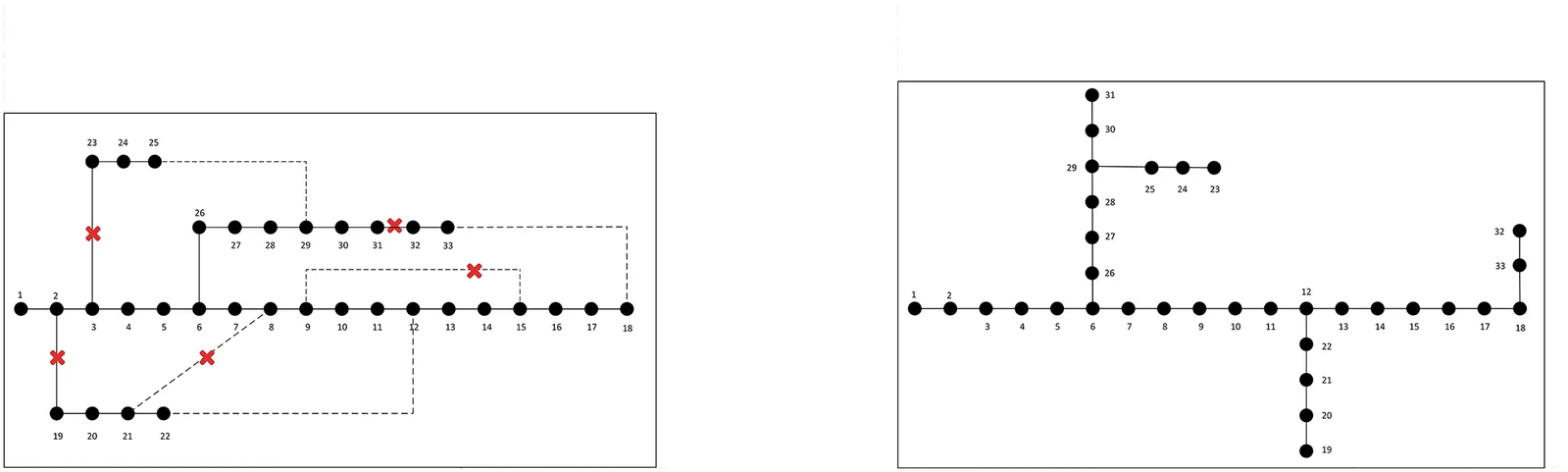
One-time topology identification for the IEEE 33-bus feeder: (a) initial feeder and switching actions; (b) fixed radial topology used in subsequent simulations.

To standardize the subsequent power-flow calculation and voltage-security assessment, the feeder is modeled in a per-unit framework. The base voltage and base apparent power are set to $V_{base}=\text{1}\text{2}.\text{6}\text{6}\text{ kV}$ and $S_{base}=\text{1}\text{0}\text{ MVA}$, respectively. Under the benchmark loading condition, the total active and reactive demands are $P_{load}=\text{3}\text{7}\text{1}\text{5}\text{ kW}$ and $Q_{load}=\text{2}\text{3}\text{0}\text{0}\text{ kVAr}$. All branch parameters and nodal demand data follow the standard IEEE 33-bus benchmark specification [16], which ensures that the underlying network physics remains transparent, reproducible, and directly comparable with prior distribution-system studies.

The study uses a fixed-topology attribution protocol. The baseline radial topology is identified once under the no-ESS condition and then kept unchanged for all multi-period evaluations, ADN ablations, and ESS planning experiments. This prevents moving baselines when strategies are compared, so economic and security differences can be attributed mainly to resource coordination and storage decisions. It also avoids repeatedly adding reconfiguration-related combinatorial complexity to the lower-level evaluator and upper-level planning loop. Unless otherwise stated, labels such as passive feeder, Base A, and Base B refer to enabled active-network resources rather than topology changes.

The passive IEEE 33-bus feeder is upgraded by adding PV units at buses 6, 19, and 31, a wind turbine at bus 18, microturbines at buses 25 and 33, PHEV demand at bus 30, an SVC at bus 9, and capacitor banks at buses 22 and 25. The seven ESS candidate buses are 18, 22, 24, 25, 30, 32, and 33.

Fig 3 shows the modified 33-bus ADN and the seven candidate buses.

**Fig 3:**
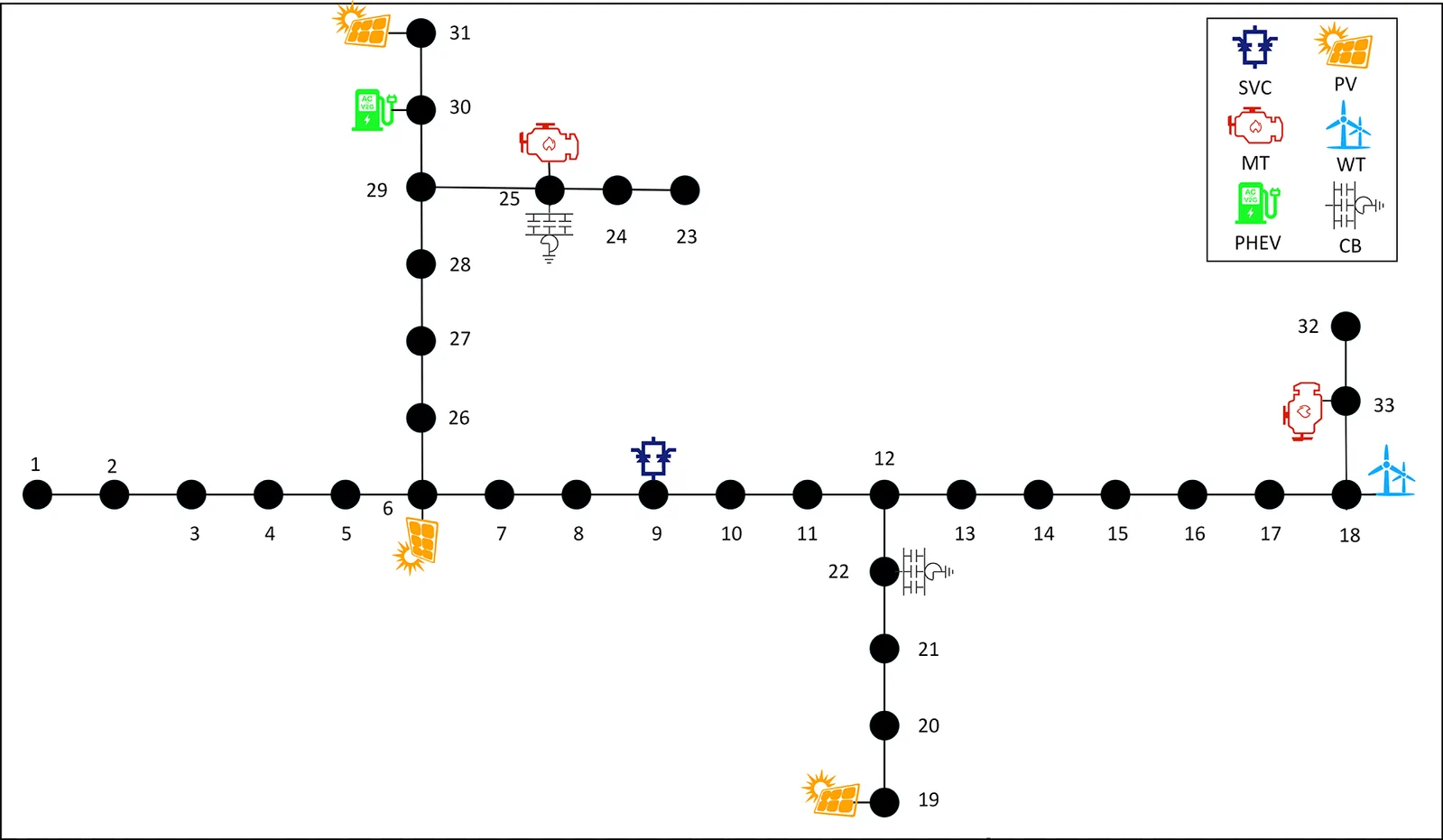
Modified IEEE 33-bus ADN with renewable units, flexible demand, dispatchable resources, reactive-power support devices, and ESS candidate buses.

#### 2.1.2. Historical data, baseline profiles, TOU tariff, and planning parameters.

Engineering-scale case study uses the released real-utility 240-bus test feeder reconstructed from its published bus and branch data [19]. The balanced positive-sequence equivalent contains 240 buses and 239 operating branches. Sixteen pre-specified candidate locations are used in the reported screen. Fig 4 uses identical coordinates in both panels to distinguish the benchmark topology from the ESS planning configuration.

**Fig 4:**
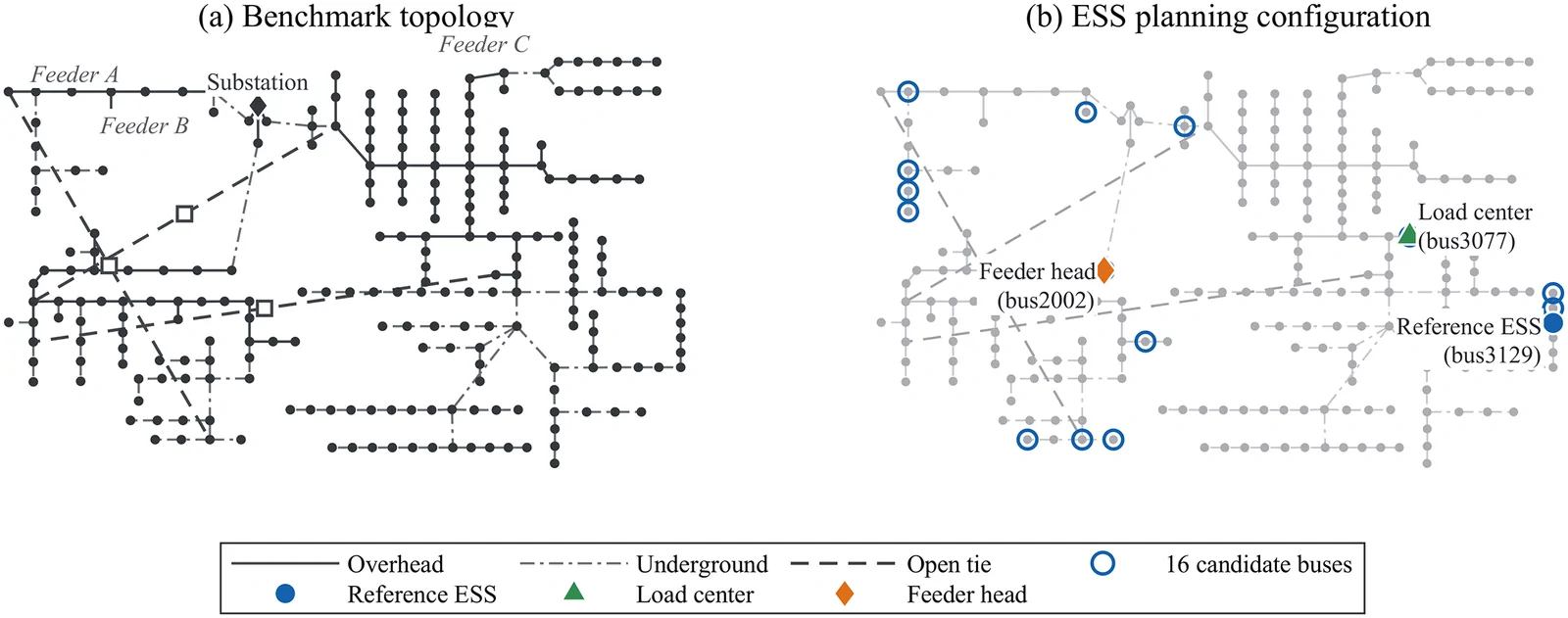
Released 240-node benchmark topology and ESS planning configuration. Both panels use identical released feeder coordinates. Panel (b) shows the 16 final candidate buses, Reference ESS at bus3129, load center at bus3077, and feeder head at bus2002.

Historical uncertainty inputs are taken from the Open Power System Data time-series package for Denmark [20]. Year A is 2016 and Year B is 2017. After UTC alignment and complete-day screening, each day is represented by one 72-dimensional joint vector containing 24 hourly load, 24 PV, and 24 wind values. Year A supports scenario construction and scaling; Year B is reserved for cross-year robustness check. Scenario construction uses only the retained historical observations; no generated trajectories are added.

Fig 5 reports the hourly means and 25th-75th percentile ranges calculated independently from the daily observations in each year. The same time-of-use tariff is used in all comparisons.

**Fig 5:**
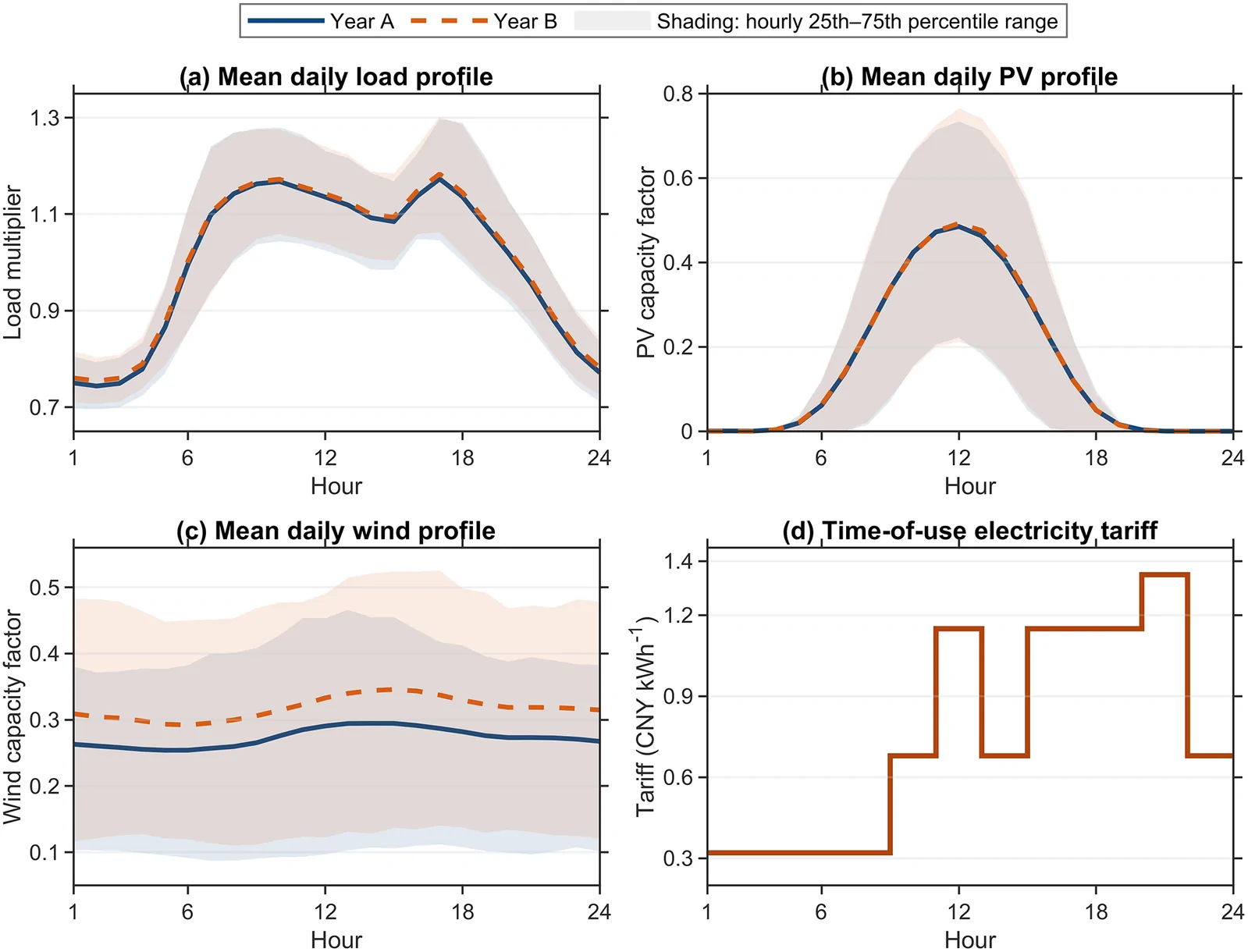
Mean daily Year A and Year B load, PV, and wind profiles and the time-of-use tariff. Shaded areas are hourly 25th–75th percentile ranges.

#### 2.1.3. Voltage-security thresholds and numerical acceptance.

The 0.95 p.u. threshold is used for the ADN-retrofitting and basic-feasibility diagnostic, whereas 0.97 p.u. is the stricter planning assessment threshold. A 0.96 p.u. case is included in the threshold sensitivity. Voltage penalties and load shedding remain feasibility backstops; conclusions are interpreted with penalty-independent physical metrics. Post-solution absolute physical residuals greater than ${\text{10}}^{-\text{3}}$ are classified as failures, while residuals at or below ${\text{10}}^{-\text{3}}$ are numerically acceptable.

### 2.2. Lower-level operational model

Given the common feeder configuration, deterministic baseline inputs, and the fixed reference topology $Z^{fix}$established in **the System description and framework overview subsection**, the lower-level model determines the multi-period dispatch of the ADN under a reduced set of representative uncertainty scenarios [21,22]. The model is formulated over a 24 h horizon and explicitly captures the coupling among network physics, heterogeneous controllable resources, and scenario-dependent renewable/load realizations. The feeder network physics are represented by a fixed-topology DistFlow-SOCP formulation, while the network-state and standard device-dispatch variables are continuous. The exact PWL DoD degradation representation introduces auxiliary binary segment-selection variables; consequently, the complete lower-level operational problem is a mixed-integer second-order cone program (MISOCP). These auxiliary binary variables arise from the degradation model rather than feeder reconfiguration, and the feeder topology remains fixed throughout all ESS candidate evaluations. Let t∈T={1,…,T} denote the hourly scheduling interval with T=24, k∈K={1,…,K} denote representative scenarios with probability $\pi_{k}$ satisfying $\sum_{k\in K}\pi_{k}=\text{1}$, i∈N denote buses, and (i,j)∈L denote branches. On this basis, the lower-level problem minimizes the expected operating expenditure while enforcing uncertainty-aware operational constraints on the common fixed feeder structure.

#### 2.2.1. Objective function and cost/emission/risk accounting.

This subsection formulates the scenario-based operating objective of the active distribution network (ADN) over a 24-h horizon. Under renewable-generation and demand uncertainties, the lower-level dispatch is designed to minimize the probability-weighted expected operating expenditure (OPEX), while explicitly monetizing carbon emissions and embedding security-oriented soft constraints to preserve mathematical feasibility under stressed realizations [22–25]. For each representative scenario k∈K with probability $\pi_{k}$, where $\sum_{k\in K}\pi_{k}=\text{1}$, the dispatch yields a scenario-wise operating cost $J_{k}$. The lower-level objective is therefore expressed as


$$\begin{array}{c} min\text{OPEX}=\sum_{k\in K}\;\pi_{k}J_{k} \end{array}$$ (1)

$$\begin{array}{c} J_{k}=C_{k}^{\text{grid}}+C_{k}^{\text{mt}}+C_{k}^{\text{deg}}+C_{k}^{\text{co}\text{2}}+C_{k}^{\text{volt}}+C_{k}^{\text{shed}} \end{array}$$ (2)

In Equation (2), $C_{k}^{\text{grid}}$, $C_{k}^{\text{mt}}$, and $C_{k}^{\text{deg }}$denote the upstream electricity procurement cost, microturbine (MT) operating cost, and ESS degradation cost, respectively [26,27]; $C_{k}^{\text{co}\text{2}}$is the monetized carbon-emission cost; and $C_{k}^{\text{volt}}$and $C_{k}^{\text{shed}}$are penalty terms associated with voltage-limit violations and involuntary load shedding [17,28]. All monetary terms are measured in Chinese Yuan (CNY). Power variables are expressed in MW, and with Δt=1h, all energy-related quantities are consistently evaluated in MWh. This unified monetary formulation is intentionally adopted so that routine operating costs, environmental externalities, and security-driven feasibility costs can be assessed within a common economic framework and subsequently interfaced with the upper-level TOTEX-based planning problem [18,23,25].

Economic operating costs are first quantified through electricity procurement under a time-of-use (TOU) tariff. This term provides the main market signal for peak shaving and valley filling, allowing operational flexibility to be valued economically [23,29]. For each scenario, the grid-purchase cost is given by


$$\begin{array}{c} C_{k}^{\text{grid}}=\sum_{t\in T}\;c_{t}^{\text{TOU}}P_{t,k}^{g}\Delta t \end{array}$$ (3)

The hourly time index, upstream grid import, and TOU price are defined in the notation following Equation (3). Consistent with **the System description and framework overview subsection**, the tariff has four levels: valley, flat, peak, and critical-peak prices of 320, 680, 1150, and 1350 CNY/MWh, respectively. The tariff introduces temporal price contrast so that storage, local generation, and flexible demand can be evaluated under stressed hours as well as routine hours. When dispatchable MT units are available, their operating cost is modeled in an energy-proportional form as


$$\begin{array}{c} C_{k}^{\text{mt}}=\sum_{t\in T}\;c^{\text{mt}}P_{t,k}^{\text{mt}}\Delta t \end{array}$$ (4)

where $P_{t,k}^{\text{mt}}$is the MT active power output (MW) and $c^{\text{mt}}$denotes the marginal MT generation cost (CNY/MWh). This linear cost representation is adopted to preserve tractability while retaining the essential economic role of dispatchable local support under stressed operating conditions [25,30].

The corresponding scenario-wise ESS degradation cost is expressed as


$$\begin{array}{c} d_{t,k}=SOC^{max}-SOC_{t,k}=\sum_{j=\text{1}}^{J}s_{j,t,k},\Phi \left(d_{t,k}\right)=\sum_{j=\text{1}}^{J}m_{j}s_{j,t,k},\;m_{j}=\frac{\Phi \left(D_{j}\right)-\Phi \left(D_{j-\text{1}}\right)}{D_{j}-D_{j-\text{1}}} \end{array}$$

$$\begin{array}{c} \Delta \varphi_{t,k}\geq \text{0},\;\Delta \varphi_{t,k}\geq \Phi \left(d_{t+\text{1},k}\right)-\Phi \left(d_{t,k}\right) \end{array}$$

$$\begin{array}{c} C_{k}^{deg}=c_{E}^{(rep)\left(\text{1}\text{0}\text{0}\text{0}E^{rated}\right)\sum_{t=\text{1}}^{T}\Delta \varphi_{t,k}} \end{array}$$ (5)

In Equation (5), $d_{t,k}$ is the depth of discharge relative to the maximum state of charge, $s_{j,t,k}$ is the incremental depth assigned to segment j, and $m_{j}$ is the marginal damage slope between adjacent DoD-damage knots $D_{j}$ and $\Phi (D_{j})$. The piecewise-linear representation is exact over the defined knot range. The nonnegative variable ${\Delta \varphi }_{t,k}$ records only positive hourly increases in the damage state. The scenario-wise degradation cost is obtained from the battery energy-block replacement price $c_{E}^{\text{rep}}$, rated energy $E^{\text{rated}}$, and the accumulated positive damage increments. The scenario probability $\pi_{k}$ is applied once outside this scenario-wise term; the capital-recovery factor is not applied to cycling damage, and degradation is not double-counted in CAPEX. The exact PWL implementation uses auxiliary binary segment-selection variables to enforce sequential filling of the active DoD-damage segment. These variables introduce mixed-integer structure into the complete lower-level model but do not alter the fixed feeder topology.

In addition to direct operating costs, this study explicitly monetizes carbon emissions so that the operational problem reflects both economic and environmental performance [24,25]. Since carbon emissions in the considered ADN arise primarily from upstream grid purchases and local MT generation, the scenario-wise carbon quantity is expressed as


$$\begin{array}{c} CO{\text{2}}_{k}=\gamma^{\text{grid}}\sum_{t\in T}\;P_{t,k}^{g}\Delta t+\gamma^{\text{mt}}\sum_{t\in T}\;P_{t,k}^{\text{mt}}\Delta t \end{array}$$ (6)

and the corresponding carbon-emission cost is calculated as


$$\begin{array}{c} C_{k}^{\text{co}\text{2}}=p^{\text{co}\text{2}}CO{\text{2}}_{k} \end{array}$$ (7)

Here, the carbon price is set to 100 CNY/t, and the emission factors represent upstream grid electricity and MT generation. This carbon term avoids evaluating dispatch solely by energy cost and keeps the cost model consistent with low-carbon ADN operation [24,25].


$$\begin{array}{c} C_{k}^{\text{volt}}=w_{v}\sum_{t\in T}\;\sum_{i\in N}\;\left(s_{i,t,k}^{\text{min}}+s_{i,t,k}^{\text{max}}\right)\Delta t \end{array}$$ (8)

and the load-shedding penalty is written as


$$\begin{array}{c} C_{k}^{\text{shed}}=\text{VoLL}\sum_{t\in T}\;\sum_{i\in N}\;P_{i,t,k}^{\text{shed}}\Delta t \end{array}$$ (9)

In Equations (8),(9), $s_{i,t,k}^{\text{min}}$ and $s_{i,t,k}^{\text{max}}$are the lower- and upper-voltage slack variables at bus i, respectively, and $P_{i,t,k}^{\text{shed}}$is the involuntary curtailed demand (MW). The voltage-penalty coefficient is set to $w_{v}={\text{1}\text{0}}^{\text{5}}$, and the value of lost load is set to $\text{VoLL}={\text{1}\text{0}}^{\text{5}}$ CNY/MWh. These large coefficients are intentionally selected to ensure that voltage violations and load curtailment remain last-resort actions, thereby preserving both numerical robustness and the physical credibility of the optimized dispatch. Collectively, Equations (1)-(9) define a lower-level operating objective in which normal operating expenditures, environmental costs, and security-related feasibility costs are internalized in a unified monetary form.

#### 2.2.2. Historical uncertainty modeling and input-risk-stratified scenario reduction.

Scenario construction uses all $N_{A}=\text{ 366}$ complete Year A days described in **the Historical data, baseline profiles, TOU tariff, and planning parameters subsection**. Load, PV, and wind remain aligned within each historical day; all scaling parameters are estimated from Year A only.


$$\begin{array}{c} x_{d}={\left[L_{d,\text{1}},\ldots ,L_{d,\text{2}\text{4}},PV_{d,\text{1}},\ldots ,PV_{d,\text{2}\text{4}},W_{d,\text{1}},\ldots ,W_{d,\text{2}\text{4}}\right]}^{T},\;d=\text{1},\ldots ,N_{A},\;N_{A}=\text{3}\text{6}\text{6} \end{array}$$ (10)

Equation (10) defines the 72-dimensional joint historical vector. L, PV, and W denote the hourly load multiplier and renewable capacity factors for the same UTC day d.


$$\begin{array}{c} x_{d,r,t}^{*}=\frac{x_{d,r,t}-\mu_{r,t}}{s_{r}},s_{r}=\sqrt{\frac{\text{1}}{\text{2}\text{4}}\sum_{t=\text{1}}^{\text{2}\text{4}}\sigma_{r,t}^{\text{2}}},r\in \left\{L,PV,W\right\} \end{array}$$ (11)

Equation (11) centers each hour within a Load/PV/Wind block using the Year A hour-specific mean and applies one block-wide scale based on the 24 hourly population standard deviations. The three 24-hour blocks therefore enter the profile distance with equal weight.


$$\begin{array}{c} S_{d}=\text{0}.\text{3}\text{0}z\left(p_{d}^{net}\right)+\text{0}.\text{2}\text{0}z\left(l_{d}^{ren}\right)+\text{0}.\text{2}\text{0}z\left(\rho_{d}\right)+\text{0}.\text{3}\text{0}z\left(v_{d}^{proxy}\right) \end{array}$$ (12)

Equation (12) defines the combined input-risk score from standardized peak net load, low renewable availability, maximum absolute net-load ramp, and the fixed-topology voltage-stress proxy. All four features are computed from Year A inputs; no optimization outcome is used.


$$\begin{array}{c} H_{s}=Top_{\left\lceil \text{0}.\text{1}\text{0}N_{s}\right\rceil }S_{d},d\in D_{s} \end{array}$$ (13)

Equation (13) defines the high-risk pool separately for each season as the ⌈0.10 $N_{s}$⌉ historical days with the highest combined input-risk scores, where $N_{s}$ denotes the number of complete historical days in season s.


$$\begin{array}{c} D(d,q)=\sqrt{\text{0}.\text{7}\text{0}\cdot \frac{\text{1}}{\text{3}}\sum_{r\in \left\{L,PV,W\right\}}MSE\left(x_{d,r}^{*},x_{q,r}^{*}\right)+\text{0}.\text{3}\text{0}\cdot \frac{\text{1}}{\text{4}}\sum_{m=\text{1}}^{\text{4}}{\left(z_{d,m}-z_{q,m}\right)}^{\text{2}}} \end{array}$$ (14)

Equation (14) combines the equally weighted Load/PV/Wind block mean-squared-error distance with the four-feature risk distance. The 0.70/0.30 weights were fixed before representative-day selection.


$$\begin{array}{c} R_{K}=Select_{K}\left(D,\left\{D_{s},H_{s}\right\},S,H,R\right),K\in \left\{\text{3},\text{5},\text{1}\text{0},\text{2}\text{0}\right\} \end{array}$$ (15)

Equation (15) summarizes the predeclared representative-set rules. K3 uses three global medoids. K5 uses one ordinary medoid per season plus the annual combined-input-risk maximum. K10 uses one ordinary and one high-risk medoid per season plus the annual high-load/low-renewable and annual high-ramp maxima. K20 retains the K10 core and adds ten constrained seasonal ordinary medoids using the frozen Hamilton allocation.


$$\begin{array}{c} a(d)=\text{arg}\underset{k\in E_{K}(d)}{\text{min}}D\left(d,r_{k}\right) \end{array}$$ (16)

Equation (16) assigns each historical day exactly once to the nearest eligible representative under D. For K5, high-risk days use the annual risk representative and other days use their seasonal ordinary medoid; for K10/K20, days compete within the same season and risk stratum. Ties and duplicate replacements are deterministic.


$$\begin{array}{c} \pi_{k}=\frac{n_{k}}{N_{A}}=\frac{n_{k}}{\text{3}\text{6}\text{6}},n_{k}=\left|\left\{d:a(d)=k\right\}\right| \end{array}$$ (17)

In Equation (17), $n_{k}$ is the assigned membership count of representative k. Thus each probability is the empirical membership count divided by 366; no probability floor or manual inflation is applied.


$$\begin{array}{c} \sum_{k=\text{1}}^{K}\pi_{k}=\text{1},\pi_{k}\geq \text{0} \end{array}$$ (18)

Equation (18) conserves the full historical probability mass. No response or voltage outcome is used as a selection oracle: 21 January 2016 emerges from the frozen input-side rules and is retained exactly in K5, K10, and K20 but not K3. Full-history economic and voltage-security audits are conducted after selection.


$$\begin{array}{c} CVaR_{\alpha (L)}=\underset{\eta }{\text{min}}\left[\eta +\frac{\text{1}}{\text{1}-\alpha }\sum_{k=\text{1}}^{K}\pi_{k}{\left[L_{k}-\eta \right]}_{+}\right] \end{array}$$ (19)

In Equation (19), $L_{k}$ is the scenario-wise daily operating loss $J_{k}$ used in the risk diagnostic, including the operating, carbon, voltage-backstop, and load-shedding terms defined in Equations (2)-(9). CVaR is reported only as a sensitivity diagnostic; it is not the principal planning objective used to select the retained design.

K = 10 is used for the principal 33-bus planning and strategy comparisons, and K = 20 provides a higher-fidelity scenario-set check. K = 3 and K = 5 are scenario-reduction sensitivity cases rather than separate full-domain planning optimizations. Fidelity is assessed against full-history TOTEX, UVE, and minimum-voltage outcomes, together with exact worst-day-retention and runtime checks.

#### 2.2.3. Operational constraints.

The OPEX defined in **the Objective function and cost/emission/risk accounting subsection** is minimized subject to a set of constraints that jointly enforce feeder physics, device-level feasibility, inter-temporal energy coupling, and security-oriented operating limits [30,31]. In the proposed framework, these constraints are organized according to the physical logic of ADN operation rather than by device type alone. Specifically, the feasible set is constructed in five layers: topology treatment, network power-flow equations, unified nodal injections and device output limits, flexible-demand and ESS dynamics, and finally security-preserving soft constraints. Such an organization is adopted to make explicit how uncertainty scenarios are translated into physically admissible dispatch decisions on the common fixed feeder structure $Z^{fix}$. As established in **the System description and framework overview subsection**, feeder reconfiguration is performed only once in the baseline no-ESS stage to identify the reference topology, after which all subsequent multi-scenario dispatch evaluations are carried out under fixed topology. Accordingly, the topology-related constraints below should be interpreted as a one-time identification step followed by fixed-topology operation.

(a) **Stage-I topology identification and radiality enforcement**

To identify the fixed reference feeder used in the later dispatch and planning studies, a radial topology is determined once under the baseline condition without ESS installation. For this one-time identification step, a standard single-commodity-flow (SCF) formulation is adopted [16,30,32], with binary branch-status variables $z_{ij}\in \{\text{0},\text{1}\}$ and fictitious flow variables $f_{ij}$. The radiality constraints are expressed as


$$\begin{array}{c} \sum_{(i,j)\in L}\;z_{ij}=|𝒩|-\text{1} \end{array}$$

$$\begin{array}{c} -M_{\text{topo}}z_{ij}\leq f_{ij}\leq M_{\text{topo}}z_{ij},\forall (i,j)\in L \end{array}$$

$$\begin{array}{c} \sum_{(m,i)\in L}\;f_{mi}-\sum_{(i,n)\in L}\;f_{in}=\text{1},\forall i\in N\setminus \left\{\text{1}\right\} \end{array}$$

$$\begin{array}{c} \sum_{(\text{1},n)\in L}\;f_{\text{1}n}-\sum_{(m,\text{1})\in L}\;f_{m\text{1}}=|𝒩|-\text{1} \end{array}$$ (20)

where N and L denote the sets of buses and branches, respectively; bus 1 is the root (slack) bus; $f_{ij}$is the fictitious SCF variable; and $M_{\text{topo}}$is a sufficiently large constant. These SCF constraints are used only in Stage I to identify the baseline radial reference topology $Z^{fix}$. Once $Z^{fix}$is obtained, all later multi-scenario operational evaluations and bilevel planning studies are carried out under the fixed-topology condition $Z=Z^{fix}$.

(b) **Stage-II DistFlow branch-flow model and SOCP relaxation**

Given the fixed topology $Z^{fix}$obtained in Stage I, the feeder operation under each representative scenario k∈K and each hourly interval t∈T is modeled by the DistFlow branch-flow equations with squared voltage magnitudes. Let $P_{ij,t,k}$and $Q_{ij,t,k}$denote the active and reactive power flows on branch (i,j), let $U_{i,t,k}=|V_{i,t,k}{|}^{\text{2}}$denote the squared voltage magnitude at bus i, and let ${\text{𝓁}}_{ij,t,k}=|I_{ij,t,k}{|}^{\text{2}}$denote the squared branch current magnitude. The voltage-drop relationship is expressed as


$$\begin{array}{c} U_{j,t,k}=U_{i,t,k}-\text{2}\left(r_{ij}P_{ij,t,k}+x_{ij}Q_{ij,t,k}\right)+\left(r_{ij}^{\text{2}}+x_{ij}^{\text{2}}\right){\text{𝓁}}_{ij,t,k},\forall (i,j)\in L,\forall t,\forall \text{k} \end{array}$$ (21)

For each non-root bus i∈N∖{1}, nodal power balance is enforced by equating the incoming power net of ohmic losses to the outgoing power plus the net injection, i.e.,


$$\begin{array}{c} \sum_{m:(m,i)\in L}\;P_{mi,t,k}-\sum_{j:(i,j)\in L}\;P_{ij,t,k}-r_{mi}{\text{𝓁}}_{mi,t,k}=p_{i,t,k}^{\text{inj}},\forall i\in N,\forall t\in T,\forall k\in K \end{array}$$ (22)

$$\begin{array}{c} \sum_{m:(m,i)\in L}\;Q_{mi,t,k}-\sum_{j:(i,j)\in L}\;Q_{ij,t,k}-x_{mi}{\text{𝓁}}_{mi,t,k}=q_{i,t,k}^{\text{inj}},\forall i\in N,\forall t\in T,\forall k\in K \end{array}$$ (23)

The nonconvex equality $P_{ij,t,k}^{\text{2}}+Q_{ij,t,k}^{\text{2}}=U_{i,t,k}{\text{𝓁}}_{ij,t,k}$is relaxed into a second-order cone constraint [17,32,33],


$$\begin{array}{c} {\|\left[\begin{array}{c} \text{2}P_{ij,t,k}\\ \text{2}Q_{ij,t,k}\\ U_{i,t,k}-{\text{𝓁}}_{ij,t,k} \end{array}\right]\|}_{\text{2}}\leq U_{i,t,k}+{\text{𝓁}}_{ij,t,k},\forall (i,j)\in L,\forall t,k \end{array}$$ (24)

The SOCP relaxation preserves the radial feeder’s branch-flow structure and remains the network-physics component of the complete Stage-II MISOCP used for repeated bilevel evaluations.

(c) **Unified nodal injections and DER/reactive-resource constraints.**

To close the branch-flow equations, the net active and reactive injections at each bus are assembled by superposing all device contributions and subtracting the post-curtailment demand. A unified nodal injection representation is adopted so that the feeder equations remain structurally unchanged across different resource placements [22,23,33]. Specifically,


$$\begin{array}{c} p_{i,t,k}^{\text{inj}}=-\left(P_{i,t}^{d}-P_{i,t,k}^{\text{shed}}\right)+a_{i}^{pv}P_{i,t,k}^{pv}+a_{i}^{w}P_{i,t,k}^{w}+a_{i}^{mt}P_{i,t,k}^{mt}+a_{i}^{ess}\left(P_{t,k}^{dis}-P_{t,k}^{ch}\right)+a_{i}^{phev}P_{t,k}^{phev} \end{array}$$

$$\begin{array}{c} q_{i,t,k}^{\text{inj}}=-\left(Q_{i,t}^{d}-Q_{i,t,k}^{\text{shed}}\right)+a_{i}^{pv}Q_{i,t,k}^{pv}+a_{i}^{w}Q_{i,t,k}^{w}+a_{i}^{mt}Q_{i,t,k}^{mt}+a_{i}^{svc}Q_{t,k}^{svc}+a_{i}^{cb}Q_{i,t,k}^{cb}+a_{i}^{ess}Q_{t,k}^{ess} \end{array}$$ (25)

In Equation (25), $p_{i,t,k}^{\text{inj}}$ and $q_{i,t,k}^{\text{inj}}$ are the net active and reactive injections at bus i, hour t, and scenario k. $P_{i,t}^{\text{d}}$ and $Q_{i,t}^{\text{d}}$ are demand; $P_{i,t,k}^{\text{shed}}$ and $Q_{i,t,k}^{\text{shed}}$ are curtailed demand; the superscripts pv, w, mt, phev, svc, cb, and ess identify the corresponding device outputs; and each $a_{i}$ term is the fixed location indicator for that device. Charging enters as a withdrawal and discharging as an injection. The single wind unit at bus 18 is indexed by hour and scenario and is mapped to the bus balance through its location indicator.


$$\begin{array}{c} \text{0}\leq P_{i,t,k}^{\text{pv}}\leq {\bar{P}}_{i,t,k}^{\text{pv}},Q_{i,t,k}^{\text{pv}}=P_{i,t,k}^{\text{pv}}\text{tan}(\varphi_{\text{pv}}),\forall i,t,k \end{array}$$ (26)

$$\begin{array}{c} \text{0}\leq P_{t,k}^{w}\leq {\bar{P}}_{t,k}^{w},Q_{t,k}^{w}=P_{t,k}^{w}\text{tan}(\varphi_{w}),\forall t,k \end{array}$$ (27)

$$\begin{array}{c} \text{0}\leq P_{i,t,k}^{\text{mt}}\leq {\bar{P}}_{i}^{\text{mt}},Q_{i,t,k}^{\text{mt}}=P_{i,t,k}^{\text{mt}}\text{tan}(\varphi_{\text{mt}}),\forall i,t,k \end{array}$$ (28)

In Equations (26)–(28), $P_{i,t,k}^{\text{pv}}$, $P_{t,k}^{\text{w}}$, and $P_{i,t,k}^{\text{mt}}$ denote PV, wind, and microturbine active-power outputs. Their upper bounds are the scenario-dependent available renewable power or the installed dispatchable capacity. The corresponding reactive powers $Q_{i,t,k}^{\text{pv}}$, $Q_{t,k}^{\text{w}}$, and $Q_{i,t,k}^{\text{mt}}$ follow the stated fixed power-factor relations using $\text{tan}(\varphi_{\text{pv}})$, $\text{tan}(\varphi_{\text{w}})$, and $\text{tan}(\varphi_{\text{mt}})$, respectively, with the same bus, hour, and scenario indexing.

For feeder-level reactive support, the SVC is modeled as a bidirectional continuously adjustable reactive source. The capacitor bank is represented by a unidirectional step-relaxed reactive-support variable bounded between zero and its maximum step-equivalent value. Multiplication by the fixed step size gives the injected capacitive reactive power. This relaxation approximates switched capacitor support without adding integer variables to repeated multi-scenario evaluations.


$$\begin{array}{c} -{\bar{Q}}^{\text{svc}}\leq Q_{t,k}^{\text{svc}}\leq {\bar{Q}}^{\text{svc}},\forall t,k \end{array}$$ (29)

$$\begin{array}{c} \text{0}\leq g_{i,t,k}^{cb}\leq g_{i}^{max},\;Q_{i,t,k}^{cb}=g_{i,t,k}^{cb}\Delta Q_{i}^{cb},\;\forall i,t,k \end{array}$$ (30)

These constraints define the active-power availability and reactive-support capability needed to close the nodal power balances.

(d) **PHEV active-only flexibility and ESS dynamics**

The aggregated PHEV resource is modeled as a bounded active-power flexibility term within its charging/discharging capability [34,35],


$$\begin{array}{c} -{\bar{P}}^{\text{phev}}\leq P_{t,k}^{\text{phev}}\leq {\bar{P}}^{\text{phev}},\forall t,k \end{array}$$ (31)

The aggregated PHEV resource is modeled as active-power flexibility only, and its reactive-power contribution is neglected. This simplification is consistent with the case design, where PHEVs represent shiftable charging demand at bus 30 and reactive support is supplied by SVC, CB, and inverter-interfaced DERs.

The ESS is modeled with charging/discharging power, reactive capability, and inter-temporal energy conservation [21,26,36]. Let $P_{t,k}^{\text{ch}}$, $P_{t,k}^{\text{dis}}$, and $Q_{t,k}^{\text{ess}}$denote the charging, discharging, and reactive powers, $\eta_{ch}$and $\eta_{dis}$denote the charging and discharging efficiencies, respectively, and let $E_{t,k}$denote the stored energy. The corresponding feasibility constraints are expressed as


$$\begin{array}{c} \text{0}\leq P_{t,k}^{\text{ch}}\leq P_{max},\text{0}\leq P_{t,k}^{\text{dis}}\leq P_{max},\forall t,k \end{array}$$

$$\begin{array}{c} E_{t+\text{1},k}=E_{t,k}+\eta_{ch}P_{t,k}^{ch}\Delta t-\frac{\text{1}}{\eta_{dis}}P_{t,k}^{dis}\Delta t,\forall t=\text{1},\ldots ,T-\text{1},\forall k \end{array}$$

$$\begin{array}{c} E^{min}\leq E_{t,k}\leq E^{max},\forall t,k \end{array}$$

$$\begin{array}{c} E_{T,k}=E_{\text{1},k},\forall k \end{array}$$

$$\begin{array}{c} {\left(P_{t,k}^{\text{dis}}-P_{t,k}^{\text{ch}}\right)}^{\text{2}}+{\left(Q_{t,k}^{\text{ess}}\right)}^{\text{2}}\leq S_{\text{max}}^{\text{2}},\forall t,k \end{array}$$ (32)

Here, the terminal condition $E_{T,k}=E_{\text{1},k}\;$is explicitly enforced to ensure a fair daily comparison by preventing the optimizer from exploiting end-of-horizon energy depletion or accumulation. The remaining ESS constraints characterize charging/discharging bounds, inter-temporal energy conservation, and converter apparent-power capability, thereby ensuring physically admissible storage operation across the entire scheduling horizon.

(e) **Voltage-security-oriented soft constraints and load shedding**

To preserve model solvability under rare but severe stressed scenarios, voltage-security constraints are enforced using penalized slack variables rather than by allowing the optimization to fail [37–39]. Specifically, the squared bus voltages are constrained as


$$\begin{array}{c} (V^{min}{)}^{\text{2}}-s_{i,t,k}^{min}\leq U_{i,t,k}\leq (V^{max}{)}^{\text{2}}+s_{i,t,k}^{max} \end{array}$$

$$\begin{array}{c} s_{i,t,k}^{min}\geq \text{0},s_{i,t,k}^{max}\geq \text{0},\forall i,t,k \end{array}$$ (33)

while branch currents are bounded by


$$\begin{array}{c} \text{0}\leq {\text{𝓁}}_{ij,t,k}\leq {\bar{\text{𝓁}}}_{ij},\forall (i,j),t,k \end{array}$$ (34)

In Equation (34), $l_{i,j,t,k}$ is the squared current magnitude of branch (i,j) at hour t in scenario k, and its upper bound is the corresponding squared thermal-current limit. Involuntary load shedding is then allowed only as a reliability backstop,


$$\begin{array}{c} \text{0}\leq P_{i,t,k}^{\text{shed}}\leq P_{i,t}^{d},Q_{i,t,k}^{\text{shed}}=P_{i,t,k}^{\text{shed}}\text{tan}(\text{arccos }\phi_{i}^{d}),\forall i,t,k \end{array}$$ (35)

In Equation (35), $P_{i,t,k}^{\text{shed}}$ is bounded by the active demand $P_{i,t}^{\text{d}}$, and $Q_{i,t,k}^{\text{shed}}$ follows the demand power factor through $Q_{i,t,k}^{\text{shed}}=P_{i,t,k}^{\text{shed}}\text{ tan}(\text{arccos}(\varphi_{i}^{\text{d}}))$. Voltage slack variables and curtailed demand are feasibility safeguards, not normal operating resources; the large coefficients in **the Objective function and cost/emission/risk accounting subsection** keep them as last-resort backstops under rare stressed scenarios.

The operational constraints above define a tractable multi-scenario feasible set that combines fixed-topology feeder physics, controllable resources, ESS dynamics, and last-resort feasibility backstops. Together with the historical representative scenarios, they form the lower-level operational model evaluated for each upper-level ESS alternative.

### 2.3. Bilevel ESS planning model and solution methodology

In this study, bilevel denotes a nested planning-operation structure rather than a general-purpose bilevel optimization algorithm. The upper layer selects discrete ESS planning alternatives, while the lower layer evaluates the corresponding operational responses under the prescribed network constraints. For the reported 33-bus case, the finite planning domain is solved through exhaustive enumeration of candidate ESS configurations, and the optimal solution is identified according to the defined numerical criteria.

Under the fixed reference topology $Z^{fix}$established in **the System description and framework overview subsection**, the upper-level planning problem optimizes ESS siting and sizing decisions for the ADN. Different from the one-time baseline reconfiguration stage, the upper-level model no longer alters feeder structure, but instead evaluates candidate storage configurations on the common fixed feeder through repeated calls to the lower-level multi-scenario operational model described in **the Lower-level operational model subsection**. This treatment allows the planning layer to search over storage placement and capacity decisions while assessing each candidate solution under a structurally consistent network environment. Accordingly, the planning stage is carried out under the fixed-topology condition


$$\begin{array}{c} Z=Z^{fix} \end{array}$$ (36)

Under this condition, the upper-level decision specifies the installation bus, rated converter power, and storage duration. Energy capacity is determined by the product of rated power and duration.


$$\begin{array}{c} 𝐲=\{b,P_{max},E_{max}\},b\in N_{E} \end{array}$$ (37)

where b is the ESS installation bus selected from the candidate set $N_{E}\subseteq N$, $P_{\text{max}}\;$is the rated converter power (MW), and $E_{\text{max}}\;$is the energy capacity (MWh). To maintain engineering interpretability and avoid unrealistic power-energy decoupling, the ESS is designed using a fixed-duration rule,


$$\begin{array}{c} E_{max}=hP_{max} \end{array}$$ (38)

For the 33-bus study, duration is selected from 2, 3, and 4 h. Rated power is selected from 0.10 to 1.00 MW in 0.10 MW steps, and energy capacity equals rated power multiplied by duration.


$$\begin{array}{c} \text{0}\leq P_{max}\leq \bar{P},\text{0}\leq E_{max}\leq \bar{E} \end{array}$$ (39)

The seven candidate buses, ten power levels, and three duration classes give 210 positive-capacity plans. One explicit no-build alternative is added, producing the complete GRID211 domain summarized in Table 2.

**Table 2:** Upper-level ESS candidate domains for the IEEE 33-bus and released 240-bus systems.

System	Candidate locations	Power levels (MW)	Duration (h)	Evaluated alternatives
IEEE 33-bus	7 buses: 18, 22, 24, 25, 30, 32, 33	0.10–1.00 (0.10 step)	2, 3, 4	211
Released 240-bus	16 predefined buses	0.25, 0.50, 0.75, 1.00, 1.25, 1.60, 2.00	2, 4	155

Note: The 2 h family was evaluated at all 16 retained candidate locations (16 × 7 = 112 positive-capacity alternatives), whereas the 4 h family was evaluated only at the predefined six-location subset (6 × 7 = 42). Thus, 154 positive-capacity alternatives were evaluated; one no-build alternative gives 155 total alternatives. The retained planning domain excludes bus1002 and bus2006.

To place capital expenditure and daily operating expenditure on the same economic basis, the ESS investment cost is converted into a daily-equivalent capital term through the capital recovery factor (CRF). Let $c_{P}$(CNY/kW) and $c_{E}$(CNY/kWh) denote the unit costs of rated power and energy capacity, respectively. The equivalent daily capital expenditure is then expressed as


$$\begin{array}{c} CAPEX_{\text{day}}\left(P_{max},E_{max}\right)=\frac{\text{CRF}}{\text{3}\text{6}\text{5}}\left(\text{1}\text{0}\text{0}\text{0}c_{P}P_{max}+\text{1}\text{0}\text{0}\text{0}c_{E}E_{max}\right) \end{array}$$

$$\begin{array}{c} \text{CRF}=\frac{r(\text{1}+r{)}^{L}}{(\text{1}+r{)}^{L}-\text{1}} \end{array}$$ (40)

In Equation (40), the factor 1000 converts MW/MWh into kW/kWh for consistency with investment-unit costs. The discount rate, project lifetime, and cost coefficients are selected as planning-level assumptions for grid-side ESS and provide a transparent basis for comparing storage capacities against their investment burden.

The ESS CAPEX split is derived from the 2025 CNESA average LFP EPC bid prices for 2 h (1043.82 CNY/kWh) and 4 h (935.40 CNY/kWh) systems [40]. Under $T(H)=\frac{c_{P}}{H}+c_{E}$, the paired benchmarks give $c_{E}=\text{2}T(\text{4})-T(\text{2})=\text{8}\text{2}\text{6}.\text{9}\text{8}$ CNY/kWh and $c_{P}=\text{2}\left[T(\text{2})-c_{E}\right]=\text{4}\text{3}\text{3}.\text{6}\text{8}$ CNY/kW. The fixed O&M assumption and broader storage-cost context are benchmarked against the PNNL assessment [41]; the numerical inputs are case-study assumptions rather than universal market values.

Given a candidate planning decision 𝐲and the fixed topology $Z^{fix}$, the lower-level stochastic MISOCP operational model described in the Lower-level operational model subsection is solved under the stated numerical solution criteria under the representative scenario set constructed in **the Historical uncertainty modeling and input-risk-stratified scenario reduction subsection**, thereby yielding the resulting OPEX


$$\begin{array}{c} OPEX^{⋆}(𝐲)=\underset{𝐱\in \Omega (𝐲,Z^{fix})}{min}\;\sum_{k\in K}\;\pi_{k}J_{k} \end{array}$$ (41)

where 𝐱collects all lower-level operational variables, including branch flows, bus voltages, device dispatch setpoints, ESS schedules, voltage slack variables, and involuntary load shedding, and $\Omega (𝐲,Z^{fix})$ denotes the feasible region induced by the DistFlow-SOCP network model, device-level constraints, and scenario-wise security limits under the planning decision 𝐲. On this basis, the upper-level objective is formulated as the total daily expenditure,


$$\begin{array}{c} \underset{𝐲}{min}\;TOTEX(𝐲)=CAPEX_{\text{day}}(P_{\text{max}},E_{\text{max}})+OPEX^{⋆}(𝐲) \end{array}$$ (42)

Voltage-security performance is evaluated independently of monetary penalty terms using probability-weighted post-processing. For each bus, hour, and scenario, the scenario-specific feeder minimum, probability-weighted hourly minimum, undervoltage depth, and event indicator are defined as


$$\begin{array}{c} V_{min,t,k}=\underset{i\in N}{min}V_{i,t,k},{\bar{V}}_{min,t}=\sum_{k=\text{1}}^{K}\pi_{k}V_{min,t,k} \end{array}$$

$$\begin{array}{c} d_{t,k}=max\left(\text{0},V_{lim}-V_{min,t,k}\right),e_{t,k}=I\left(V_{min,t,k}<V_{lim}-\tau \right) \end{array}$$ (43)

where the evaluation threshold and numerical event tolerance follow Table 3, and each scenario retains its empirical probability. The expected undervoltage duration, maximum scenario-wise depth, and cumulative probability-weighted exposure are

**Table 3:** Key technical, economic, scenario, and computational parameters.

Parameter group	Value used in this study	Notes
Benchmark feeder	Modified IEEE 33-bus ADN based on the Baran–Wu feeder; $V_{base}$ = 12.66 kV; $S_{base}$ = 10 MVA; base demand = 3715 kW + 2300 kVAr.	The reference radial topology is identified once and then fixed for subsequent ESS comparisons.
Renewable and dispatchable resources	PV buses: 6, 19, 31; WT bus: 18; MT buses: 25, 33.	PV and WT represent uncertain renewable injections; MT units provide dispatchable local support.
Flexible and reactive resources	PHEV bus: 30; SVC bus: 9; capacitor-bank buses: 22, 25.	PHEV provides active-power flexibility; SVC and capacitor banks provide reactive/voltage support.
Released 240-bus case-study system	Balanced positive-sequence equivalent of the released real-utility 240-bus feeder; 240 buses and 239 fixed operating branches; 16 pre-specified ESS candidate locations.	Used for engineering-scale executability and siting assessment; the detailed candidate list is reported with the 240-bus study.
Historical uncertainty data	Open Power System Data (OPSD) time-series package; Denmark (DK). Year A: 2016; Year B: 2017; complete 24 h joint load/PV/wind daily samples.	Year A is used for scenario construction and scaling; Year B is reserved for cross-year robustness check.
33-bus ESS planning domain	Candidate buses: 18, 22, 24, 25, 30, 32, 33; $P_{ESS}$ = 0.10–1.00 MW in 0.10 MW steps; H = 2, 3, 4 h; $E_{ESS}$ = $P_{ESS}$ × H.	210 positive-capacity plans plus one explicit no-build plan, giving 211 alternatives.
33-bus retained comparison plans	Proposed ESS: bus 24, 1.00 MW/ 2.00 MWh (2 h); Fixed ESS: bus 33, 0.40 MW/ 0.80 MWh (2 h).	Both plans use the same fixed topology, lower-level model, scenarios, and economic settings.
240-bus ESS planning domain	16 pre-specified candidate locations; 2 h and 4 h durations; No ESS, Proposed/reference, load-center, and feeder-head strategies.	The 240-bus study is a separate engineering-scale case study rather than a replacement for the 33-bus design search.
Voltage-security criteria	0.95 p.u. for ADN-retrofitting/basic-feasibility assessment; 0.97 p.u. for stricter ESS-planning assessment; 0.96 p.u. included in threshold sensitivity.	Penalty-independent metrics include $V_{min}$, UVH, maximum undervoltage depth, UVE, and voltage margin; cases with absolute physical residuals > 10^-3^are infeasible.
TOU tariff	0.32, 0.68, 1.15, and 1.35 CNY/kWh for valley, flat, peak, and critical-peak periods, respectively.	Valley: h 1–8; Peak: h 11–12 and 15–19; Critical peak: h 20–21; remaining hours are flat.
Carbon parameters	Carbon price = 100 CNY/tCO_2_; grid emission factor = 0.6093 tCO_2_/MWh; MT emission factor = 0.645 tCO_2_/MWh.	Used in lower-level cost accounting where the carbon term is reported.
ESS economics	Power component = 433.68 CNY/kW and energy component = 826.98 CNY/kWh, derived from 2025 CNESA 2 h/4 h LFP EPC averages [40]; discount rate = 8%; project life = 15 years; fixed O&M = 33.036484 CNY/kW-year, benchmarked to PNNL [41].	CAPEX is converted to an equivalent daily value through the CRF; cost, lifetime, and discount-rate sensitivities are reported separately.
ESS efficiency, SOC, and degradation	Round-trip efficiency = 85%; cyclic 24 h SOC convention for the main representative-day model; DoD-dependent PWL cycle-degradation model; DoD sensitivity = 60%, 70%, 80%.	The separate 7-day interday check tests chronological SOC coupling; detailed PWL degradation data are reported outside this compact parameter table.
Historical scenario reduction	Each day is a 72-D joint vector (24 load + 24 PV + 24 wind). The 33-bus K = 3, 5, 10, and 20 sets use frozen input-risk features, seasonal top-decile risk pools, and annual input-side maxima; weights equal assigned historical-day counts/ 366.	K = 10 is the principal 33-bus setting; K20 is the higher-fidelity scenario-set check. Exact retention of the full-history worst-voltage day is audited separately for K3/K5/K10/K20.
Risk treatment	CVaR confidence levels α = 0.95, 0.96, and 0.97 are examined; voltage-violation and load-shedding penalties remain feasibility backstops.	Economic conclusions are interpreted together with penalty-independent physical security metrics.
Solver and numerical settings	Windows 11; MATLAB R2024b/ YALMIP/ Gurobi 13.0.2; target MIP gap ≤ 1% and time limit = 900 s where applied.	Post-solution feasibility used an absolute physical-residual tolerance of 10^-3^; larger residuals were classified as infeasible.


$$\begin{array}{c} UVH=\sum_{k=\text{1}}^{K}\pi_{k}\sum_{t=\text{1}}^{T}e_{(t,k)\Delta t},D_{max}=\underset{t,k}{\text{max}}d_{t,k} \end{array}$$

$$\begin{array}{c} UVE=\sum_{k=\text{1}}^{K}\pi_{k}\sum_{t=\text{1}}^{T}d_{(t,k)\Delta t} \end{array}$$ (44)

Finally, the mean voltage margin is calculated from the positive hourly margin of the probability-weighted feeder minimum:


$$\begin{array}{c} MVM=\frac{\text{1}}{T}\sum_{t=\text{1}}^{T}\text{max}\left(\text{0},{\bar{V}}_{min,t}-V_{lim}\right) \end{array}$$ (45)

The time step is one hour. Thus, undervoltage duration and exposure are explicitly probability-weighted across scenarios, maximum depth is taken across all hours and scenarios, and mean voltage margin is the average positive margin above the evaluation threshold.

The retained investment and operating-cost equations define a bilevel planning-operation evaluation in which each discrete ESS plan is assessed by solving the fixed-topology lower-level MISOCP under the representative scenarios. The final planning method is exhaustive evaluation of the complete discrete domain using daily-equivalent TOTEX.

PSO, GA, and DE are not the final planning method. They are evaluated only as search benchmarks against the known exhaustive-grid optimum under a common candidate-evaluation budget. The lower-level candidate evaluation is a fixed-topology MISOCP with DistFlow-SOCP network physics; its auxiliary binary variables enforce the exact PWL DoD degradation representation rather than feeder reconfiguration.

For reproducibility, PSO, GA, and DE each used a population of 6 and 30 independent runs with common seeds 2026072801–2026072830. Each run stopped after 60 unique valid candidate evaluations or 1,200 total proposals. Duplicate proposals did not consume the unique-evaluation budget. PSO used inertia 0.72 and cognitive/social coefficients 1.49/1.49; GA used 0.90 crossover, 0.10 mutation probability per dimension, binary tournament selection, and elitist replacement; DE used rand/1/bin mutation with differential weight 0.70 and crossover probability 0.90. All methods queried the same precomputed GRID211 objective surface; no solver call was made during this search benchmark.

## 3. Results

The lower-level MISOCP was implemented in MATLAB R2024b using YALMIP and solved with Gurobi 13.0.2 under Windows 11. A target MIP gap of 1% and a 900 s time limit were applied where applicable. The 33-bus principal study uses K10 historical scenarios and the complete GRID211 domain. K20 checks the selected design at higher scenario fidelity. The released 240-bus feeder provides a separate engineering-scale case study.

### 3.1. Uncertainty characterization and scenario reduction

Fig 6 shows the Year A trajectories and the input-risk-stratified K10 representatives. Fig 7 and Table 4 quantify the trade-off among fidelity, tail retention, and runtime. K20 gives the smallest maximum TOTEX error (1.40%) and UVE error (11.13%), whereas K10 provides the principal computational compromise. K3 does not retain the exact full-history worst-Vmin day; K5, K10, and K20 do. Although K10 does not minimize every fidelity error, it retains the selected design while substantially reducing computational burden; K20 and full-history robustness check are therefore used as complementary fidelity checks.

**Table 4:** Scenario-reduction fidelity and computational performance for K = 3, 5, 10, and 20.

K	Max TOTEX error (%)	Max UVE error (%)	Max |$V_{min}$ error| (p.u.)	Worst historical day retained	Runtime (s)
3	15.60	64.73	0.02052	No	**187.8**
5	47.65	149.85	**7.12 × 10^−5^**	Yes	448.4
10	12.49	42.69	6.66 × 10^−4^	Yes	911.7
20	**1.40**	**11.13**	6.35 × 10^−4^	Yes	2771.3

Note: Fidelity is assessed against the full-history reference. “Full-history worst-$V_{\text{min}}$ day retained exactly” indicates whether the exact Year A date producing the full-history minimum $V_{\text{min}}$ (2016-01-21) is included as a representative day. This categorical check does not by itself establish tail-distribution fidelity. Lower errors and runtime are preferable.

**Fig 6:**
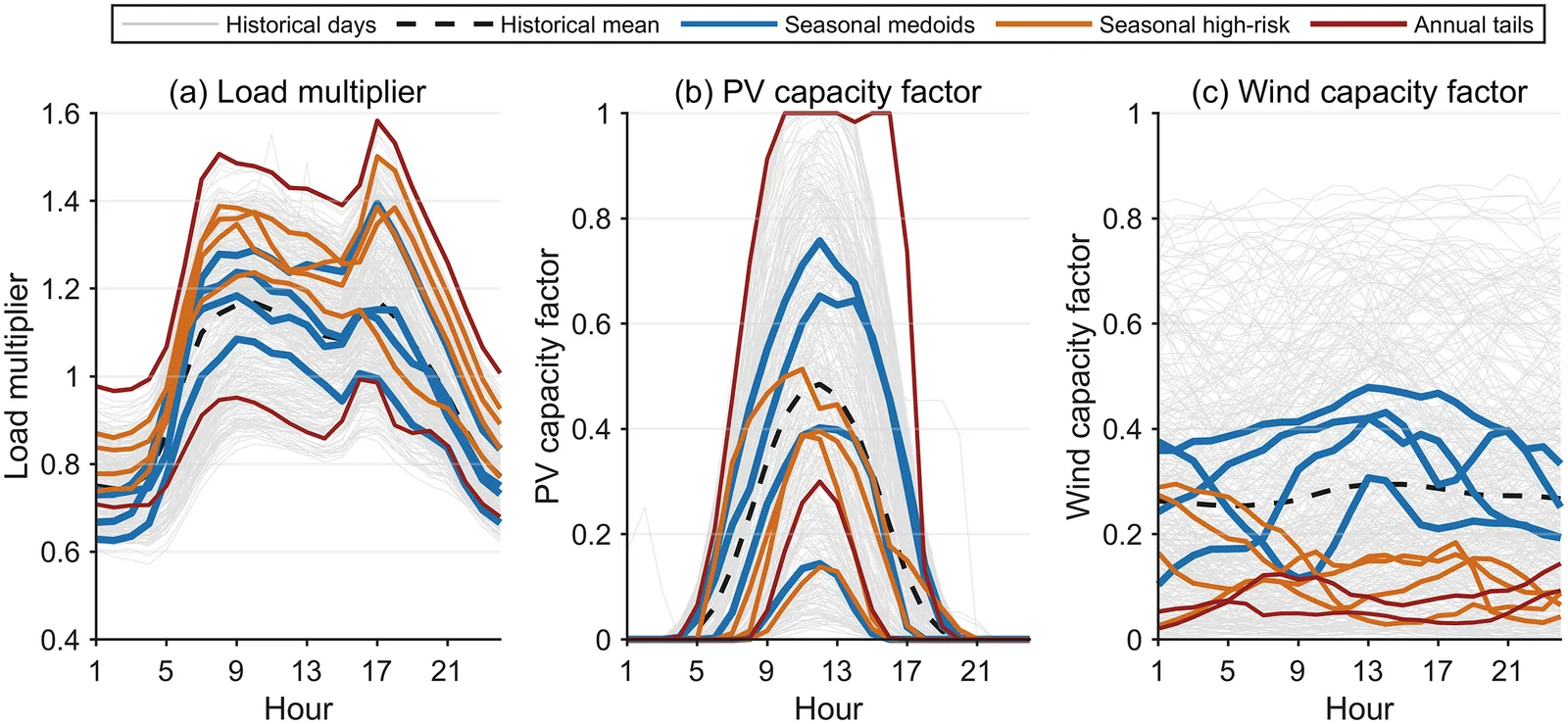
Historical Year A daily trajectories and the input-risk-stratified K = 10 representative set for load, photovoltaic, and wind profiles.

**Fig 7:**
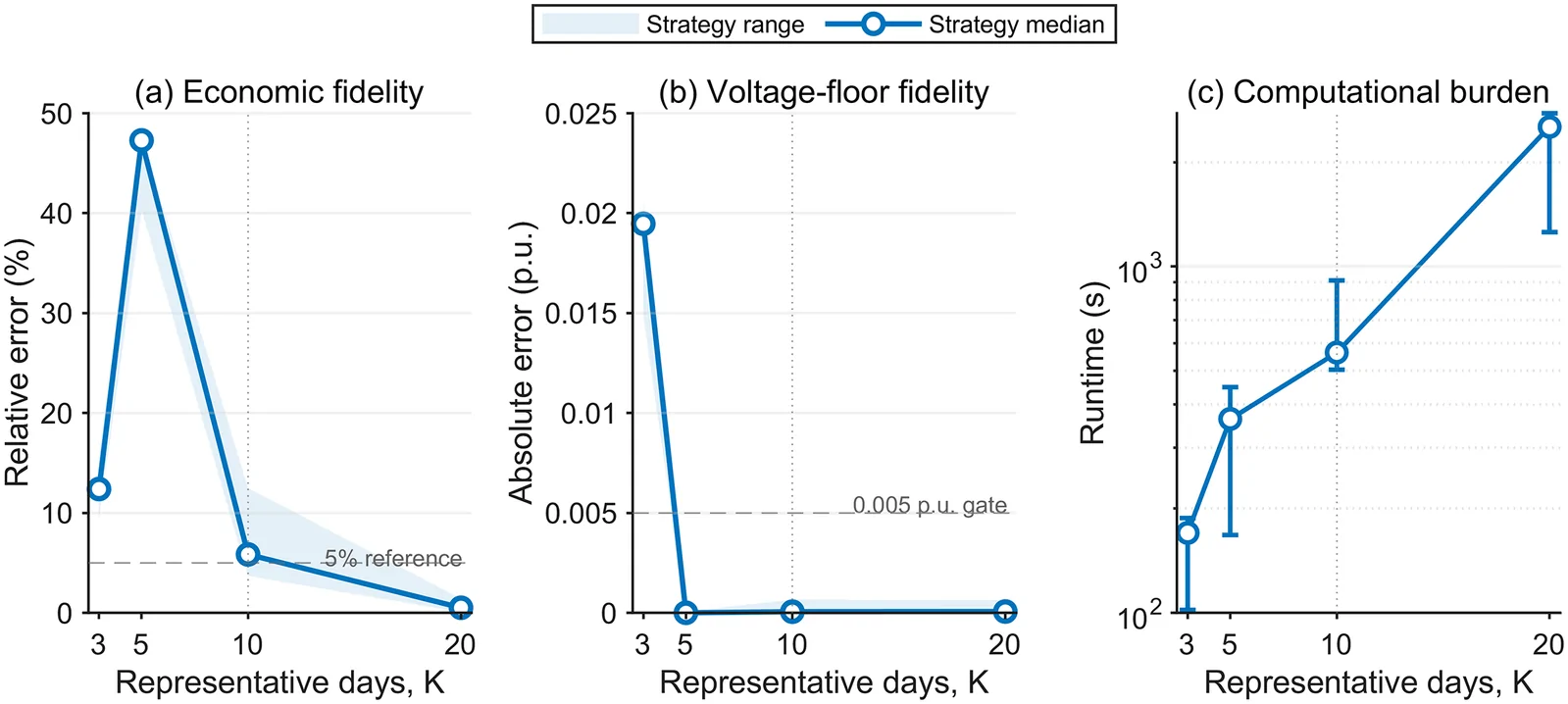
Scenario-count fidelity and computational trade-off for K = 3, 5, 10, and 20 representative days.

The selected ESS design is unchanged between K10 and K20: bus 24, 1.00 MW, 2.00 MWh, and 2 h. Tables 5 and 6 report the corresponding economic and voltage-security outcomes. K20 raises TOTEX from 51,826.24 to 52,900.74 CNY/day and retains the same minimum-voltage floor, while its longer violation duration and larger cumulative exposure provide a more conservative security assessment.

**Table 5:** Economic outcomes of the selected ESS designs for K = 10 and K = 20.

K	Selected bus	$P_{ESS}$ (MW)	$E_{ESS}$ (MWh)	Annualized ESS cost (CNY/day)	OPEX (CNY/day)	TOTEX (CNY/day)	Runtime (s)
10	24	1.00	2.00	**758.72**	**51067.52**	**51826.24**	**1286.8**
20	24	1.00	2.00	**758.72**	52142.02	52900.74	3379.3

Note: K = 10 is the principal reduced-scenario setting, whereas K = 20 provides a higher-fidelity scenario-set check. The K = 3 and K = 5 cases are used for scenario-reduction sensitivity assessment in Table 4. Boldface indicates the better directly comparable economic or computational value; tied values are both shown in bold.

**Table 6:** Voltage-security outcomes of the selected ESS designs for K = 10 and K = 20.

K	$V_{min}$ (p.u.)	UVH (h)	$D_{max}$ (p.u.)	UVE (p.u.·h)	MVM (p.u.)	Load shedding (MWh)
10	**0.95405**	**0.249**	**0.01595**	**0.001740**	**0.008688**	0.004297
20	**0.95405**	0.577	**0.01595**	0.002475	0.008464	**0.004082**

Note: UVH denotes undervoltage hours; $D_{\text{max}}$ is the maximum undervoltage depth; UVE is cumulative undervoltage exposure; and MVM is the mean voltage margin. Higher $V_{\text{min}}$ and MVM indicate better undervoltage-security performance, whereas lower UVH, $D_{\text{max}}$, UVE, and load shedding are preferable. The K = 3 and K = 5 cases are assessed as scenario-reduction sensitivity cases in Table 4. Boldface indicates the best directly comparable value; ties are both shown in bold.

### 3.2. ADN retrofitting benefits without ESS

Base A is a diagnostic resource-disabled stress case; Base B enables the active-network resources defined in the Materials and methods section while keeping the same topology. Fig 8 and Tables 7 and 8 show that Base A has a minimum voltage of 0.91452 p.u., 24 undervoltage hours, and 39.387412 MWh of load shedding. Base B reaches the 0.95 p.u. feasibility threshold without undervoltage exposure or shedding. The very large Base A cost is penalty-dominated and is not interpreted as a routine economic baseline.

**Table 7:** Voltage-security and operational metrics for ADN ablation.

Case	$V_{min}$ (p.u.)	UVH (h)	$D_{max}$ (p.u.)	UVE (p.u.·h)	Load shedding (MWh)	Network loss (MWh)
Base A (ADN off)	0.91452	24.000	0.03548	0.707862	39.387412	3.550742
Base B (ADN on)	**0.95000**	**0.000**	**0**	**0**	**0**	**1.565515**

Note: Base A is the resource-disabled diagnostic stress case; Base B is the active-ADN baseline. Boldface indicates the best directly comparable physical value(s).

**Table 8:** Economic cost breakdown for ADN ablation.

Case	Grid cost	MT cost	Carbon cost	Shedding penalty	Voltage penalty	Total operating cost
Base A (ADN off)	41703.52	0.00	3358.98	3938741.20	856955.54	4840759.23
Base B (ADN on)	14600.55	27909.94	3870.58	0.00	0.00	46381.07

Note: All monetary quantities are reported on the same daily CNY basis. OPEX is the sum of the grid-purchase cost, MT cost, carbon-emission cost, load-shedding penalty, and voltage-violation penalty reported in the table. Physical voltage-risk indicators are reported separately in Table 7. Boldface indicates the lowest directly comparable cost value(s).

**Fig 8:**
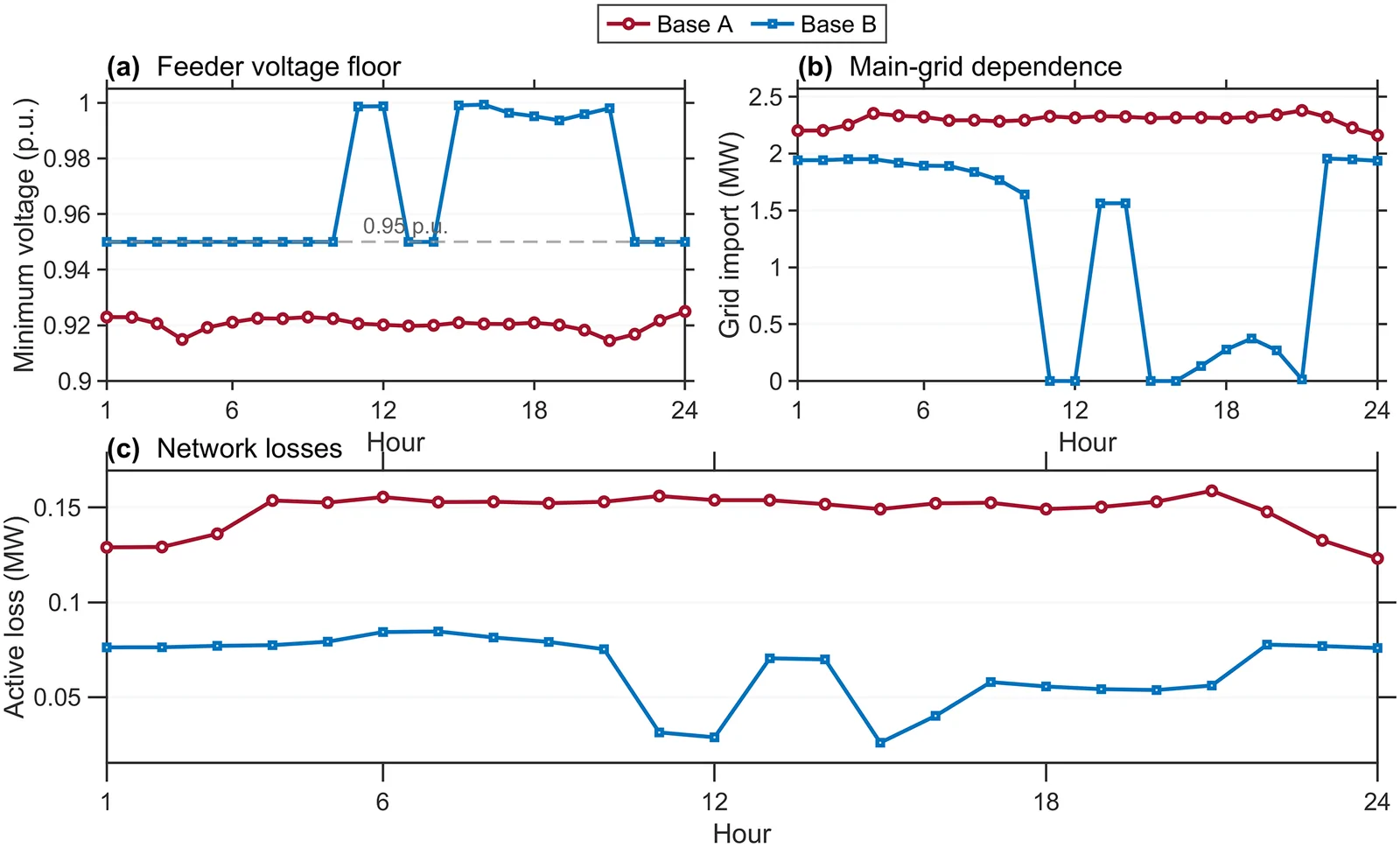
ADN ablation comparison of feeder voltage floor, main-grid dependence, and network losses for Base A and Base B.

### 3.3. Uncertainty-aware assessment of ESS value

Figs 9 and 10 compare No ESS, Fixed ESS, and Proposed ESS under deterministic and K10 evaluations. Under deterministic evaluation, the proposed design reduces TOTEX from 49,542.30 to 46,966.82 CNY/day. Table 9 shows that, under K10, the reduction is from 73,151.02 to 51,826.24 CNY/day. Table 10 also shows a higher minimum voltage and lower UVE for Proposed ESS. The uncertainty-aware benefit therefore reflects reduced exposure to stressed historical conditions rather than price arbitrage alone.

**Table 9:** Economic comparison of the three planning strategies under deterministic and K10 uncertainty-aware evaluations.

Strategy	Annualized ESS cost (CNY/day)	Deterministic evaluation		K10 uncertainty-aware evaluation	
		OPEX (CNY/day)	TOTEX (CNY/day)	OPEX (CNY/day)	TOTEX (CNY/day)
No ESS	0.00	49542.30	49542.30	73151.02	73151.02
Fixed ESS	303.49	48453.73	48757.22	58679.94	58983.43
Proposed ESS	758.72	**46208.10**	**46966.82**	**51067.52**	**51826.24**

Note: K10 denotes the uncertainty-aware evaluation using 10 representative scenarios and their associated weights. Detailed K10 cost components are reported in Table 12; this table is limited to the cross-protocol economic comparison to avoid duplicating the component-level breakdown. Boldface indicates the best directly comparable economic performance.

**Table 10:** Voltage-security comparison of the three planning strategies under deterministic and K10 uncertainty-aware evaluations.

Strategy	Deterministic evaluation			K10 uncertainty-aware evaluation		
	$V_{min}$ (p.u.)	UVH (h)	UVE (p.u.·h)	$V_{min}$ (p.u.)	UVH (h)	UVE (p.u.·h)
No ESS	**0.97000**	**0.000**	**0**	0.94138	1.582	0.013528
Fixed ESS	**0.97000**	**0.000**	3.67 × 10^-11^	0.94081	1.224	0.007272
Proposed ESS	**0.97000**	**0.000**	**0**	**0.95405**	**0.249**	**0.001740**

Note: $V_{\text{min}}$, UVH, and UVE summarize the voltage floor, violation duration, and cumulative undervoltage exposure, respectively. The same physical metric definitions are used for the deterministic and K10 evaluations. Higher $V_{\text{min}}$ and lower UVH/UVE are preferable; tied best values are all shown in bold.

**Fig 9:**
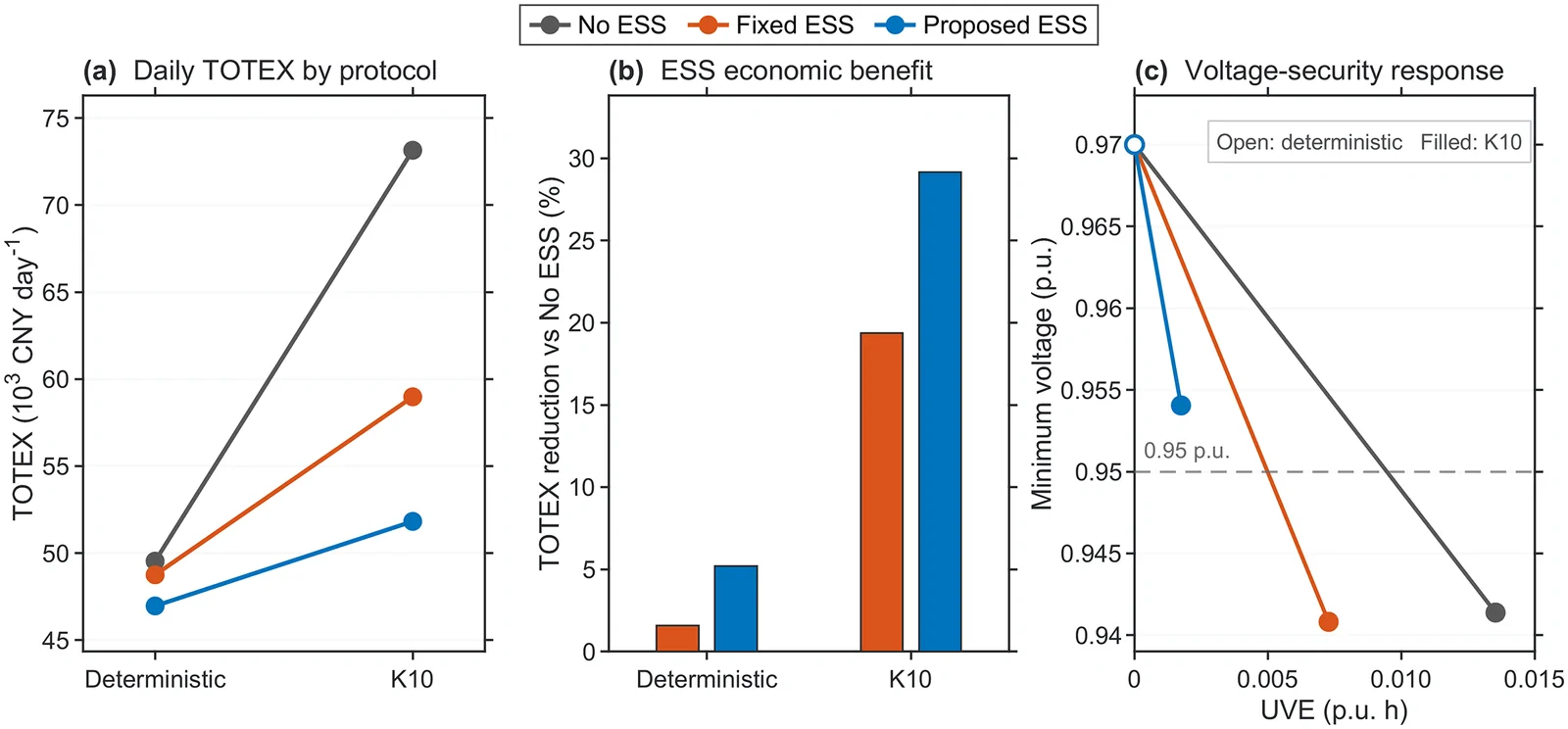
ESS ablation under deterministic and input-risk-stratified K10 evaluation.

**Fig 10:**
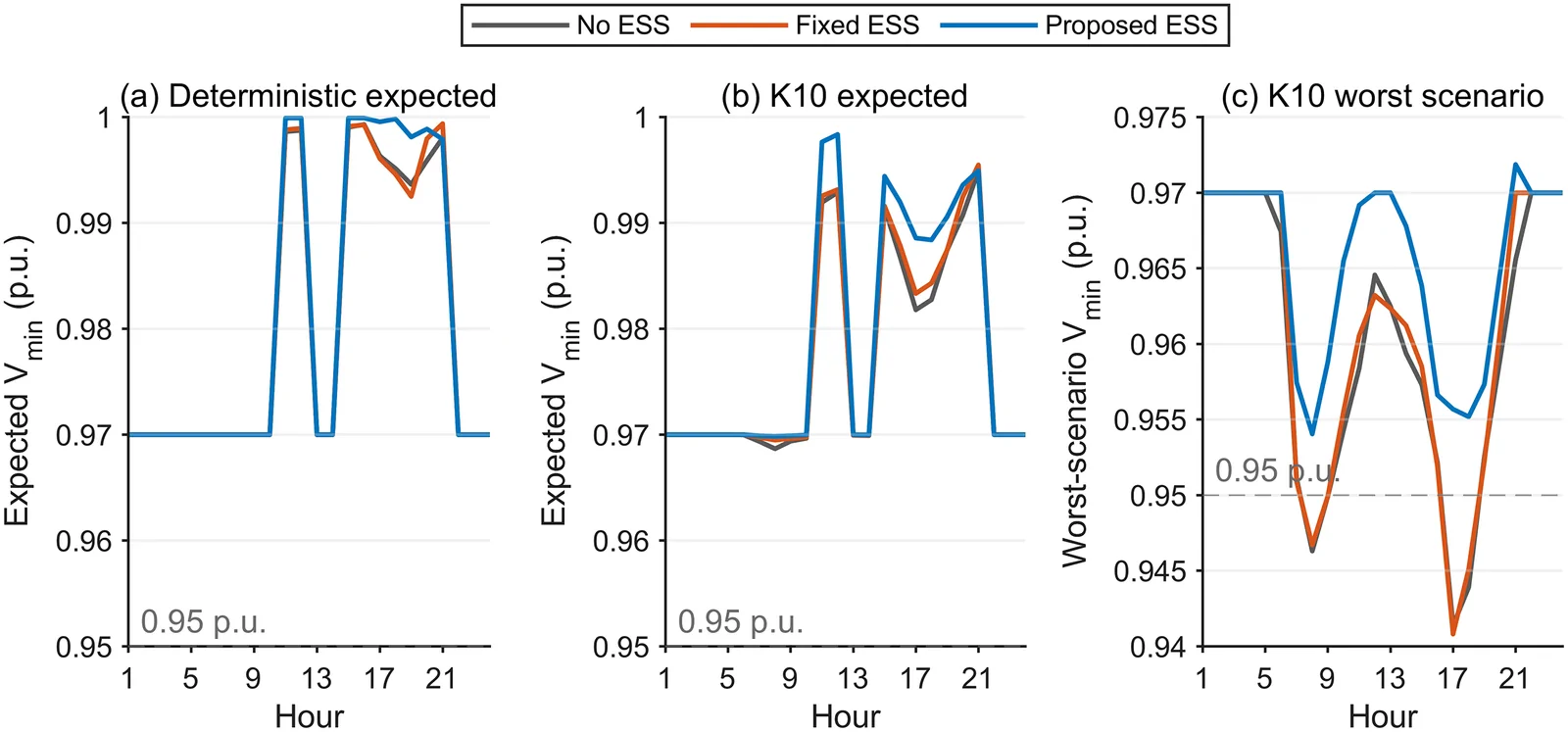
Hourly voltage-security evidence under deterministic and K10 evaluations for No ESS, Fixed ESS, and Proposed ESS.

### 3.4. Bilevel ESS planning comparison under uncertainty

#### 3.4.1. Complete discrete-domain planning result.

Fig 11 reports the complete GRID211 landscape. The economic optimum is bus 24 with 1.00 MW/ 2.00 MWh and 2 h duration; the leading alternatives are listed in Table 11. Exhaustive enumeration, rather than a metaheuristic output, is the authoritative planning result. Under K10, Proposed ESS gives the lowest TOTEX among the three strategies and reduces the security-penalty component from 20,761.21 to 1,584.94 CNY/day.

**Table 11:** Top-ranked ESS alternatives from the complete 33-bus discrete-domain evaluation.

Rank	ESS bus	$P_{ESS}$ (MW)	$E_{ESS}$ (MWh)	Duration (h)	TOTEX (CNY/day)
**1**	**24**	**1.00**	**2.00**	**2**	**51826.24**
2	24	1.00	4.00	4	51887.63
3	25	1.00	3.00	3	51888.42
4	25	1.00	2.00	2	51905.65
5	30	1.00	2.00	2	51955.55
6	30	1.00	3.00	3	51957.42
7	24	1.00	3.00	3	51993.17
8	25	1.00	4.00	4	52009.24
9	30	1.00	4.00	4	52072.60
10	24	0.90	2.70	3	52179.36

Note: Only the top-ranked alternatives are summarized in the main text; the complete 211-alternative grid is provided separately in the accompanying data package. The first-ranked design is shown in bold.

**Fig 11:**
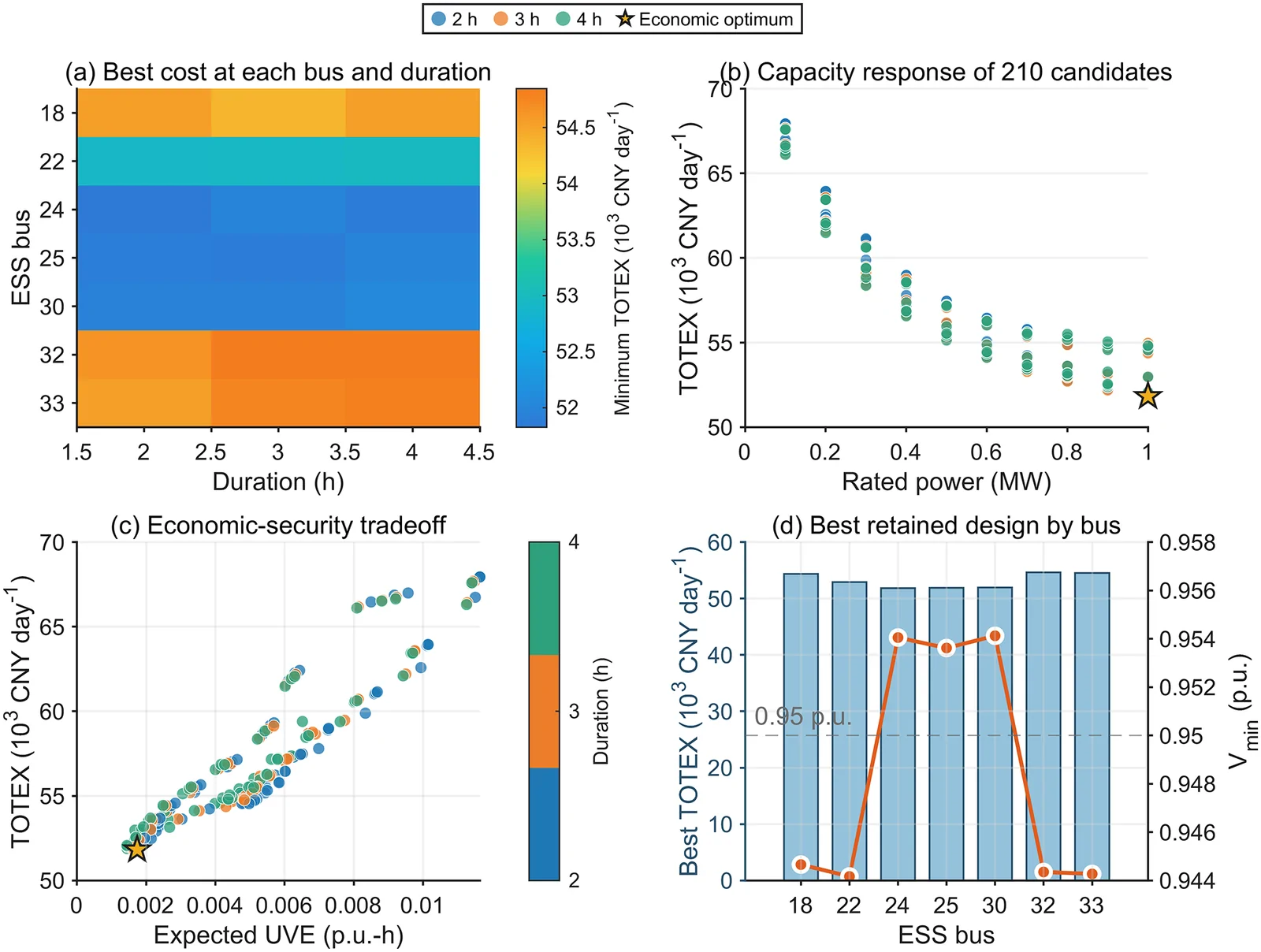
Complete GRID211 siting-sizing-duration landscape and the exhaustive-grid economic optimum.

#### 3.4.2. TOTEX comparison and economic interpretation.

Fig 12A,B and Table 12 separate non-security operating cost, security penalties, annualized ESS cost, and TOTEX. Proposed ESS has the lowest total expenditure even after its larger investment component is included.

**Table 12:** TOTEX and cost breakdown for the three 33-bus planning strategies.

Strategy	Non-security operating cost (CNY/day)	Security penalties	Annualized ESS cost	TOTEX
No ESS	52389.81	20761.21	0.00	73151.02
Fixed ESS	51501.84	7178.09	303.49	58983.43
Proposed ESS	**49482.58**	**1584.94**	758.72	**51826.24**

Note: Non-security operating cost includes grid-purchase, MT operating, ESS degradation, and carbon-emission costs, whereas security penalties comprise voltage-violation and load-shedding penalties. Their sum equals OPEX as defined in Equations (1),(2), and TOTEX further includes the annualized ESS cost.

**Fig 12:**
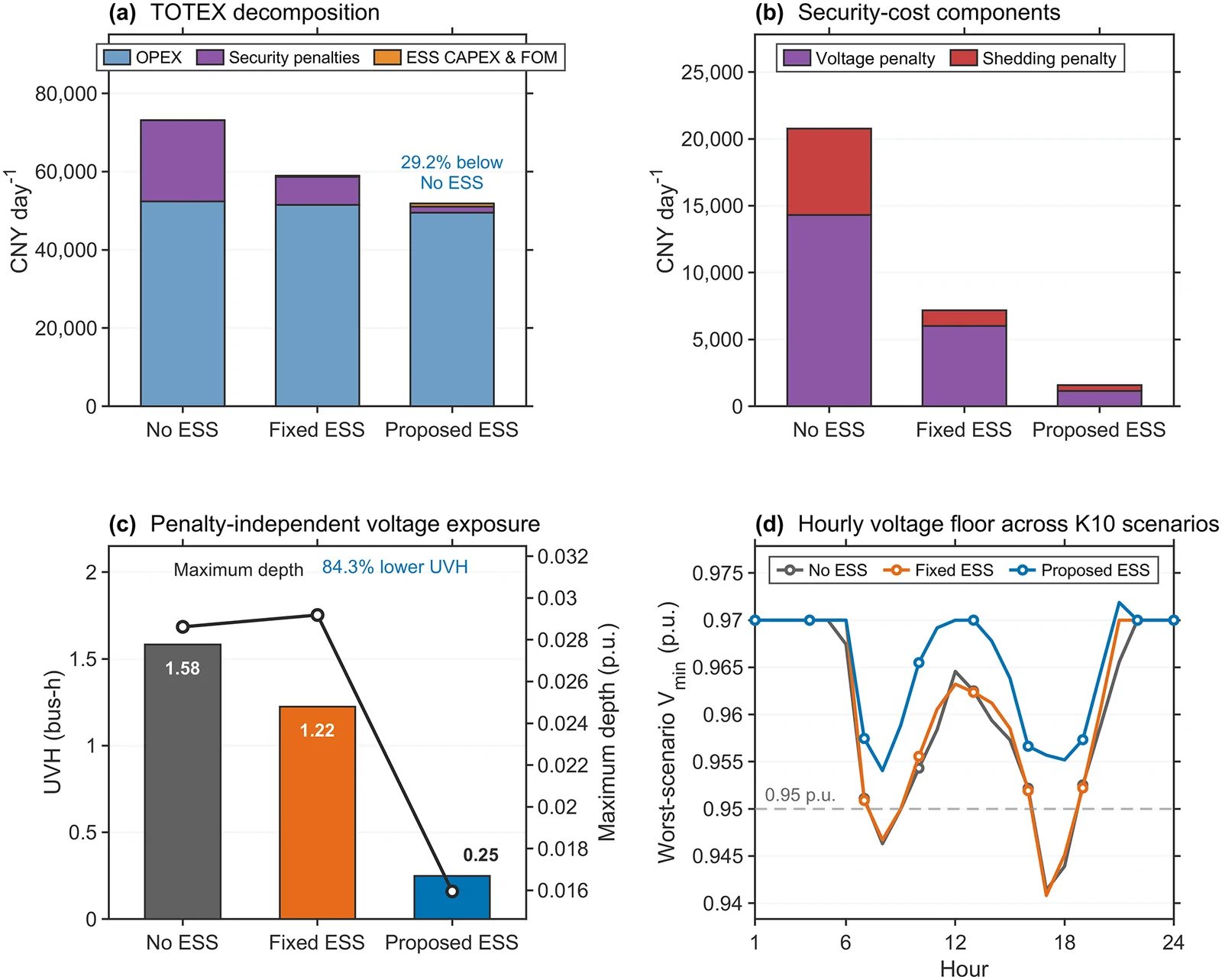
K10 cost and voltage-security decomposition for No ESS, Fixed ESS, and Proposed ESS.

#### 3.4.3. Penalty-independent voltage-security trade-off.

Fig 12C,D and Table 13 show that Proposed ESS raises the minimum voltage to 0.95405 p.u. and reduces cumulative UVE to 0.001740 p.u.·h. These physical metrics confirm that the ranking is not created solely by the monetary penalty coefficients.

**Table 13:** Penalty-independent voltage-security metrics for the three 33-bus planning strategies.

Strategy	$V_{min}$ (p.u.)	UVH (h)	$D_{max}$ (p.u.)	UVE (p.u.·h)	MVM (p.u.)	Load shedding (MWh)
No ESS	0.94138	1.582	0.02862	0.013528	0.007112	0.064617
Fixed ESS	0.94081	1.224	0.02919	0.007272	0.007428	0.011744
Proposed ESS	**0.95405**	**0.249**	**0.01595**	**0.001740**	**0.008688**	**0.004297**

Note: Higher $V_{\text{min}}$ and MVM indicate better voltage security, whereas lower UVH, $D_{\text{max}}$, UVE, and load shedding are preferable. Boldface indicates the best value(s).

#### 3.4.4. Operational mechanism and tail-risk compression.

Figs 13 and 14 trace the grid exchange, power balance, ESS schedule, state of charge, and local-resource coordination that produce the observed cost-security trade-off. Fig 15 shows that the advantage extends across the empirical Year A daily-cost distribution and compresses the upper tail rather than relying only on one average day.

**Fig 13:**
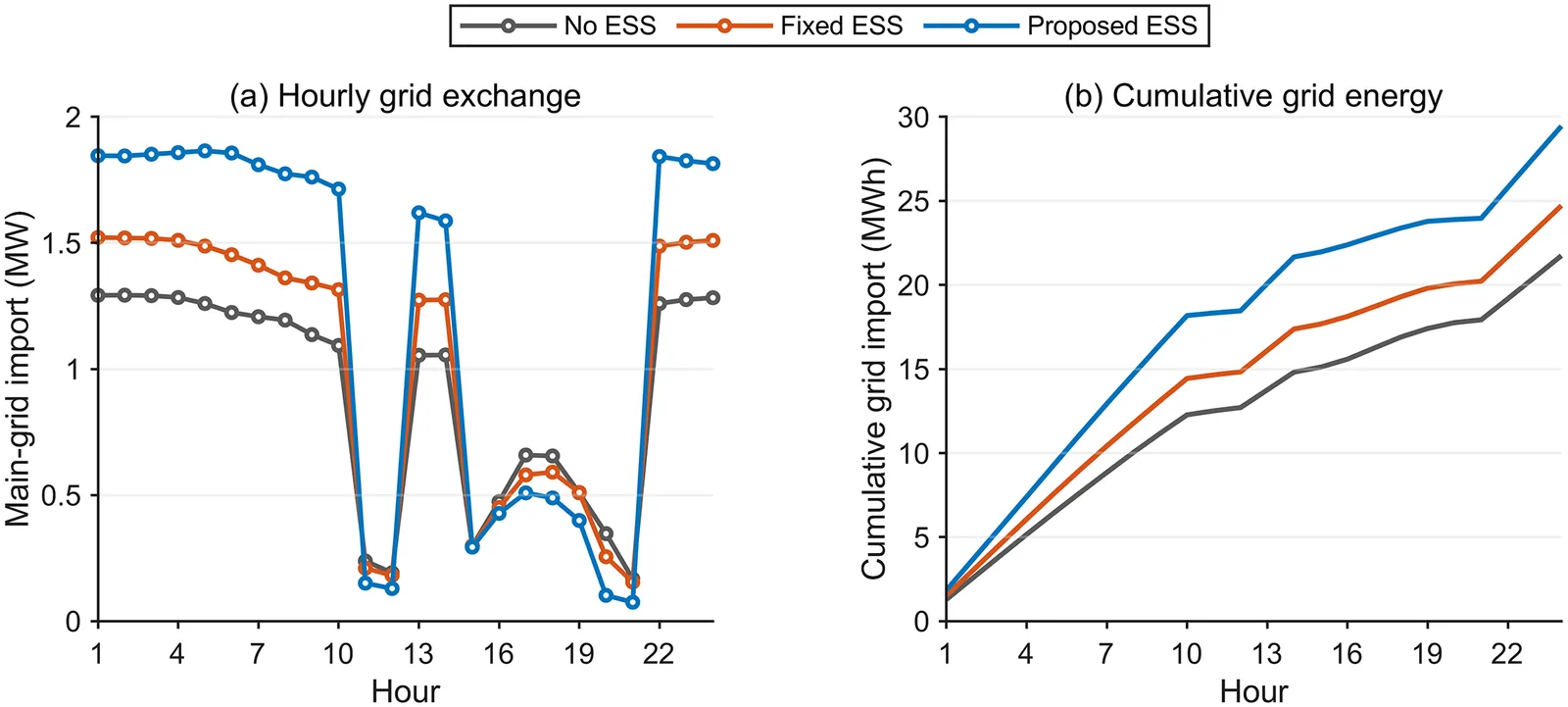
Main-grid exchange under the K10 representative scenarios.

**Fig 14:**
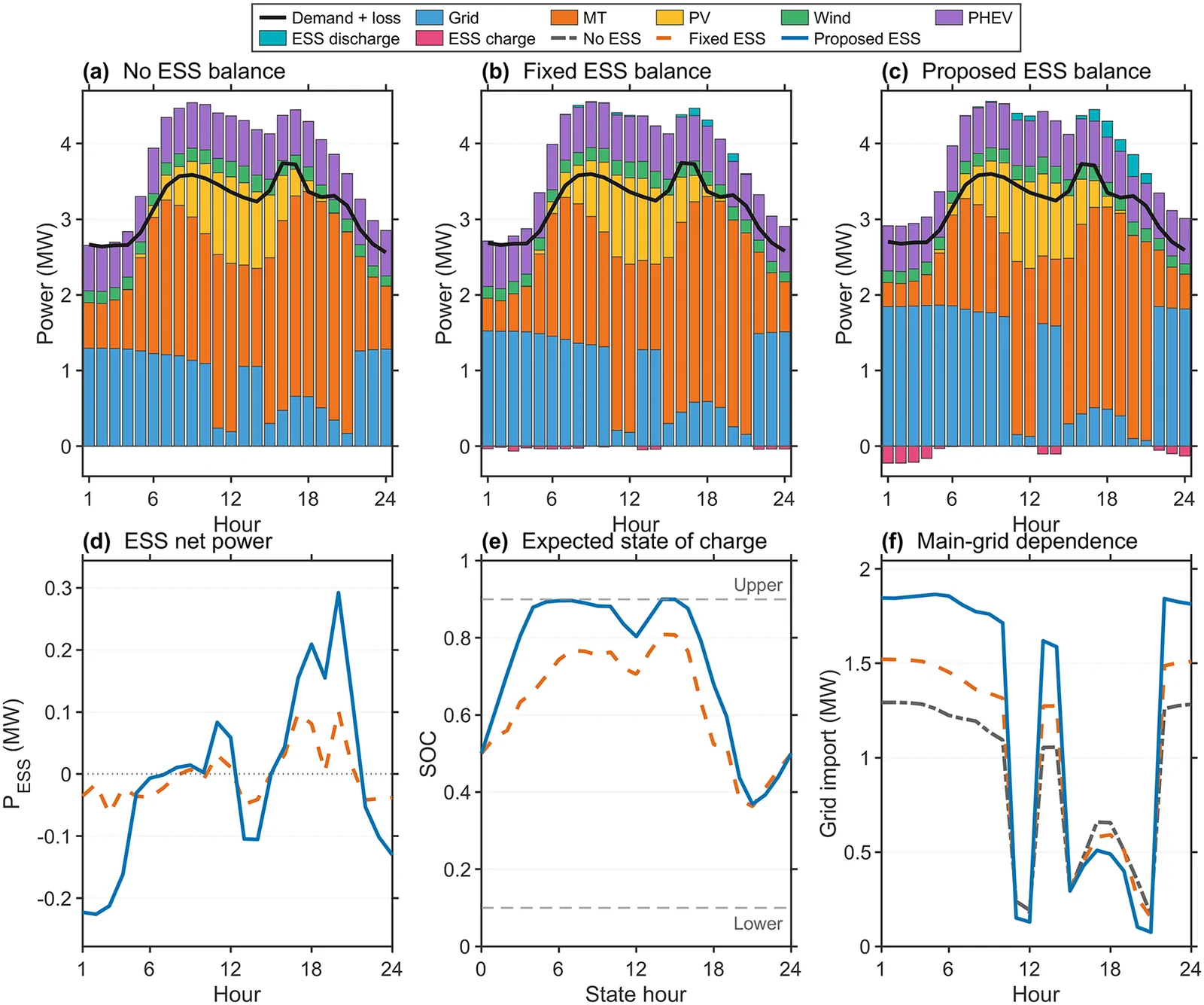
Hourly power balance, ESS operation, state of charge, and main-grid dependence under the K10 comparison.

**Fig 15:**
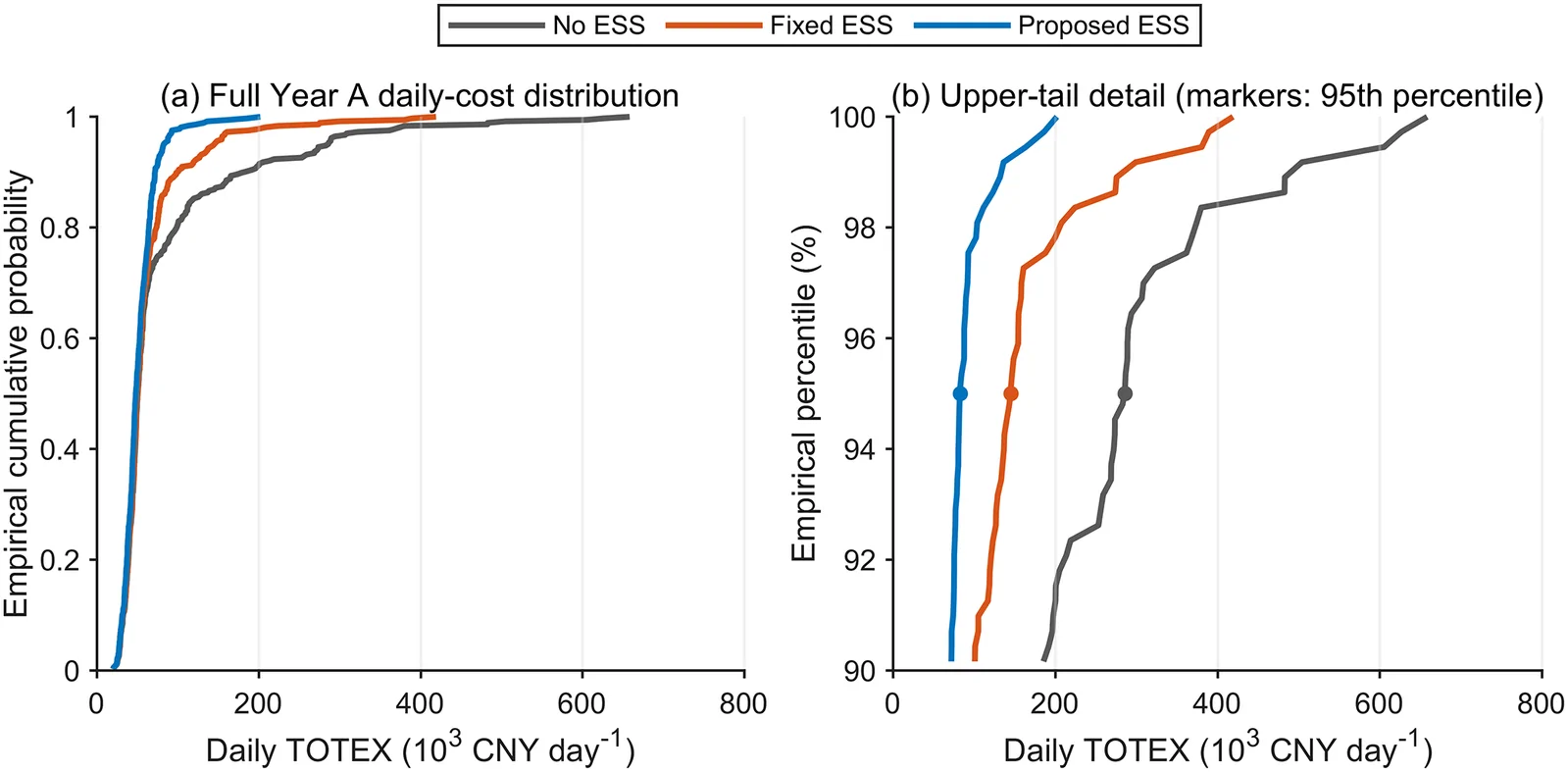
Empirical distribution of Year A daily operating outcomes.

### 3.5. Upper-level algorithm benchmarking: PSO vs GA vs DE

PSO, GA, and DE were evaluated under a common candidate-evaluation budget using the completed discrete objective surface [42,43]. S1 Table reports their ability to recover the known grid optimum and top-ranked region. The benchmark assesses search efficiency only and does not redefine the planning solution. PSO recovered the grid-best design in 86.67% of runs, compared with 80.00% for GA and 43.33% for DE.

### 3.6. Multi-day and cross-year robustness checks

Fig 16 and Table 14 compare the three strategies across 366 Year A days and 364 complete Year B days. Proposed ESS gives mean daily TOTEX values of 51.91 and 51.56 thousand CNY/day, respectively, and the lowest mean shedding in both years. Figs 17 and 18 provide a separate seven-day chronological test with interday state-of-charge coupling and compare it with daily resetting. These tests support robustness beyond the representative-day calculation without changing the principal planning domain.

**Table 14:** Annual and cross-year robustness results for the three 33-bus planning strategies.

Year	Strategy	Days	Mean daily TOTEX (10³ CNY/day)	Worst $V_{min}$ (p.u.)	Maximum daily UVE (p.u.·h)	Mean shed energy (MWh/day)
Year A	No ESS	366	83.59	0.94138	0.234243	0.113811
Year A	Fixed ESS	366	62.64	0.94087	0.220522	0.008686
Year A	Proposed ESS	366	**51.91**	**0.95395**	**0.117952**	**0.002318**
Year B	No ESS	364	81.70	0.94213	0.222593	0.101495
Year B	Fixed ESS	364	62.05	0.94350	0.211371	0.004570
Year B	Proposed ESS	364	**51.56**	**0.95369**	**0.117159**	**0.000686**

Note: This table reports the principal cross-year economic and voltage-security indicators; detailed annual distributions are shown in the corresponding figure and accompanying data. Within each year, boldface indicates lower TOTEX/UVE/shedding and higher $V_{\text{min}}$.

**Fig 16:**
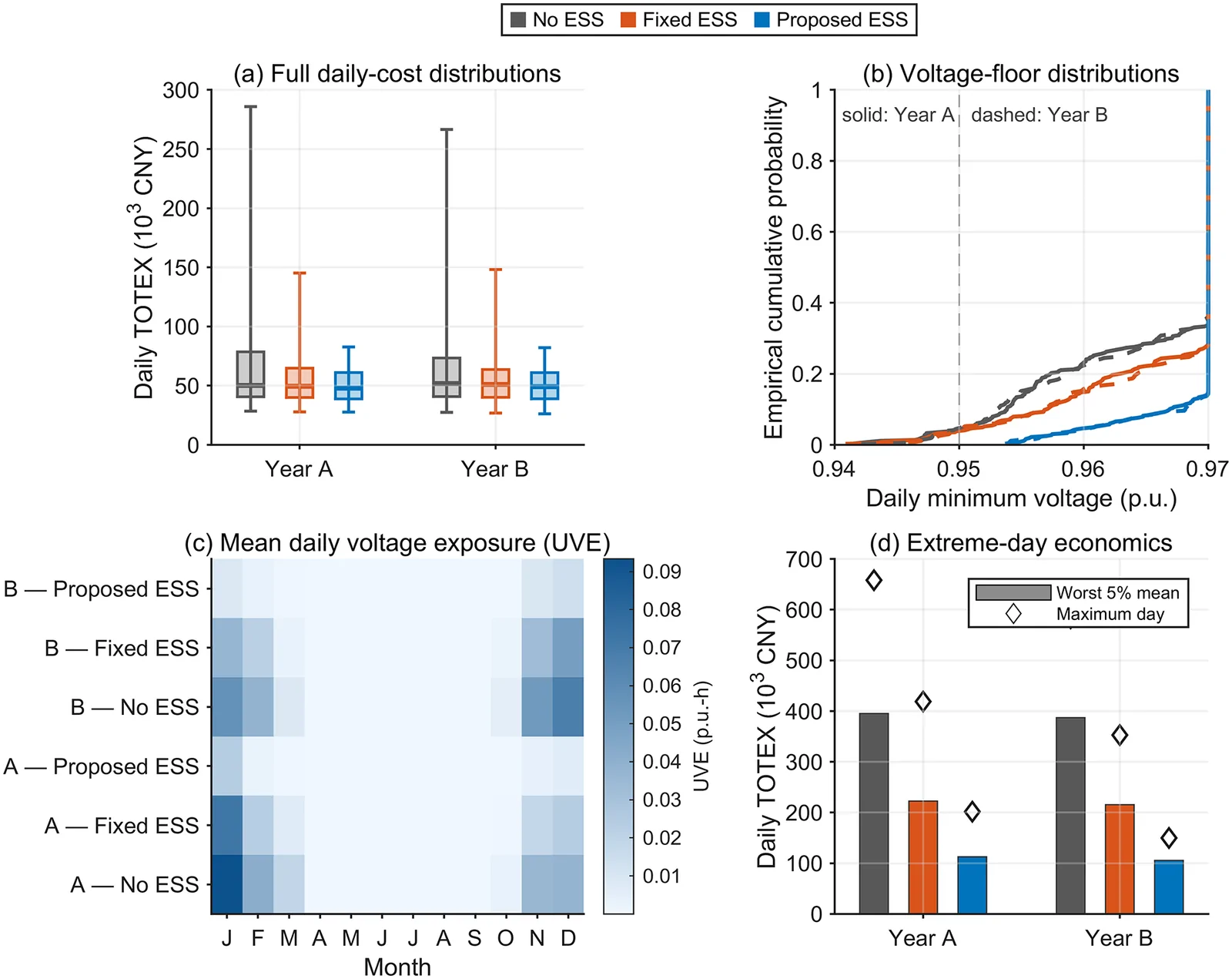
Annual and cross-year robustness of No ESS, Fixed ESS, and Proposed ESS.

**Fig 17:**
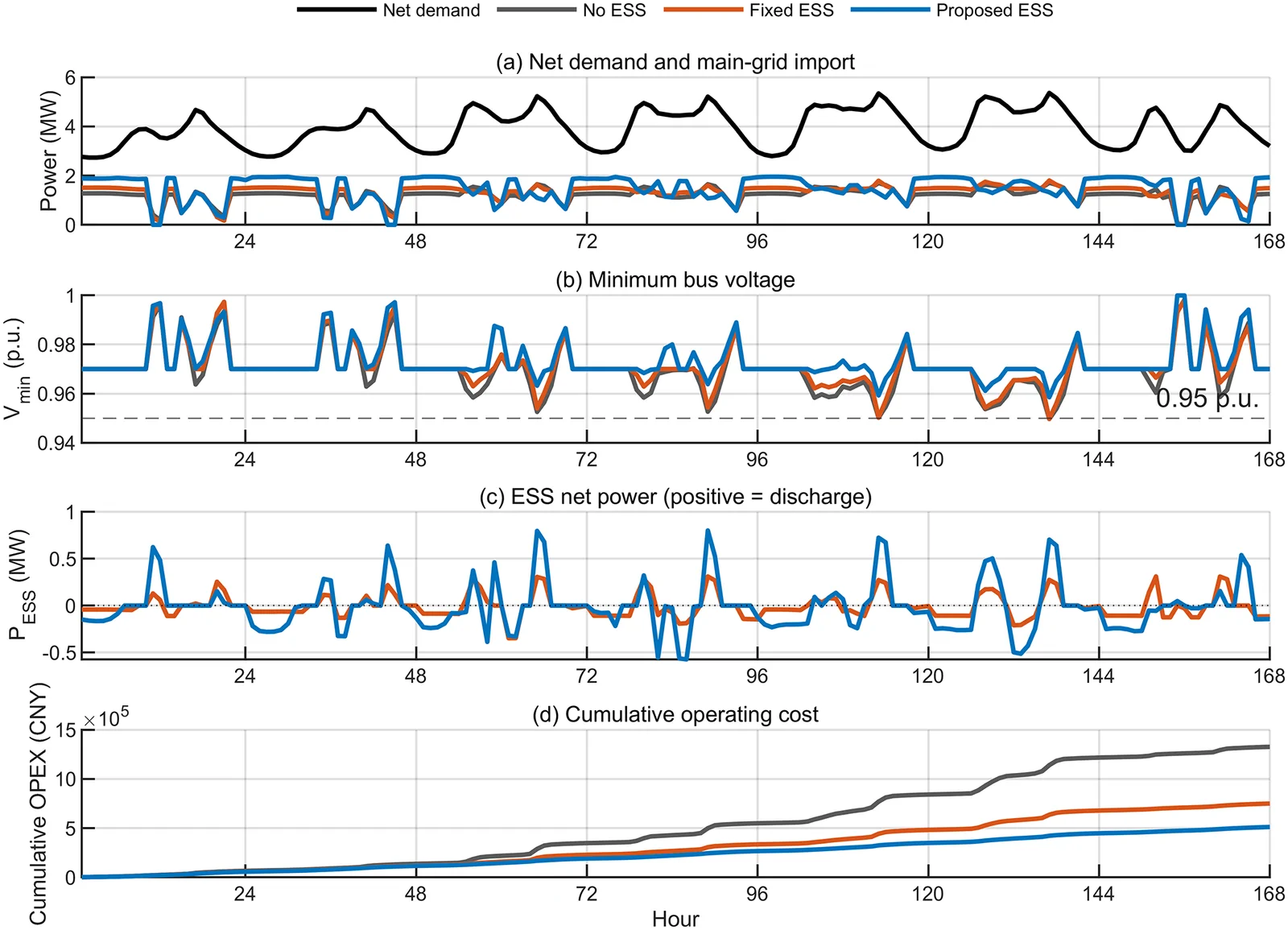
Seven-day chronological operation under interday SOC coupling.

**Fig 18:**
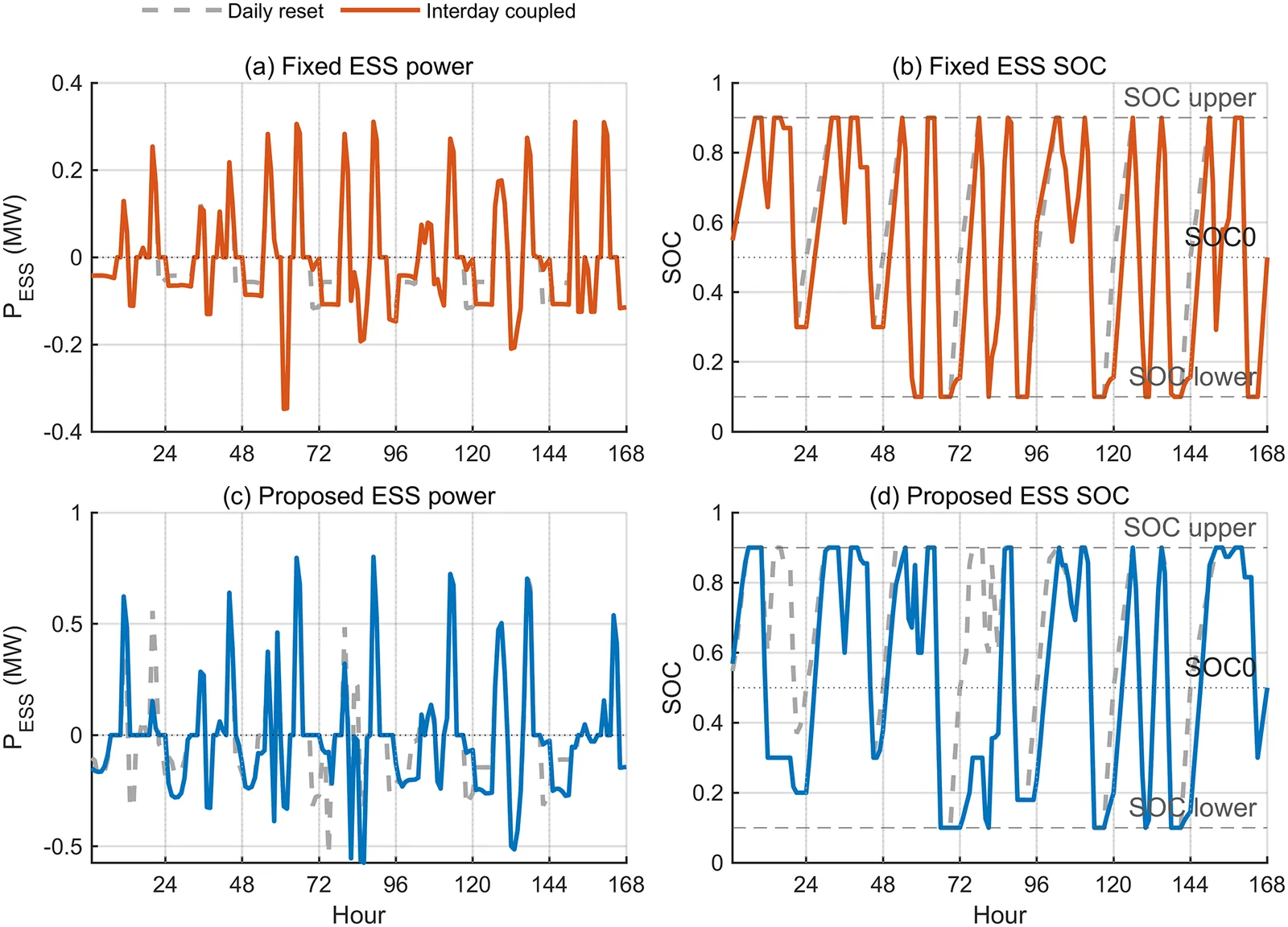
Effect of daily SOC resetting and interday SOC coupling on Fixed ESS and Proposed ESS.

### 3.7. Engineering-scale case study on the released 240-bus feeder

The released 240-bus screen contains 154 positive-capacity designs across 16 retained candidate buses plus one no-build alternative (155 total). The 2 h family comprises 16 locations at seven power levels (112 designs), and the predefined 4 h subset comprises six locations at the same seven power levels (42 designs). Fig 19 reports this candidate landscape. Fig 20 compares the reference design with the load-center and feeder-head heuristics. In the normal location comparison, the reference design is bus3129; bus3077 and bus2002 represent the load-center and feeder-head heuristics. Tables 15 and 16 show that the reference design improves voltage exposure, branch loading, overload, and loss relative to No ESS and the heuristic locations. Its TOTEX is 65.56 CNY/day above No ESS, so the result is interpreted as security improvement at a small incremental economic cost rather than as an economic saving.

**Table 15:** Economic comparison of representative ESS strategies on the released 240-bus feeder.

Strategy	Bus	$P_{ESS}$ (MW)	$E_{ESS}$ (MWh)	Grid + MT operating cost (CNY/day)	Annualized ESS cost (CNY/day)	TOTEX (CNY/day)
No ESS	—	0.00	0.00	169593.35	0.00	**182454.68**
Proposed ESS (reference)	bus3129	0.25	0.50	**169096.70**	520.58	182520.24
Load-center ESS	bus3077	0.25	0.50	169113.10	520.58	182537.37
Feeder-head ESS	bus2002	0.25	0.50	169179.64	520.58	182607.49

Note: Grid + MT operating cost comprises only grid-purchase and MT operating costs. TOTEX additionally includes applicable carbon-emission, ESS degradation, voltage-violation, and load-shedding terms, together with the annualized ESS investment cost shown in the table. To keep the main table compact, these additional modeled terms are included in TOTEX but are not reported as separate columns. Boldface marks the minimum grid + MT operating cost and minimum TOTEX across the reported strategies; annualized ESS investment cost is a design component and is not ranked independently.

**Table 16:** Voltage-security and operational comparison of representative ESS strategies on the released 240-bus feeder.

Strategy	$V_{min}$ (p.u.)	UVH/ voltage exposure	UVE (p.u.·h)	Max branch loading (p.u.)	Overload duration/ energy	Network loss (MWh)
No ESS	0.96696	1.250 h	0.002784	1.514192	5.500 h/ 0.913418 p.u.·h	3.472267
Proposed ESS (reference)	**0.96776**	**1.125 h**	**0.000915**	**1.501820**	**5.000 h/ 0.857481 p.u.·h**	**3.401085**
Load-center ESS	**0.96776**	1.250 h	0.001816	1.501985	5.000 h/ 0.858265 p.u.·h	3.411623
Feeder-head ESS	0.96696	1.250 h	0.002801	1.514192	5.375 h/ 0.914904 p.u.·h	3.465704

Note: Only physical and operational metrics are reported here; monetary quantities are reported separately in Table 15. Higher $V_{\text{min}}$ is preferable; lower voltage exposure, branch loading, overload, and network loss are preferable. Boldface indicates the best value(s).

**Fig 19:**
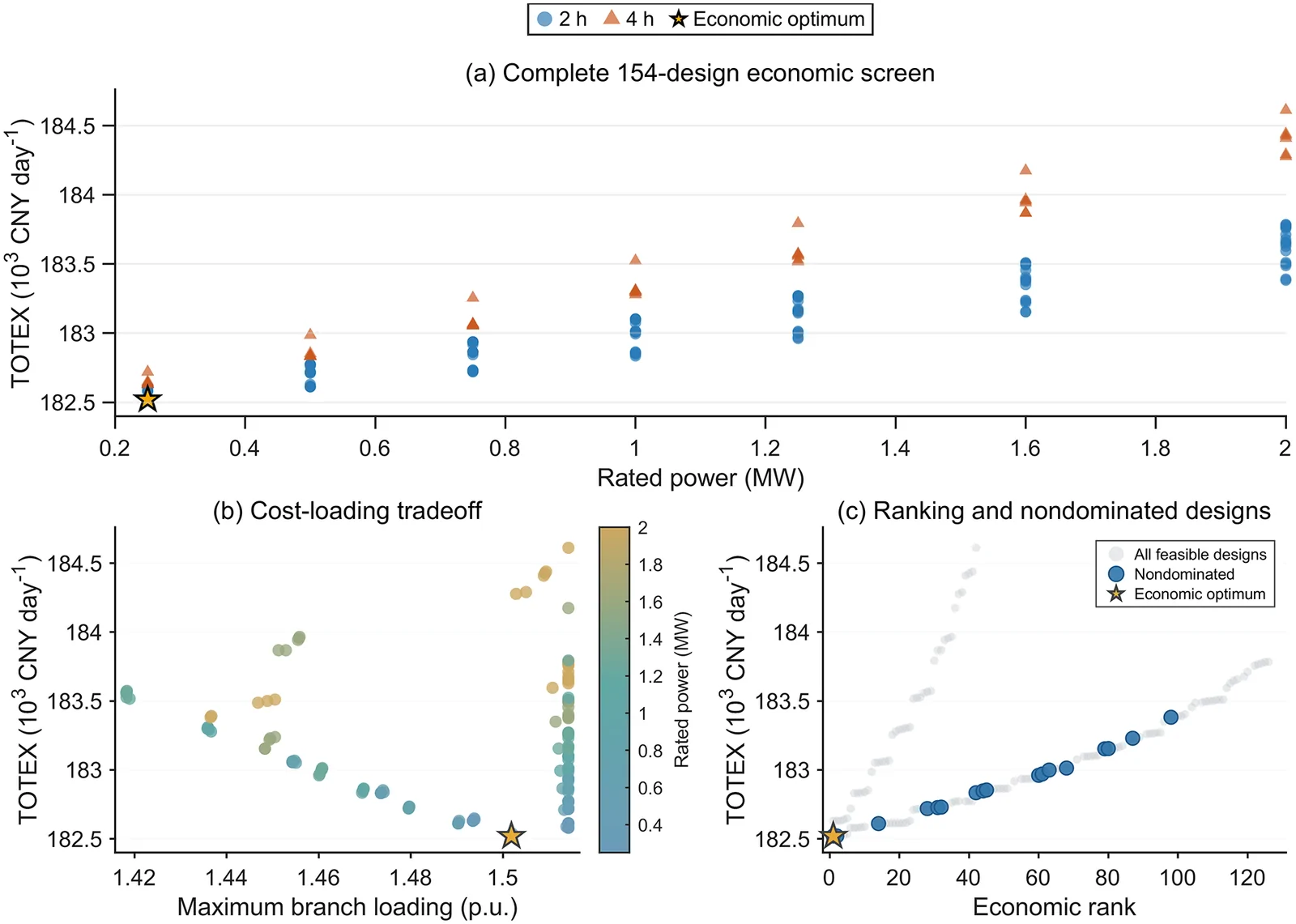
Final released 240-node ESS candidate screen: 154 positive-capacity designs across 16 candidate buses; the no-build alternative is reported separately in Table 3, giving 155 total alternatives.

**Fig 20:**
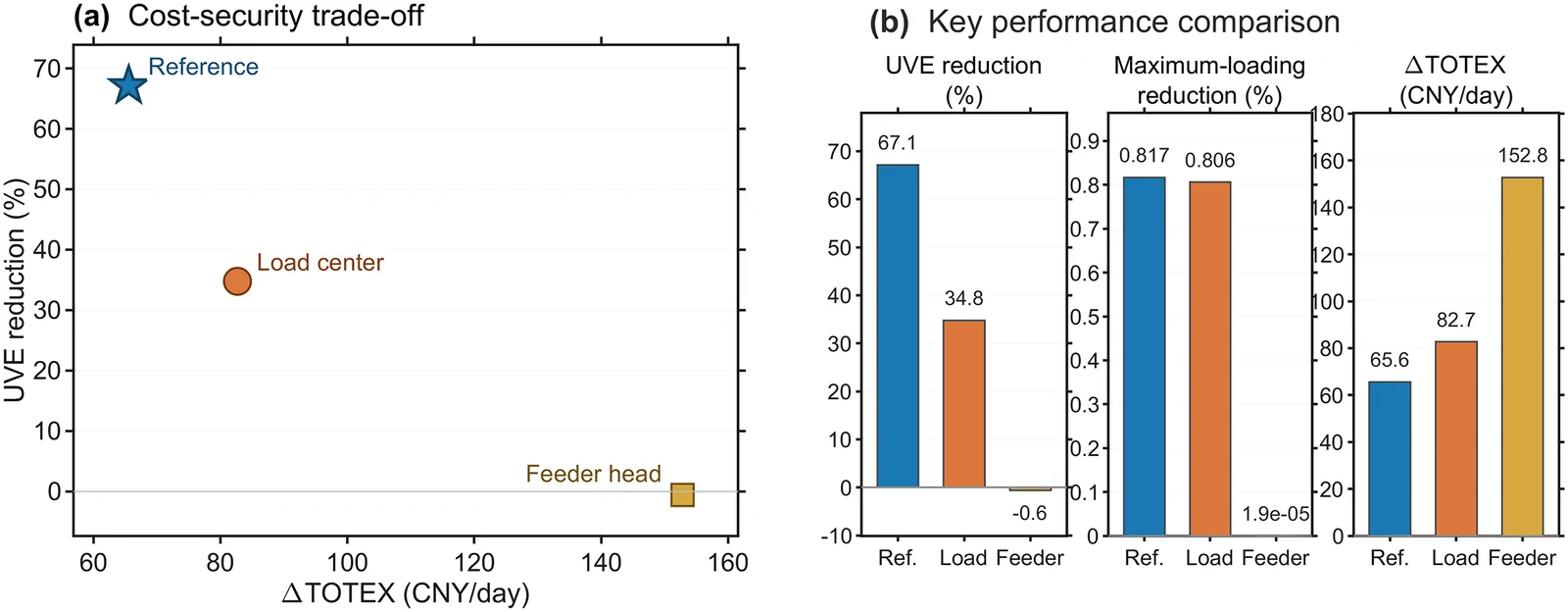
Reference-versus-fixed-location comparison on the released 240-node feeder using Reference, load-center, and feeder-head locations.

Fig 21 is a separate K8 severe-stress test. Its Fixed ESS comparator is bus1017 and is not the load-center or feeder-head case in Tables 15,16. This stress test evaluates the matched-capacity economy-security pattern, while Table 17 reports numerical consistency and computational performance.

**Table 17:** Numerical consistency and computational performance of the 33-bus and released 240-bus systems.

System	Metric	Reported value
33-bus	Maximum physical residual	9.73 × 10^-9^
33-bus	Selected-design K10 evaluation runtime	1286.78^-s^
240-bus	Model size (rows/ columns/ nonzeros)	139000/ 259848/ 693648
240-bus	Second-order-cone/ quadratic constraints	83136
240-bus	Per-case K8 evaluation runtime	14.04–31.10^-s^
240-bus	Maximum SOCP-to-AC voltage mismatch	2.13 × 10^-8^ p.u.
240-bus	Maximum physical residual	9.99 × 10^-7^
240-bus	AC-audit scenario-hour points	192 per strategy; 768 total

Note: Runtimes are solver wall-clock values for complete multi-scenario case evaluations: one selected-design K10 evaluation for the 33-bus system and the reported K8 no-ESS/reference/fixed cases for the 240-bus system. The 192-per-strategy and 768-total counts are scenario-hour operating points used in the AC back-substitution audit, not separate optimization runs; neither runtime is the elapsed time for the full candidate screen. Absolute physical residuals below 10^−3^ were treated as numerically acceptable.

**Fig 21:**
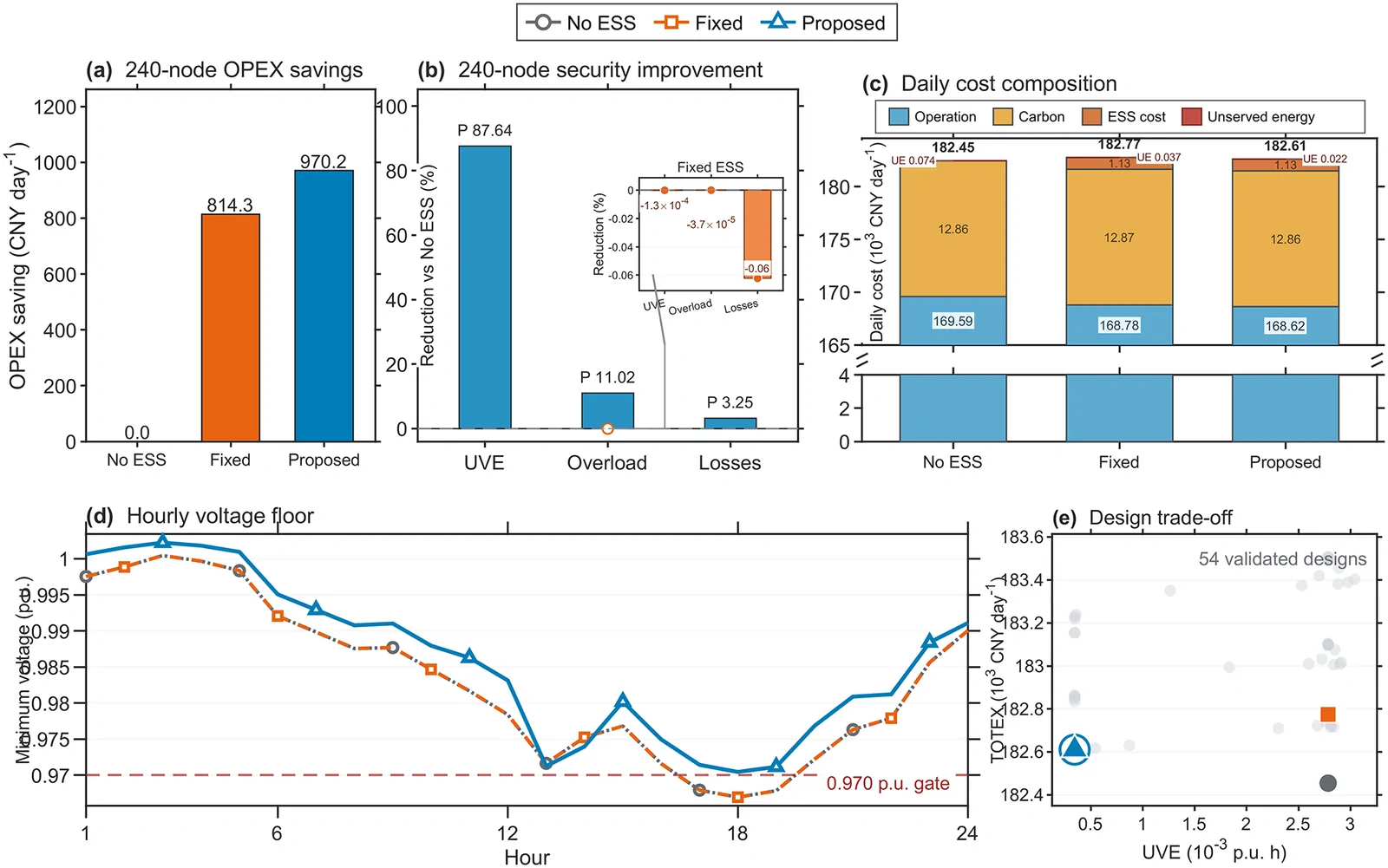
Severe-stress test of No ESS, Fixed ESS, and Proposed ESS on the released 240-node feeder. The Fixed ESS comparator corresponds to bus1017 in the matched-capacity fixed-location set used for this stress-test analysis.

### 3.8. Sensitivity and robustness analysis

Fig 22 consolidates the economic, degradation, depth-of-discharge, duration, risk-preference, voltage-threshold, and representative-scenario analyses. Tables 18 and 19 provide the exact values and definitions. The discount-rate panel re-ranks the discrete alternatives, whereas the battery-cost, lifetime, degradation, and DoD cases re-evaluate the retained proposed design. The scenario-count worst-Vmin error is a fidelity measure, not an ESS-performance ranking.

**Table 18:** Economic and storage-model sensitivity results.

Sensitivity factor	Level	TOTEX (CNY/day)	Design/ evaluation basis
**Battery-cost multiplier**	0.50	51492.13	Proposed ESS: Bus 24, 1.00 MW/ 2.00 MWh (H2)
	0.70	51625.78	Proposed ESS: Bus 24, 1.00 MW/ 2.00 MWh (H2)
	0.85	51726.01	Proposed ESS: Bus 24, 1.00 MW/ 2.00 MWh (H2)
	1.00	51826.24	Proposed ESS: Bus 24, 1.00 MW/ 2.00 MWh (H2)
	1.15	51926.47	Proposed ESS: Bus 24, 1.00 MW/ 2.00 MWh (H2)
**Project lifetime**	8 years	52153.31	Proposed ESS: Bus 24, 1.00 MW/ 2.00 MWh (H2)
	10 years	52010.41	Proposed ESS: Bus 24, 1.00 MW/ 2.00 MWh (H2)
	12 years	51916.98	Proposed ESS: Bus 24, 1.00 MW/ 2.00 MWh (H2)
	15 years	51826.24	Proposed ESS: Bus 24, 1.00 MW/ 2.00 MWh (H2)
**Discount rate**	4%	51611.99	Best design: Bus 24, 1.00 MW/ 4.00 MWh (H4)
	6%	51745.48	Best design: Bus 24, 1.00 MW/ 4.00 MWh (H4)
	8%	51826.24	Best design: Bus 24, 1.00 MW/ 2.00 MWh (H2)
	10%	51910.00	Best design: Bus 24, 1.00 MW/ 2.00 MWh (H2)
	12%	51997.80	Best design: Bus 24, 1.00 MW/ 2.00 MWh (H2)
**Degradation-cost multiplier**	0.50	51767.10	Proposed ESS (retained design)
	0.75	51827.34	Proposed ESS (retained design)
	1.00	51826.24	Proposed ESS (retained design)
	1.25	51881.89	Proposed ESS (retained design)
	1.50	51901.10	Proposed ESS (retained design)
**Allowable DoD**	60%	52070.93	Proposed ESS: Bus 24, 1.00 MW/ 2.00 MWh
	70%	51937.83	Proposed ESS: Bus 24, 1.00 MW/ 2.00 MWh
	80%	51826.24	Proposed ESS: Bus 24, 1.00 MW/ 2.00 MWh
**ESS duration class**	2 h	51826.24	Class minimum: Bus 24, 1.00 MW/ 2.00 MWh
	3 h	51888.42	Class minimum: Bus 25, 1.00 MW/ 3.00 MWh
	4 h	51887.63	Class minimum: Bus 24, 1.00 MW/ 4.00 MWh

Note: For the battery-cost, project-lifetime, degradation-cost, and DoD sensitivities, the baseline Proposed ESS design is retained and re-evaluated under each tested parameter level. For the discount-rate sensitivity, the reported “Best design” is the minimum-TOTEX design after re-ranking the candidate designs at each discount rate. For the ESS-duration sensitivity, “Class minimum” denotes the minimum-TOTEX design within each duration class.

**Table 19:** Risk, voltage-threshold, and representative-scenario sensitivity results.

Sensitivity factor	Level	Metric	No ESS	Fixed ESS	Proposed ESS
**CVaR confidence level**	0.95	CVaR	316358.21	184757.44	**107607.40**
	0.96		356745.30	209050.54	**116728.40**
	0.97		424057.12	249539.05	**131930.07**
**Voltage evaluation threshold**	0.95 p.u.	UVE (p.u.·h)	0.000233	0.000209	**0**
	0.96 p.u.		0.002775	0.001257	**0.000309**
	0.97 p.u.		0.013528	0.007272	**0.001740**
**Representative-scenario treatment**	K = 3	Worst-$V_{min}$ error (p.u.)	0.0148392	0.0194694	0.0205165
	K = 5		1.36 × 10^−9^	1.96 × 10^−7^	7.12 × 10^−5^
	K = 10		4.09 × 10^−8^	5.50 × 10^−5^	6.66 × 10^−4^
	K = 20		1.88 × 10^−8^	6.31 × 10^−5^	6.35 × 10^−4^

Note: Lower CVaR and UVE indicate better strategy performance; the best strategy value in each such row is shown in bold. Lower worst-$V_{\text{min}}$ error indicates better scenario-reduction fidelity and is not an ESS-performance ranking.

**Fig 22:**
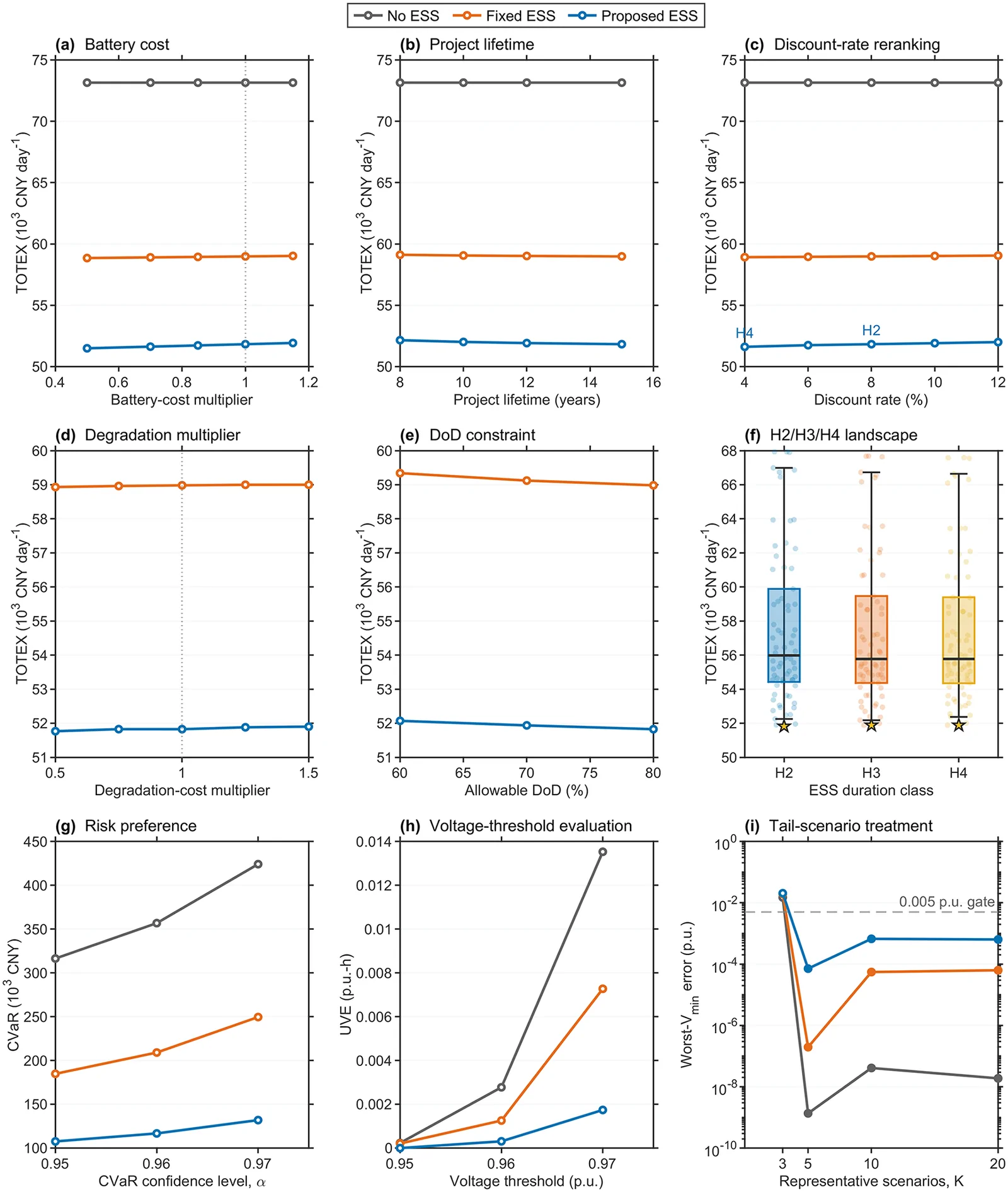
Combined economic, ESS-model, risk, voltage-threshold, and representative-scenario sensitivity analysis.

### 3.9. ESS-aware topology robustness check

The main planning comparison uses one common radial topology for No ESS, Fixed ESS, and Proposed ESS to isolate differences associated with the storage plans. To examine topology sensitivity after storage installation, we fixed the retained Proposed ESS at bus 24 (1.00 MW/ 2.00 MWh, 2 h) and repeated the same Stage-I topology-identification procedure used for the baseline reference network.

The baseline open-branch set is [23,29,37,39,44], whereas the ESS-aware set is [23,26,29,39,44]. Branch 26 changed from closed to open and branch 31 from open to closed, giving a Hamming distance of 2.

The ESS-aware topology was then fixed and evaluated using the same K10 operational model used in the main comparison. Relative to the selected Proposed ESS on the baseline topology, TOTEX increased from 51,826.24 to 53,762.99 CNY/day (+3.74%), while Vmin increased from 0.954051 to 0.955298 p.u., UVH decreased from 0.2486 to 0.1913 h, and UVE decreased from 0.001740 to 0.001516 p.u.·h. Load shedding increased from 0.004297 to 0.006224 MWh and network loss from 1.3973 to 1.7063 MWh. The result therefore indicates a mixed economy-security trade-off rather than uniform improvement as summarized in Table 20. Because the ESS design is held fixed, this sensitivity analysis does not constitute joint topology-storage planning or establish a globally optimal topology.

**Table 20:** ESS-aware topology sensitivity for the selected Proposed ESS.

Topology basis	Open branches	Changed branches	TOTEX (CNY/day)	Vmin (p.u.)	UVH (h)	UVE (p.u.·h)	Load shedding (MWh)	Network loss (MWh)
Baseline topology	[23,29,37,39,44]	None	51,826.24	0.954051	0.2486	0.001740	0.004297	1.3973
ESS-aware topology	[23,26,29,39,44]	26 open; 31 closed	53,762.99 (+3.74%)	0.955298	0.1913	0.001516	0.006224	1.7063

## 4. Discussion

### 4.1. Main methodological contribution and evidence boundary

The methodological contribution is not a new standalone optimizer or a new conic relaxation. It is an auditable attribution protocol that combines historical joint uncertainty, input-risk stratification with exact worst-day-retention auditing, fixed-topology attribution, discrete ESS siting-sizing-duration decisions, a fixed-topology MISOCP evaluator with DistFlow-SOCP network physics, daily-equivalent TOTEX, and penalty-independent voltage-security metrics. This structure separates storage effects from changes in scenario representation or feeder topology.

The complete 33-bus domain supports the planning result within the declared discrete design space, whereas the released 240-bus feeder supports executability and a cost-security trade-off at the tested engineering scale. The evidence does not establish a universal optimizer advantage, a universal storage size, field performance, or scalability to arbitrary network sizes.

### 4.2. How uncertainty changes the assessed ESS value

Historical uncertainty changes storage value because stressed load-renewable combinations contribute disproportionately to voltage exposure, load-shedding backstops, and security-related costs. The deterministic and K10 results therefore differ in more than average procurement cost. K20 selects the same bus, power, energy, and duration and retains the same minimum-voltage floor, but its longer violation duration and larger cumulative exposure show that a higher-fidelity representation can be more conservative without changing the selected plan. Cross-year and seven-day tests further separate this conclusion from one representative-day calculation.

### 4.3. Voltage-security interpretation beyond monetary penalties

Voltage-security claims are based on the joint interpretation of $V_{\text{min}}$, UVH, $D_{\text{max}}$, cumulative UVE, and MVM, not on monetary penalties alone. $V_{\text{min}}$ identifies the voltage floor; UVH and UVE describe violation duration and accumulated exposure; $D_{\text{max}}$ identifies the deepest excursion; and MVM summarizes the remaining margin. Voltage and shedding penalties keep rare stressed cases solvable but do not substitute for these physical indicators.

The results do not imply that ESS replaces an on-load tap changer, SVC, capacitor bank, or inverter-based reactive-power control. Those alternatives were not subjected to a common investment comparison. The narrower conclusion is that, within the stated ADN resource set and fixed topology, the retained ESS improves the reported economy-security trade-off.

### 4.4. Fixed topology, ESS duration, topology sensitivity, and temporal scope

A fixed topology is defensible when switching states are prescribed by protection, maintenance, regulatory, or operating policy, or when the scientific objective is attribution to storage on a common network. If topology is an active decision, joint reconfiguration and ESS planning could change the preferred location, economic value, and security improvement. The present study reports a focused topology-sensitivity check; joint switching-storage planning remains outside its scope.

The 33-bus design domain uses discrete 2, 3, and 4 h duration classes, while the 240-bus screen uses bounded 2 h and 4 h families. Relaxing this grid could widen the CAPEX-OPEX frontier: longer durations may favor multi-hour energy shifting, whereas shorter durations may remain attractive for local voltage or overload support. A continuous-duration optimum was not solved.

The principal planning model is a 24 h representative-day model with a cyclic terminal-SOC condition. Annual Year A and Year B evaluations test repeated daily economics and seasonal variation, and the seven-day test adds interday SOC coupling. These checks do not constitute full-year chronological co-optimization or seasonal energy-storage planning.

### 4.5. Scalability, generalizability, limitations, and future work

For the fixed-topology MISOCP evaluator with DistFlow-SOCP network physics, network-state variables and constraints grow approximately with branches × time periods × scenarios before presolve, with additional binary-variable growth from the exact PWL degradation segments. The tested 240-bus instance contained 139,000 rows, 259,848 columns, 693,648 nonzeros, and 83,136 second-order-cone/quadratic constraints. Reported per-case K8 multi-scenario evaluation runtimes were 14.04–31.10 s across the listed 240-bus cases; the maximum SOCP-to-AC voltage mismatch was 2.13 × 10^−8^ p.u., and the maximum physical residual was 9.99 × 10^−7^. Accordingly, computational burden can increase with the number of scenarios, ESS candidates, and degradation segments, although the feeder topology remains fixed across candidate evaluations.

These figures demonstrate tested-scale executability, not a proof for networks with thousands of buses or arbitrary feeder types. The evidence is further bounded by balanced positive-sequence models, one national historical data package, finite candidate sets, and daily-equivalent economics. The DoD-dependent PWL model improves cycling-cost accounting but does not represent temperature, rate effects, or detailed electrochemistry.

Future work should examine unbalanced multiphase feeders, feeder-specific forecasts, longer chronological horizons, continuous or richer duration choices, detailed degradation states, and joint topology-storage planning. Each extension should retain the same audit principle: compare plans on explicitly controlled network, uncertainty, and economic definitions.

## 5. Conclusions

This study developed a historical-data-driven bilevel ESS planning framework with complete discrete upper-level evaluation and a fixed-topology DistFlow-based MISOCP operational model. Joint Year A load-PV-wind trajectories were reduced with input-risk-stratified historical-day selection, K10 was used for the principal 33-bus assessment, and K20 also selected the selected bus 24, 1.00 MW/2.00 MWh, 2 h design.

Relative to No ESS, the proposed 33-bus design reduced K10 TOTEX from 73,151.02 to 51,826.24 CNY/day, raised the minimum voltage from 0.94138 to 0.95405 p.u., and reduced UVE from 0.013528 to 0.001740 p.u.·h. Cross-year and seven-day tests preserved the principal economy-security pattern. The released 240-bus case study improved physical security and operating metrics at a small incremental economic cost. These findings support common-topology attribution, historical risk coverage and exact worst-day-retention auditing, and penalty-independent voltage metrics as practical elements of auditable ESS planning.

Within the fixed-topology and finite-scenario setting considered in this study, the numerical results remain case-specific. They do not establish a universal storage size or location or economic savings for all feeders. Their main implication is methodological: ESS plans should be compared on a common network, evaluated across credible historical conditions, and judged through both total expenditure and physical security outcomes.

## Supporting information

S1 Table Search-performance comparison of PSO, GA, and DE under a common candidate-evaluation budget. (DOCX)

S1 Data Source data underlying Tables 1–20, S1 Table, and Figs 1–22. (ZIP)

## Abbreviations

ADN: Active Distribution Network
BESS: Battery Energy Storage System
CAPEX: Capital Expenditure
CB: Capacitor Bank
CRF: Capital Recovery Factor
DE: Differential Evolution
DER: Distributed Energy Resource
ESS: Energy Storage System
GA: Genetic Algorithm
IEEE: Institute of Electrical and Electronics Engineers
MVM: Mean Voltage Margin
OPEX: Operating Expenditure
PSO: Particle Swarm Optimization
PV: Photovoltaic
SOCP: Second-Order Cone Programming
TOTEX: Total Expenditure
UVH: Undervoltage Hours
D_max_: Maximum Undervoltage Depth
UVE: Cumulative Undervoltage Exposure
WT: Wind Turbine
MT: Microturbine
PHEV: Plug-in hybrid electric vehicle
V2G: Vehicle-to-grid
G2V: Grid-to-vehicle
YALMIP: Yet Another LMI Parser
VoLL: Value of Lost Load
SCF: Single-Commodity Flow
ECDF: Empirical Cumulative Distribution Function
SVC: Static Var Compensator
TOU: Time-of-Use
CV: Coefficient of Variation
