# Supplementary material for: Uncertainty-aware bilevel energy storage planning for active distribution networks: A fixed-topology DistFlow-based MISOCP framework with voltage-security assessment
Source: PLoS One. 2026 Oct 9;21(10):e0353192. doi: 10.1371/journal.pone.0353192 (PMC13652237; doi:10.1371/journal.pone.0353192)
Supplement: Supplementary file 1 [file pone.0353192.s001.docx]

**S1 Table.** Search-performance comparison of PSO, GA, and DE under a common candidate-evaluation budget.

| **Algorithm** | **Runs** | **Completed** | **Grid-best hit (%)** | **Top-5 hit (%)** | **Median evaluations to best** | **Median regret (CNY)** |
| --- | --- | --- | --- | --- | --- | --- |
| PSO | 30 | **30** | **86.67** | **100** | **20** | **0** |
| GA | 30 | **30** | 80.00 | **100** | 24.5 | **0** |
| DE | 30 | 21 | 43.33 | 93.33 | 26.5 | 61.3863 |

**Note:** PSO, GA, and DE are evaluated under a common candidate-evaluation budget. This benchmark is reported in Supporting Information because the primary planning conclusions are based on the discrete-domain evaluation. Boldface indicates the best value(s) for the reported search-performance metrics; ties are shown in bold for all tied methods.
