# Supplementary material for: Uncertainty-aware bilevel energy storage planning for active distribution networks: A fixed-topology DistFlow-based MISOCP framework with voltage-security assessment
Source: PLoS One. 2026 Oct 9;21(10):e0353192. doi: 10.1371/journal.pone.0353192 (PMC13652237; doi:10.1371/journal.pone.0353192)
Supplement: Supplementary file 2 [file pone.0353192.s004.docx]

RESPONSE TO REVIEWERS

**PLOS ONE**

Manuscript ID: PONE-D-26-30578

*Uncertainty-aware bilevel energy storage planning for active distribution networks: A fixed-topology DistFlow-based MISOCP framework with voltage-security assessment*

Dear Academic Editor and Reviewers,

We sincerely thank the Academic Editor and both reviewers for their careful and constructive assessment of our manuscript. In response, we substantially strengthened the uncertainty characterization, scenario-fidelity, input-risk, and exact worst-day-retention analysis, complete 33-bus candidate-domain evaluation, cross-year and interday validation, engineering-scale 240-bus study, economic and storage-model sensitivities, and reproducibility record. We also revised the contribution and limitations so that the claims remain aligned with the evidence.

For ease of reading, each comment is reproduced verbatim in blue and is followed by our response and the corresponding manuscript location. Location references use the final section, equation, figure, and table numbering. Figures and table extracts are included only where they present the relevant evidence directly.

Location references: All page and line references in this response refer to the final tracked manuscript submitted with this revision. They correspond to the continuous line numbers displayed in Microsoft Word Desktop with tracked changes shown (All Markup), including visible deleted and inserted text. Tables, figures, equations, and other embedded objects are referenced by their final identifiers and by the adjacent numbered manuscript lines where Word does not display separate line numbers for the object itself.

Journal Requirements

## Journal Requirement 1

**Reviewer comment (reproduced verbatim)**

1. Please ensure that your manuscript meets PLOS ONE's style requirements, including those for file naming.

**Response:**

We have organized the clean and tracked manuscripts and the accompanying submission files according to the requested PLOS ONE structure and naming conventions. The revised manuscript uses a consistent heading hierarchy, numbered equations, journal-style figure and table captions, and clearly separated end-matter sections.

The filenames identify the function of each file, and the title/author page and main-body organization were checked against the two formatting examples provided by the journal.

**Changes in the revised manuscript:**

The manuscript and submission files were organized and named consistently with the journal’s formatting instructions.

**Where this appears in the revised manuscript**

• Front matter, p. 1, Lines 2–11: final title, author and affiliation information, and correspondence details.

• Supporting information, p. 96: final S1 Table and S1 Dataset designations and captions.

## Journal Requirement 2

**Reviewer comment (reproduced verbatim)**

2. Please note that PLOS One has specific guidelines on code sharing for submissions in which author-generated code underpins the findings in the manuscript. In these cases, we expect all author-generated code to be made available without restrictions upon publication of the work. Please review our guidelines at https://journals.plos.org/plosone/s/materials-and-software-sharing#loc-sharing-code and ensure that your code is shared in a way that follows best practice and facilitates reproducibility and reuse.

**Response:**

We agree that the author-generated code underlying a computational planning study should be accessible and reusable. The code, input definitions, scenario-processing routines, network data, optimization settings, and result files supporting the revised manuscript are publicly available from the development repository and the version-specific Zenodo record. The archived record corresponding to this revision is Zenodo v1.0.1 (https://doi.org/10.5281/zenodo.21875850), and the development repository is https://github.com/mouzhenhua8/uncertainty-aware-bilevel-ess-planning.

Author-generated code is released under the MIT License. Author-generated data and documentation are released under CC BY 4.0; any third-party material remains subject to its original terms. The manuscript’s Data Availability statement now gives both access routes and explains the relationship between the versioned archive and the development repository.

**Changes in the revised manuscript:**

Added the public code and data access information, version-specific DOI, and license terms.

**Where this appears in the revised manuscript**

• Data Availability, pp. 84–85, Lines 1301–1312: public GitHub repository, version-specific Zenodo v1.0.1 DOI, material scope, and license terms.

• Supporting information, p. 96: S1 Dataset and S1 Table captions tied to the shared materials.

## Journal Requirement 3

**Reviewer comment (reproduced verbatim)**

3. Please provide a complete Data Availability Statement in the submission form, ensuring you include all necessary access information or a reason for why you are unable to make your data freely accessible. If your research concerns only data provided within your submission, please write "All data are in the manuscript and/or supporting information files" as your Data Availability Statement.

**Response:**

The Data Availability statement has been completed with direct access information. It identifies the version-specific Zenodo v1.0.1 record (https://doi.org/10.5281/zenodo.21875850), the development repository (https://github.com/mouzhenhua8/uncertainty-aware-bilevel-ess-planning), and the S1 Dataset supplied with the submission. The statement also distinguishes author-created material from third-party source data and retains the original terms that apply to the latter.

The same access information will be entered in the submission form. This wording is more informative than the generic statement that all data are contained in the article because the analyses depend on code, network inputs, historical-series processing, and supporting result files that are most useful when provided in their native directory structure.

**Changes in the revised manuscript:**

Completed the Data Availability statement with stable access information and licensing details.

**Where this appears in the revised manuscript**

• Data Availability, pp. 84–85, Lines 1301–1312: direct GitHub and Zenodo access information, material scope, and third-party-data terms.

## Journal Requirement 4

**Reviewer comment (reproduced verbatim)**

4. Please include captions for your Supporting Information files at the end of your manuscript, and update any in-text citations to match accordingly. Please see our Supporting Information guidelines for more information: http://journals.plos.org/plosone/s/supporting-information.

**Response:**

We added the Supporting Information captions at the end of the manuscript and aligned their names with the in-text references. The manuscript now identifies S1 Table as the search-performance comparison of PSO, GA, and DE under a common candidate-evaluation budget. It also includes the caption “S1 Dataset. Source data underlying Tables 1–19, S1 Table, and Figs 1–22.” The corresponding files use these same designations.

**Changes in the revised manuscript:**

Added and aligned the S1 Table and S1 Dataset captions and their in-text citations.

**Where this appears in the revised manuscript**

• Supporting information, p. 96: complete S1 Table and S1 Dataset captions, including source data for the manuscript figures and tables.

## Journal Requirement 5

**Reviewer comment (reproduced verbatim)**

5. If the reviewer comments include a recommendation to cite specific previously published works, please review and evaluate these publications to determine whether they are relevant and should be cited. There is no requirement to cite these works unless the editor has indicated otherwise.

**Response:**

Neither reviewer made citation of a particular publication a condition of revision. We nevertheless reconsidered the literature needed to position the study and expanded the comparison of related work in **Table** 1. The cited studies were selected because they bear directly on stochastic or robust ESS planning, bilevel planning, distribution-network expansion, unbalanced-network storage allocation, or dynamic reconfiguration. We also added the Open Power System Data source for the historical series and the PNNL storage-cost assessment as context for the economic assumptions. These citations are used to delimit the scope of our contribution rather than to imply endorsement by the reviewers.

**Changes in the revised manuscript:**

Expanded the directly relevant literature and added the historical-data and storage-cost sources.

**Where this appears in the revised manuscript**

• Introduction and Table 1 (Introduction: pp. 4–5, Lines 63–75; Table 1 caption: p. 9, Lines 152–153): bounded quantitative positioning against representative stochastic, robust, bilevel, expansion-planning, and reconfiguration studies.

• Section 2.1.2 and Fig 5, p. 14, Lines 221–231: Open Power System Data source and Year A/Year B construction.

• References 41 and 43 (Reference 41: p. 92, Lines 1438–1439; Reference 43: p. 92, Lines 1443–1445): historical-data and grid-storage cost sources used in the revision.

# Reviewer 1

## Reviewer 1 — Comment 1

**Reviewer comment (reproduced verbatim)**

Comment 1: Novelty compared with existing bilevel ESS planning studies needs stronger justification

The manuscript claims an integrated framework combining uncertainty modeling, fixed-topology attribution, DistFlow-SOCP, TOTEX, and voltage-security assessment. However, many of these components have already been investigated individually in previous studies. The authors should more clearly explain what fundamental methodological advancement is introduced beyond combining existing techniques.

In particular, the novelty should be distinguished from previous stochastic ESS planning, robust optimization, and bilevel distribution network expansion studies. A quantitative comparison table including recent ESS planning methods (objective function, uncertainty treatment, network model, optimization approach, and limitations) would strengthen the contribution.

**Response:**

We appreciate this comment because it prompted us to sharpen both the scope and the evidence for the manuscript’s contribution. The revision now states the distinctive contribution directly and then defines the boundary of the claim.

The distinctive contribution is a **controlled uncertainty-aware ESS planning and attribution framework**. No ESS, Fixed ESS, and Proposed ESS are evaluated on the same retained radial topology, with the same device set and engineering assumptions. This **common-topology controlled attribution** makes the economic and physical voltage-security differences attributable primarily to the ESS plan and uncertainty representation rather than confounding them with simultaneous network reconfiguration. The fixed topology serves this controlled-attribution objective; it is not presented as universally superior to adaptive operation.

The framework couples that controlled baseline with **historically grounded, input-risk-stratified representative-day construction** from **366 complete historical Year A days** represented as joint 72-dimensional load–PV–wind trajectories, season-specific high-risk pools, K = 3, 5, 10, and 20 fidelity checks, and empirical membership-based scenario weights. It also embeds **degradation-aware lower-level operational evaluation** consistently in the planning comparison: the network physics use fixed-topology DistFlow-SOCP, while the complete lower-level model is MISOCP because exact piecewise-linear DoD-dependent degradation segment-selection binaries are included. Economic and physical voltage-security metrics are reported separately so that the security evidence does not depend only on penalty terms.

For the declared 33-bus planning domain, **all 211 valid alternatives were evaluated**. The reported **bus 24, 1 MW/2 MWh design** is therefore the best evaluated solution within that finite domain. **PSO, GA, and DE are retained only as search-efficiency benchmarks**, not as the source of the reported optimum. This attribution is supported by the quantitative literature comparison in Table 1; the K3/K5/K10/K20 fidelity and runtime study; economy–security decomposition; deterministic and resource-ablation comparisons; sensitivity analyses; Year A/Year B and seven-day chronological assessments; and the released 240-bus engineering-scale validation.

Accordingly, the novelty claim is deliberately bounded. The contribution lies in the controlled combination, attribution logic, and multi-layer validation of these elements, rather than in claiming K-means, DistFlow-SOCP, MISOCP, bilevel planning, or standard metaheuristics themselves as new. We also do not claim universal superiority or guaranteed retention of unobserved future extremes.

**Changes in the revised manuscript:**

Reframed the contribution around controlled common-topology attribution; strengthened the quantitative Table 1 comparison; clarified complete finite-domain evaluation and the PSO/GA/DE benchmark role; stated the novelty boundary explicitly; and connected the validation evidence directly to the contribution.

**Where this appears in the revised manuscript**

• Introduction and Table 1 (Introduction: pp. 4–5, Lines 63–75; Table 1 caption: p. 9, Lines 152–153): controlled-attribution contribution and quantitative comparison with representative planning studies.

• Sections 2.1.1 and 2.2.1–2.2.3 (Section 2.1.1: pp. 12–13, Lines 179–212; Section 2.2.1: pp. 20–24, Lines 293–364; Section 2.2.2: pp. 24–30, Lines 365–470; Section 2.2.3: pp. 31–38, Lines 480–602): retained common topology, historical risk-stratified scenario construction, and the degradation-aware fixed-topology lower-level model.

• Sections 3.1 and 3.4–3.8 (Section 3.1: pp. 45–47, Lines 708–737; Section 3.4: pp. 55–58, Lines 868–904; Section 3.5: p. 58, Lines 905–910; Section 3.6: pp. 58–59, Lines 911–924; Section 3.7: pp. 59–62, Lines 925–963; Section 3.8: pp. 62–64, Lines 964–979): scenario-fidelity/runtime evidence, complete evaluation of 211 alternatives, economy–security and resource-ablation comparisons, search benchmarks, cross-year/seven-day/240-bus validation, and sensitivities.

• Discussion, Sections 4.1–4.5, and Conclusions (Section 4.1: pp. 73–74, Lines 1130–1140; Section 4.2: p. 74, Lines 1141–1148; Section 4.3: pp. 75–76, Lines 1170–1179; Section 4.4: p. 76, Lines 1180–1193; Section 4.5: p. 77, Lines 1194–1212; Conclusions: pp. 79–80, Lines 1240–1256): contribution boundary, transferability, and case-specific limitations.

*Excerpt from revised manuscript Table 1: “Comparison of representative studies related to uncertainty-aware ADN planning and ESS planning.”*

| **Study** | **Uncertainty treatment** | **Objective and ESS/network formulation** | **Validation** | **Key limitation / distinction** |
| --- | --- | --- | --- | --- |
| Lu et al. (2020) | Data-driven distributionally robust model calibrated from measured data | Minimization of ESS planning and operating costs with coordinated ESS operation in a high-PV distribution network | High-PV distribution-system case study | Strong data-driven robustness, but does not isolate ESS effects under a common fixed topology or provide cross-year voltage-risk validation |
| Li et al. (2022) | Uncertain DG output represented in bilevel planning | Joint DG/ESS investment and operating-economy optimization through bilevel siting/sizing with lower-level ESS operation and binary PSO | PG&E 69-bus distribution system | Uses bilevel joint planning, but ESS benefits are not attributed under one common fixed topology |
| Yuan et al. (2024) | Two-stage stochastic-robust treatment with representative scenarios and a worst-case uncertainty set | Minimization of distributed ESS investment and operating costs using MISOCP subproblems and metaheuristic/global search | 17-node test system | Emphasizes worst-case robustness; validation is smaller in scale and does not include common-topology cross-year assessment |
| Zhan et al. (2025) | Renewable variability embedded in stochastic dynamic operation | Dynamic ADN operation through coordinated reconfiguration, SOP/ESS control, learning-based topology decisions, and lower-level SOCP | Modified IEEE 33- and 69-bus systems | Uses adaptive topology, whereas the present study fixes the reference topology to isolate ESS effects |
| **This study** | Historical Year A/Year B load-PV-wind records; input-risk-stratified historical-day reduction; CVaR-based diagnostic tail-risk evaluation | Daily-equivalent TOTEX minimization with separate voltage-security assessment; upper-level discrete ESS siting/sizing and lower-level fixed-topology MISOCP with DistFlow-SOCP network physics; PSO/GA/DE used as search benchmarks | 33-bus main study with cross-year and 7-day interday validation; released 240-bus engineering-scale study | Common-topology, auditable attribution of ESS economic and physical-security effects across historical uncertainty, multiple horizons, and network scales |

## Reviewer 1 — Comment 2

**Reviewer comment (reproduced verbatim)**

Major Comment 2: The assumption of fixed topology limits the practical applicability of the proposed framework

The manuscript emphasizes fixed topology to avoid changing baselines and isolate ESS benefits. While this is useful for attribution, active distribution networks increasingly rely on joint optimization of ESS placement, network reconfiguration, and flexible resources.

The authors should discuss:

Under what practical conditions is topology assumed fixed?

How would the optimal ESS location change if reconfiguration is allowed?

Could the proposed approach underestimate the benefit of coordinated ESS and switching actions?

The limitation section acknowledges this issue, but further sensitivity analysis is required.

**Response:**

We thank the reviewer for identifying this important boundary of the study. We agree that fixed topology is a deliberate scope condition, not a generally optimal policy for every active distribution network. A common topology can be appropriate when the normal feeder state is prescribed by protection coordination, maintenance arrangements, regulation, or an operator’s switching policy. It is also useful when the scientific question is the incremental effect of storage on a given network. If each strategy were allowed to choose a different topology, changes in losses, voltage sensitivity, congestion, and the weak-bus pattern could not be attributed to storage alone.

In the revised formulation, the one-time topology-identification step establishes the radial reference, and the resulting branch states are retained for every subsequent ESS candidate. Fig 2 now makes this sequence explicit. This does not imply that reconfiguration lacks value; it ensures that No ESS, Fixed ESS, and Proposed ESS face the same electrical network. Consequently, the K10 differences in TOTEX and voltage exposure are not caused by assigning more favorable switch states to one strategy.

Allowing reconfiguration could change both location and size. Switching changes electrical distance, upstream loading, loss gradients, weak-bus patterns, and the marginal voltage effect of active and reactive injections. A storage unit preferred at bus 24 on the retained network could move toward another weak or congested area after branch states change. Coordinated switching might also reduce the storage capacity needed to meet a voltage criterion, or it might increase the value of storage by combining spatial power-flow redistribution with temporal energy shifting. The fixed-topology study can therefore underestimate switching–storage synergies.

We considered the requested sensitivity carefully. A defensible joint comparison would require branch-status decisions, radiality and connectivity constraints, limits on switching frequency, switching costs and wear, protection feasibility, and a common control policy. Introducing only a few unconstrained switch variables would not represent topology–storage co-optimization and could create a misleading benchmark. We therefore retain the controlled common-topology comparison, explicitly state that the reported ESS locations are conditional on the retained feeder configuration, and identify a fully parameterized joint topology–storage formulation as a valuable extension.

This limitation now appears with the result rather than only as a brief closing caveat. The 33-bus and 240-bus cases support storage siting and economy–security comparisons on fixed radial feeders. They do not show what the optimum would be under unrestricted switching. This wording preserves the value of a common counterfactual while making the practical boundary of the finding clear.

**Key evidence supporting this response**

• Fig 2 documents one-time topology identification and the fixed radial topology held common across all later 33-bus comparisons

• No ESS, Fixed ESS, and Proposed ESS are evaluated on the same retained branch-state vector, isolating the storage-plan effect

• The released 240-bus study repeats the fixed-radial attribution logic, with conclusions explicitly conditioned on that topology

**Changes in the revised manuscript:**

Expanded the practical basis for a fixed topology, explained how reconfiguration could change storage siting and value, and stated that coordinated benefits may be underestimated.

**Where this appears in the revised manuscript**

• Section 2.1.1 and Fig 2, pp. 12–13, Lines 179–212 (Fig 2 caption: p. 12, Lines 189–190): one-time topology identification and the fixed radial network used as the common attribution baseline.

• Discussion, Section 4.4, p. 76, Lines 1180–1185: practical conditions for fixed topology and the possibility of underestimated switching–storage synergies.

• Discussion, Section 4.5, p. 77, Lines 1209–1212: joint topology–storage optimization identified as a separate extension.

*Reproduced from the revised manuscript: Fig 2. One-time topology identification for the IEEE 33-bus feeder: (a) initial feeder and switching actions; (b) fixed radial topology used in subsequent simulations.*

## Reviewer 1 — Comment 3

**Reviewer comment (reproduced verbatim)**

Major Comment 3: The uncertainty generation methodology requires more detailed validation

The proposed method relies on Monte Carlo sampling and scenario reduction (600 samples reduced to 5 scenarios). However, the manuscript does not sufficiently demonstrate whether five scenarios are enough to represent uncertainty.

The authors should provide:

Comparison between original Monte Carlo results and reduced scenarios.

Error evaluation after scenario reduction.

Sensitivity analysis using different numbers of scenarios (e.g., K = 3, 5, 10, 20).

Explanation of why the selected tail scenario probability floor is appropriate.

Currently, the conclusion that tail risks are preserved is not sufficiently demonstrated.

**Response:**

We thank the reviewer for pressing us to demonstrate scenario fidelity rather than assume it. This comment prompted a major change to the uncertainty analysis. The revised manuscript no longer uses 600 generated Monte Carlo samples reduced only to five scenarios. Instead, it begins with **366 complete, jointly aligned historical days in Year A**. Each day is represented by a **72-dimensional vector containing 24 hourly load, 24 PV, and 24 wind observations**, and no additional synthetic trajectories are introduced.

We now compare K = 3, 5, 10, and 20 representative-day sets against the retained historical record. The final builder uses Year A inputs only. Its combined input-risk score is formed from standardized peak net load, low renewable availability, maximum absolute net-load ramp, and the fixed-topology voltage-stress proxy. For each season s, the high-risk pool $H_{s}$ contains the top ceil(0.10 $N_{s}$) historical days by that score, where $N_{s}$ is the number of complete Year A days in season s. K3 uses three global medoids; K5 uses one ordinary medoid per season plus the annual combined-input-risk maximum; K10 uses one ordinary and one high-risk medoid per season plus the annual high-load/low-renewable and annual high-ramp maxima; and K20 retains the K10 core and adds ten constrained seasonal ordinary medoids using the frozen Hamilton allocation. Each historical day is assigned exactly once to its nearest eligible representative. If $n_{k}$ is the resulting membership count, $\pi_{k}=\frac{n_{k}}{N_{A}}=\frac{n_{k}}{366}$; no probability floor or manual inflation is applied. The full-history worst-voltage date is audited after selection rather than used as a selection oracle: it is absent from K3 and appears as an actual representative trajectory in K5, K10, and K20.

**Table** 4 reports the requested errors and computation times. K3 gives a maximum TOTEX error of 15.60% and a maximum UVE error of 64.73%, and it does not retain the exact worst-voltage day. K5 retains that day, but its maximum TOTEX and UVE errors are 47.65% and 149.85%. **K10 also retains the day and lowers those errors to 12.49% and 42.69%, with a runtime of 911.7 s**. **K20 provides the closest tested representation, with errors of 1.40% and 11.13%, but its runtime increases to 2771.3 s**. We report the unexpectedly poor K5 aggregate errors rather than implying that more clusters improve every metric monotonically.

**K10 is used for the principal 33-bus comparison** because it retains the observed worst-voltage day and the selected bus 24, 1 MW/2 MWh design while providing a practical middle point in the tested fidelity–time trade-off. K20 and full-history calculations provide higher-fidelity confirmation; K10 is not described as the lowest-error representation. **Fig** 6 compares the historical daily cloud with the K10 trajectories, and **Fig** 7 displays the scenario-count errors and runtimes. Tables 5 and 6 add design-stability and operating comparisons, while Year B supplies an independent cross-year test.

The revision now distinguishes input-side risk stratification from post-selection exact-date retention. The construction does not guarantee that every future extreme is represented, and it is not a formal distributionally robust optimization model. It is an empirical historical-scenario protocol conditional on the two-year source record, daily vectorization, frozen input features, and fixed distance and selection rules. Those limits are stated in the Discussion.

**Key evidence supporting this response**

• Each seasonal risk pool contains the top ceil(0.10$N_{s}$) Year A days by the four-feature input-risk score; probabilities are

$\pi_{k}=\frac{n_{k}}{366}$

• K10 retains the exact worst-voltage date with 12.49% maximum TOTEX error, 42.69% maximum UVE error, and 911.7 s runtime

• K20 lowers the corresponding errors to 1.40% and 11.13% and confirms the same bus 24, 1.00 MW/2.00 MWh, 2 h plan

**Changes in the revised manuscript:**

Replaced the former synthetic N=600/K=5 construction with historical joint daily trajectories and quantitative K=3/5/10/20 fidelity, input-risk-stratification, exact-date-retention, design-stability, and runtime comparisons.

**Where this appears in the revised manuscript**

• Section 2.2.2, Eqs (10)–(19), pp. 24–30, Lines 365–470: 72-dimensional historical vectors, four-feature input risk, seasonal ceil(0.10 $N_{s}$) pools, K-specific rules, assignment, and $\pi_{k}=\frac{n_{k}}{366}$ weights.

• Section 3.1, Figs 6–7 and Tables 4–6, pp. 45–47, Lines 708–737: K=3/5/10/20 fidelity, exact worst-date retention, runtime, and K20 design confirmation.

• Discussion, Section 4.2, p. 74, Lines 1141–1148: K10 as the principal computational compromise and K20/full-history evidence as complementary checks.

*Reproduced from the revised manuscript: Fig 6. Historical Year A daily trajectories and the input-risk-stratified K=10 representative set for load, photovoltaic, and wind profiles.*

*Reproduced from the revised manuscript: Fig 7. Scenario-count fidelity and computational trade-off for K=3, 5, 10, and 20 representative days.*

*Relevant results from revised manuscript Table 4: “Scenario-reduction fidelity and computational performance for K = 3, 5, 10, and 20.”*

| **K** | **Max TOTEX error (%)** | **Max UVE error (%)** | **Max \|**$\boldsymbol{V}_{\boldsymbol{min}}$ **error\| (p.u.)** | **Worst historical day retained** | **Runtime (s)** |
| --- | --- | --- | --- | --- | --- |
| 3 | 15.60 | 64.73 | 0.02052 | No | **187.8** |
| 5 | 47.65 | 149.85 | **7.12×10⁻⁵** | Yes | 448.4 |
| 10 | 12.49 | 42.69 | 6.66×10⁻⁴ | Yes | 911.7 |
| 20 | **1.40** | **11.13** | 6.35×10⁻⁴ | Yes | 2771.3 |

## Reviewer 1 — Comment 4

**Reviewer comment (reproduced verbatim)**

Major Comment 4: The renewable and load uncertainty models appear highly synthetic

The uncertainty model uses generated PV, wind, and load trajectories. However, realistic distribution planning requires historical or forecast-based uncertainty characteristics.

The authors should clarify:

Are the uncertainty distributions derived from real operational data?

How are seasonal variations considered?

How are extreme renewable events represented?

Since the main contribution depends strongly on uncertainty effects, unrealistic uncertainty assumptions may significantly influence the ESS value estimation.

**Response:**

We appreciate the reviewer’s focus on the realism of the uncertainty inputs, because it is central to the value attributed to storage. The revised analysis therefore does not use generated load, PV, or wind distributions. It uses the Open Power System Data time-series package for Denmark: 2016 is designated Year A and 2017 Year B. After time alignment and completeness checks, the study retains **366 complete Year A days and 364 complete Year B days**. Load, PV, and wind remain paired by hour and day, so their observed temporal dependence is preserved rather than reconstructed from independent marginal distributions.

Seasonal variation enters through the full calendar records. Year A is used to construct the representative daily sets and to scale the benchmark feeder; Year B is held apart for cross-year validation. **Fig 5** shows the mean daily profiles, hourly interquartile ranges, and time-of-use tariff for both years. The annual calculations then evaluate all complete days in each year, rather than interpreting a single representative day as an annual record. The **separate seven-day calculation carries state of charge from one day to the next** and allows us to compare chronological coupling with daily resetting.

Extreme events are empirical, not generated. For each season s, the final builder defines the high-risk pool as the top ceil(0.10 $N_{s}$) historical days by the frozen combined input-risk score, where $N_{s}$ is the number of complete Year A days in that season, and it adds annual input-side maxima according to the declared K-specific rules. The scenario-count study then records whether the full-history worst-voltage date is retained exactly and measures TOTEX, UVE, and voltage-ranking error against the full-history reference. This protocol covers extreme conditions present in the source record but does not create unobserved weather extremes or assign return periods beyond the two-year record. We have made that boundary explicit.

The annual results support the direction of the principal comparison across both years. Proposed ESS gives mean daily TOTEX values of **51.91 thousand CNY/day in Year A and 51.56 thousand CNY/day in Year B**, with the lowest mean shed energy among the three strategies in both years. The **worst minimum voltages are 0.95395 and 0.95369 p.u., respectively**. These checks do not turn the source series into measurements from the specific IEEE 33-bus feeder; the public national profiles are scaled to the benchmark network. We now say so directly and avoid describing the inputs as site-specific measurements.

For transfer to a utility feeder, the same procedure should be populated with local demand, renewable, tariff, and device data. The revised evidence is stronger because it preserves real joint temporal patterns and separates construction and validation years, while the conclusion remains appropriately limited to the historical record and scaling used in the study.

**Changes in the revised manuscript:**

Documented the OPSD Year A/Year B source, retained joint temporal dependence and empirical extremes, and added cross-year and short-chronology validation with explicit limitations.

**Where this appears in the revised manuscript**

• Section 2.1.2 and Fig 5, p. 14, Lines 221–231: paired Year A/Year B load–PV–wind records and seasonal distributions.

• Section 2.2.2, Eqs (10)–(19), pp. 24–30, Lines 365–470: input-risk-stratified historical-day construction without synthetic trajectories.

• Section 3.6, Figs 16–18 and Table 14, pp. 58–59, Lines 911–924: complete Year A/Year B and seven-day interday-SOC validation.

• Discussion, Sections 4.2, 4.4, and 4.5 (Section 4.2: p. 74, Lines 1141–1148; Section 4.4: p. 76, Lines 1190–1193; Section 4.5: p. 77, Lines 1204–1212): empirical coverage, chronology, and transferability boundaries.

*Reproduced from the revised manuscript: Fig 5. Mean daily Year A and Year B load, PV, and wind profiles and the time-of-use tariff. Shaded areas are hourly 25th–75th percentile ranges.*

## Reviewer 1 — Comment 5

**Reviewer comment (reproduced verbatim)**

Major Comment 5: The economic assumptions for ESS investment need stronger justification

The ESS planning result strongly depends on CAPEX, degradation cost, discount rate, and lifetime assumptions. The manuscript uses:

Power cost = 1500 CNY/kW

Energy cost = 1800 CNY/kWh

Discount rate = 8%

Lifetime = 10 years

However, these assumptions are not sufficiently justified.

The authors should provide:

Sources of ESS cost parameters.

Sensitivity analysis for future battery cost reduction.

Impact of different lifetime assumptions.

Influence of degradation cost coefficients.

Without this analysis, the economic conclusions may not be transferable.

**Response:**

We thank the reviewer for asking us to strengthen the economic basis. The 1500 CNY/kW, 1800 CNY/kWh, and 10-year values quoted in the comment belonged to an earlier model version and are not used in the current manuscript. The final power–energy cost split was derived from paired 2025 CNESA LFP EPC benchmark averages of 1043.82 CNY/kWh for a 2-hour system and 935.40 CNY/kWh for a 4-hour system (source record: https://research.cnesa.org/report/info/detail/?id=6645). With total specific CAPEX written as $T\left( H \right)=\frac{c_{P}}{H}+c_{E}$, these two observations give $c_{E}=2T\left( 4 \right)-T\left( 2 \right)=826.98$ CNY/kWh and $c_{P}=2\left[ T\left( 2 \right)-c_{E} \right]=433.68$ CNY/kW. Because the source values are already denominated in CNY, no USD-to-CNY conversion was applied to these CAPEX coefficients.

Table 2 reports the resulting **433.68 CNY/kW and 826.98 CNY/kWh coefficients**, together with fixed O&M of 33.036484 CNY/kW-year, **an 8% discount rate, and a 15-year project life**. The fixed O&M value originates from the 4.59 USD/kW-year PNNL benchmark and was converted with the recorded 2024 average exchange rate of 7.19749110789917 CNY/USD; PNNL is also retained as broader storage-cost and performance context. The 8% discount rate and 15-year life are declared case-study planning assumptions, applied consistently across all strategies rather than presented as universal market values.

The revision then tests how the conclusion responds to those assumptions. Table 18 and Fig 22 vary the **battery-cost multiplier from 0.50 to 1.15**; the retained design’s TOTEX ranges from 51,492.13 to 51,926.47 CNY/day. **Project life is varied from 8 to 15 years**, giving 52,153.31 CNY/day at 8 years and 51,826.24 CNY/day at 15 years. The discount-rate analysis spans 4% to 12% and re-evaluates the discrete alternatives: **4% and 6% select bus 24, 1 MW/4 MWh, whereas 8%, 10%, and 12% select bus 24, 1 MW/2 MWh**, with TOTEX values from 51,611.99 to 51,997.80 CNY/day.

We also vary the degradation-cost multiplier from 0.50 to 1.50 and allowable depth of discharge from 60% to 80%. The degradation cases span 51,767.10 to 51,901.10 CNY/day, while the DoD cases give 52,070.93, 51,937.83, and 51,826.24 CNY/day at 60%, 70%, and 80%. Because the upper-level design space is discrete and dispatch changes with the assumptions, the evaluated response need not be perfectly smooth or monotonic.

Duration is examined directly: the best tested two-, three-, and four-hour designs have TOTEX values of 51,826.24, 51,888.42, and 51,887.63 CNY/day. Their proximity and the H2/H4 switch under discount-rate sensitivity show that local costs and financing can affect the near-optimal ranking. Accordingly, the Discussion asks users to substitute local procurement costs, tariffs, financing, lifetime, degradation, and operating rules. The results support the economy–security conclusion only within the tested ranges; they do not make one storage configuration transferable to every market or technology.

**Key evidence supporting this response**

• Table 2 reports the power-cost, energy-cost, fixed-O&M, discount-rate, and project-life assumptions used in every comparison

• Fig 22 and Table 18 cover battery cost, lifetime, discount rate, degradation cost, DoD, and duration sensitivities

• The 4% and 6% discount-rate cases select the four-hour alternative, making the observed H2/H4 ranking change explicit

**Changes in the revised manuscript:**

Updated the economic parameter basis and added battery-cost, lifetime, discount-rate, degradation-cost, DoD, and duration sensitivities.

**Where this appears in the revised manuscript**

• Table 2, p. 15, adjacent Line 232: final power cost, energy cost, fixed O&M, discount rate, and project-life assumptions.

• Section 3.8, Fig 22 and Table 18, pp. 62–64, Lines 964–979: battery-cost, lifetime, discount-rate, degradation, DoD, and duration sensitivities.

• Section 2.3 (economic assumptions: pp. 40–41, Lines 630–633 and 642–651; References 43–44: p. 92, Lines 1443–1448): economic assumptions defined as localizable planning inputs; References 43–44 provide the cited cost context.

*Excerpt from revised manuscript Table 18: “Economic and storage-model sensitivity results.”*

| **Sensitivity factor** | **Level** | **TOTEX (CNY/day)** | **Design / evaluation basis** |
| --- | --- | --- | --- |
| **Battery-cost multiplier** | 0.50 | 51492.13 | Proposed ESS: Bus 24, 1.00 MW / 2.00 MWh (H2) |
| Battery-cost multiplier | 1.00 | 51826.24 | Proposed ESS: Bus 24, 1.00 MW / 2.00 MWh (H2) |
| Battery-cost multiplier | 1.15 | 51926.47 | Proposed ESS: Bus 24, 1.00 MW / 2.00 MWh (H2) |
| **Project lifetime** | 8 years | 52153.31 | Proposed ESS: Bus 24, 1.00 MW / 2.00 MWh (H2) |
| Project lifetime | 15 years | 51826.24 | Proposed ESS: Bus 24, 1.00 MW / 2.00 MWh (H2) |
| **Discount rate** | 4% | 51611.99 | Best design: Bus 24, 1.00 MW / 4.00 MWh (H4) |
| Discount rate | 8% | 51826.24 | Best design: Bus 24, 1.00 MW / 2.00 MWh (H2) |
| Discount rate | 12% | 51997.80 | Best design: Bus 24, 1.00 MW / 2.00 MWh (H2) |

*Reproduced from the revised manuscript: Fig 22. Combined economic, ESS-model, risk, voltage-threshold, and representative-scenario sensitivity analysis.*

## Reviewer 1 — Comment 6

**Reviewer comment (reproduced verbatim)**

Major Comment 6: The voltage-security improvement requires more rigorous interpretation

The manuscript reports improvements in undervoltage hours, depth, and accumulated undervoltage energy. For example, stochastic ESS reduces UVH from 17 h to 14 h and improves voltage-related indicators.

However, the actual voltage violations appear relatively small (for example, minimum voltage deviations are close to the threshold). The authors should discuss:

Whether these improvements are practically significant.

Whether the voltage-security benefit justifies ESS investment.

Comparison with alternative voltage control methods such as OLTC, inverter reactive power control, or capacitor switching.

**Response:**

We thank the reviewer for asking us to interpret voltage improvement in physical and practical terms rather than through a monetary penalty alone. The numerical example in the comment corresponds to an earlier version. Under the current K10 evaluation, **No ESS has a minimum voltage of 0.94138 p.u., 1.582 undervoltage hours, a maximum undervoltage depth of 0.02862 p.u., and UVE of 0.013528 p.u.·h** at the 0.97 p.u. planning threshold. **Proposed ESS changes these values to 0.95405 p.u., 0.249 h, 0.01595 p.u., and 0.001740 p.u.·h**. The benefit therefore appears in the worst value, duration, depth, and accumulated exposure.

Fig 12 and Tables 12–13 now separate the engineering and economic evidence. No ESS has 52,389.81 CNY/day of non-security operating cost and 20,761.21 CNY/day of security penalties, giving **73,151.02 CNY/day TOTEX**. Proposed ESS has 49,482.58 CNY/day of non-security operating cost, 1,584.94 CNY/day of security penalties, and 758.72 CNY/day of annualized storage cost, giving **51,826.24 CNY/day TOTEX**. Thus the investment is not justified by a few milliper-unit of voltage improvement in isolation; within the formulated problem, it is supported by the combined operating-cost and security-exposure changes.

The Fixed ESS case is an important qualification. It reduces undervoltage hours to 1.224 h and UVE to 0.007272 p.u.·h, but its minimum voltage is 0.94081 p.u., slightly below the No ESS value. This counterexample shows why no single voltage statistic is sufficient and why siting and sizing matter. We therefore report all physical measures and avoid saying that every storage placement improves every voltage index. Threshold sensitivity at 0.96 and 0.97 p.u. further distinguishes the physical voltage from the planning criterion used to classify an exposure.

We also agree that OLTCs, switched capacitor banks, SVCs, and inverter reactive-power control can be alternatives or complements. The modeled network contains specified flexible and reactive resources, and the resource ablation results show their role within the retained configuration. A fair investment comparison with ESS would require device-specific CAPEX and O&M, switching limits and wear, reactive-power capability, control and coordination policies, lifetime, and reliability treatment. Without that common basis, claiming superiority for ESS would not be justified.

The revised Discussion consequently makes a bounded claim: the proposed storage plan improves the tested economy–security trade-off for this fixed network and resource set. It does not establish that ESS should replace OLTC, capacitor, or inverter controls in general. This wording gives the observed voltage changes their proper engineering significance without extending the comparison beyond the technologies actually evaluated.

**Changes in the revised manuscript:**

Added penalty-independent voltage metrics and a separated cost decomposition, and limited the interpretation relative to voltage-control technologies not compared on a common basis.

**Where this appears in the revised manuscript**

• Sections 3.4.2–3.4.3, Fig 12, and Tables 12–13, pp. 56–57, Lines 880–895: separated cost components and penalty-independent Vmin, UVH, Dmax, UVE, MVM, and shedding metrics.

• Discussion, Section 4.3, pp. 75–76, Lines 1170–1179: physical voltage interpretation and the boundary of comparisons with alternative voltage-control technologies.

*Reproduced from the revised manuscript: Fig 12. K10 cost and voltage-security decomposition for No ESS, Fixed ESS, and Proposed ESS.*

*Relevant results from revised manuscript Table 12: “TOTEX and cost breakdown for the three 33-bus planning strategies.”*

| **Strategy** | **Non-security operating cost (CNY/day)** | **Security penalties** | **Annualized ESS cost** | **TOTEX** |
| --- | --- | --- | --- | --- |
| No ESS | 52389.81 | 20761.21 | 0.00 | 73151.02 |
| Fixed ESS | 51501.84 | 7178.09 | 303.49 | 58983.43 |
| Proposed ESS | **49482.58** | **1584.94** | 758.72 | **51826.24** |

*Relevant results from revised manuscript Table 13: “Penalty-independent voltage-security metrics for the three 33-bus planning strategies.”*

| **Strategy** | $\boldsymbol{V}_{\boldsymbol{min}}$ **(p.u.)** | **UVH (h)** | $\boldsymbol{D}_{\boldsymbol{max}}$ **(p.u.)** | **UVE (p.u.·h)** | **MVM (p.u.)** | **Load shedding (MWh)** |
| --- | --- | --- | --- | --- | --- | --- |
| No ESS | 0.94138 | 1.582 | 0.02862 | 0.013528 | 0.007112 | 0.064617 |
| Fixed ESS | 0.94081 | 1.224 | 0.02919 | 0.007272 | 0.007428 | 0.011744 |
| Proposed ESS | **0.95405** | **0.249** | **0.01595** | **0.001740** | **0.008688** | **0.004297** |

## Reviewer 1 — Comment 7

**Reviewer comment (reproduced verbatim)**

Major Comment 7: The optimization algorithm comparison is insufficient

The manuscript compares PSO, DE, and GA. However, these algorithms are heuristic methods and their performance depends strongly on parameter settings.

The authors should provide:

Population size.

Iteration number.

Parameter tuning procedure.

Stopping criteria.

Number of independent runs.

Additionally, comparison with deterministic optimization methods or mathematical programming approaches would strengthen confidence in the obtained ESS sizing solution.

**Response:**

We appreciate the reviewer’s request for a more rigorous basis for confidence in the selected design. We have clarified the role of the three heuristics and supplied the requested settings. Most importantly, PSO, GA, and DE do not determine the reported 33-bus planning result. We evaluated every one of the 211 valid alternatives in the finite siting–power–duration domain. The resulting complete objective surface provides an exhaustive-enumeration reference: bus 24, 1 MW/2 MWh is the best evaluated design in that declared domain. The heuristic study then asks how often each method finds that known point under the same limited candidate budget.

All three methods use a **population size of 6 and 30 independent runs**, with the same sequence of random seeds (2026072801–2026072830) used across PSO, GA, and DE. A run stops after **60 unique valid candidate evaluations** or 1,200 total proposals; repeated candidates do not consume the unique-evaluation allowance. PSO uses inertia 0.72 and cognitive and social coefficients of 1.49 and 1.49. GA uses crossover probability 0.90, mutation probability 0.10 per dimension, binary tournament selection, and elitist replacement. DE uses rand/1/bin mutation with F = 0.70 and CR = 0.90.

The intention of these settings is a common comparison, not separate tuning to favor one method. Every algorithm queries the same saved objective surface; no lower-level model is re-solved during the search comparison. This removes variations in optimization time or numerical tolerances from the comparison and makes global-hit rate, top-five hit rate, evaluations to first optimum, and terminal regret directly measurable. The seeds, settings, and stopping rules are included with the shared material.

S1 Table gives the 30-run results. **PSO reaches the known best candidate in 86.67% of runs** and a top-five candidate in 100%, with a median of 20 unique evaluations to the first optimum. **GA gives 80.00%**, 100%, and 24.5 evaluations. **DE gives 43.33%**, 93.33%, and 26.5 evaluations; 21 runs meet the stopping condition, and its median terminal regret is 61.3863 CNY/day. These figures describe behavior on this finite surface under the stated budget. We do not generalize them into an overall ranking of PSO, GA, and DE.

For this 33-bus discrete planning problem, complete enumeration is stronger evidence for the chosen design than another heuristic curve or a surrogate mathematical comparison because the best point in the stated domain is known. Each candidate’s lower-level operating problem is solved through the stated mathematical formulation. For larger or continuous upper-level domains, decomposition, branch-and-bound, screening, or surrogate methods could be appropriate, but those approaches are outside the present tests. The revised paper now separates confidence in the storage design from the narrower question of budgeted search behavior.

**Key evidence supporting this response**

• The complete 33-bus domain contains 211 valid alternatives and supplies the known grid optimum used only to benchmark search efficiency

• Under the common evaluation budget, grid-best hit rates are 86.67% for PSO, 80.00% for GA, and 43.33% for DE

• S1 Table reports 30-run completion, top-5 hit rate, evaluations to first optimum, and terminal regret for all three algorithms

**Changes in the revised manuscript:**

Specified population, budgets, seeds, stopping rules, and method parameters, and separated the heuristic comparison from the complete 211-alternative planning result.

**Where this appears in the revised manuscript**

• Section 2.3, pp. 43–44, Lines 678–690: common PSO/GA/DE settings, budgets, seeds, stopping criteria, and solver tolerances.

• Section 3.5 and S1 Table (Section 3.5: p. 58, Lines 905–910; S1 Table: p. 96, Lines 1572–1576): complete GRID211 optimum and 30-run search-efficiency results for PSO, GA, and DE.

*Reproduced from revised manuscript S1 Table: “Search-performance comparison of PSO, GA, and DE under a common candidate-evaluation budget.”*

| **Algorithm** | **Runs** | **Completed** | **Grid-best hit (%)** | **Top-5 hit (%)** | **Median evaluations to best** | **Median regret (CNY)** |
| --- | --- | --- | --- | --- | --- | --- |
| PSO | 30 | **30** | **86.67** | **100** | **20** | **0** |
| GA | 30 | **30** | 80.00 | **100** | 24.5 | **0** |
| DE | 30 | 21 | 43.33 | 93.33 | 26.5 | 61.3863 |

## Reviewer 1 — Comment 8

**Reviewer comment (reproduced verbatim)**

Major Comment 8: The scalability of the proposed bilevel framework is unclear

The study is demonstrated only on a modified IEEE 33-bus feeder. Modern distribution networks may contain hundreds or thousands of buses.

The authors should provide computational complexity analysis:

How does computation time increase with network size?

How many upper-level optimization evaluations are required?

Can the method handle multi-day or seasonal horizons?

The current validation does not demonstrate practical scalability.

**Response:**

We thank the reviewer for challenging the scalability evidence. The 33-bus case alone would not justify a statement about larger-network executability. The revision therefore includes a released 240-bus radial feeder as a separate engineering-scale study. The case has **240 buses, 239 retained branches, and 16 pre-specified ESS candidate locations**. Across the tested operating models, the formulation contains **139,000 rows, 259,848 columns, 693,648 nonzero coefficients, and 83,136 second-order-cone or quadratic constraints**.

The reported **14.04-31.10 s values are solver runtimes for complete K8 multi-scenario case evaluations** on the 240-bus feeder; each case spans 8 representative days × 24 h = 192 scenario-hour points. The 192-per-strategy and 768-total counts refer to AC back-substitution audit points, not 192 or 768 separate optimization runs. For the 33-bus system, **1286.78 s is the solver runtime for one selected-design K10 evaluation** spanning 10 representative days × 24 h; it is not the elapsed time for the complete 211-candidate planning screen. The maximum SOCP-to-AC voltage mismatch is 2.13 × 10⁻⁸ p.u., the 240-bus maximum physical residual is 9.99 × 10⁻⁷, and the 33-bus maximum physical residual is 9.73 × 10⁻⁹. These values provide model-size, per-case runtime, and numerical-consistency evidence rather than a bus-count scaling law.

At the upper level, **154 positive-capacity designs plus the no-build option give 155 alternatives** in the 240-bus candidate set. The paper distinguishes this finite siting study from an unrestricted search over all buses, powers, and durations. The location comparisons—reference, load-center, and feeder-head cases—show that placement continues to affect the economy–security balance on the larger feeder, while the separate severe-stress comparison checks the same qualitative pattern under a more demanding condition.

Computation does not scale with bus count alone. For a radial DistFlow-SOCP model, continuous network variables and branch constraints grow approximately with buses, branches, hours, and scenarios. Runtime can grow more sharply because it also depends on integer variables, cone structure, candidate count, conditioning, and solver behavior. Upper-level effort is roughly the number of candidate evaluations multiplied by the cost of each declared multi-scenario evaluation, although parallel execution and screening can change elapsed time. We added this explanation to Section 4.5.

The temporal scope is tested at three levels: representative 24-hour days for the principal planning calculation, all complete days in Year A and Year B for annual and cross-year evaluation, and a seven-day sequence with interday SOC coupling. These checks demonstrate multi-day and seasonal evaluation without claiming that we solved a joint 8,760-hour planning problem. Likewise, the 240-bus study shows executability at the released scale, not that the same formulation will be tractable on every thousand-bus network. The revised language now keeps that distinction explicit.

**Key evidence supporting this response**

• The released 240-bus instance reports 139,000 rows, 259,848 columns, 693,648 nonzeros, and 83,136 conic/quadratic constraints

• The finite screen contains 154 positive-capacity designs plus one no-build alternative; 768 denotes AC-audit scenario-hour points, not optimization runs

• Per-case K8 runtimes are 14.04–31.10 s, with 2.13×10⁻⁸ p.u. maximum SOCP-to-AC voltage mismatch and 9.99×10⁻⁷ maximum residual

**Changes in the revised manuscript:**

Added a released 240-bus study with model dimensions, per-case runtimes, candidate counts, AC-audit scenario-hour counts, and numerical-consistency measures, together with a bounded scaling discussion.

**Where this appears in the revised manuscript**

• Section 3.7, Figs 19–21 and Tables 15–17, pp. 59–62, Lines 925–963: released 240-bus candidate domain, economy–security comparisons, model dimensions, runtimes, and numerical consistency.

• Discussion, Section 4.5, p. 77, Lines 1194–1212: scaling drivers, bounded tested-scale claim, and possible extensions.

*Reproduced from the revised manuscript: Fig 20. Reference-versus-fixed-location comparison on the released 240-node feeder using Reference, load-center, and feeder-head locations.*

*Relevant results from revised manuscript Table 17: “Numerical consistency and computational performance of the 33-bus and released 240-bus systems.”*

| **System** | **Metric** | **Reported value** |
| --- | --- | --- |
| 33-bus | Maximum physical residual | 9.73×10⁻⁹ |
| 33-bus | Selected-design K10 evaluation runtime | 1286.78 s |
| 240-bus | Model size (rows / columns / nonzeros) | 139000 / 259848 / 693648 |
| 240-bus | Second-order-cone / quadratic constraints | 83136 |
| 240-bus | Per-case K8 evaluation runtime | 14.04–31.10 s |
| 240-bus | Maximum SOCP-to-AC voltage mismatch | 2.13×10⁻⁸ p.u. |
| 240-bus | Maximum physical residual | 9.99×10⁻⁷ |
| 240-bus | AC-audit scenario-hour points | 192 per strategy; 768 total |

## Reviewer 1 — Comment 9

**Reviewer comment (reproduced verbatim)**

Major Comment 9: The 24-hour scheduling horizon may underestimate long-term ESS behavior

The lower-level operation considers only a daily 24-hour horizon. However, ESS planning decisions involve:

Seasonal renewable variation.

Long-duration energy shifting.

Battery degradation over years.

Annual economic performance.

The authors should justify why a single-day horizon is sufficient for investment planning or provide additional multi-day/seasonal validation.

**Response:**

We appreciate the reviewer’s concern about the temporal evidence supporting a long-lived storage investment. A single generic day would indeed be inadequate. The principal optimization uses 24-hour representative days because the lower-level operating model is repeated across historically weighted daily conditions and because this keeps the finite planning study computationally manageable. That model uses a cyclic end-of-day SOC convention. We now state plainly that this convention does not represent seasonal energy carryover or long-duration storage across months.

To test whether the planning conclusion depends on one daily profile, the revision adds annual and cross-year evaluations. All **366 complete Year A days and 364 complete Year B days** are evaluated for No ESS, Fixed ESS, and Proposed ESS. Proposed ESS has mean daily TOTEX of **51.91 thousand CNY/day in Year A and 51.56 thousand CNY/day in Year B**, compared with 83.59 and 81.70 thousand CNY/day for No ESS. Its worst minimum voltage is **0.95395 p.u. in Year A and 0.95369 p.u. in Year B**, and its mean shed energy is 0.002318 and 0.000686 MWh/day. The same economy–security direction therefore persists across the construction and held-out years.

We also added a **separate seven-day chronological calculation** in which SOC carries from one day to the next. **Figs** 17–18 compare this interday treatment with the daily-reset convention for Fixed ESS and Proposed ESS. This test is important because daily resetting can suppress short sequences of charging or discharging conditions. It does not, however, create a full multiyear degradation trajectory or an 8,760-hour joint investment–operation model, and the paper no longer suggests otherwise.

Long-term economics enter through annualized CAPEX, the project lifetime, discount rate, fixed O&M, and the DoD-dependent cycle-degradation cost. Lifetime, discount-rate, degradation-cost, DoD, and duration sensitivities are reported in **Fig** 22 and **Table** 18. This provides evidence about investment assumptions and daily cycling, but it does not model calendar aging, temperature-driven aging, capacity fade over many years, or seasonal energy shifting. Those limitations are now stated in Section 4.5.

Our justification is therefore not that one 24-hour trajectory is sufficient. Rather, representative days are used for the principal finite-domain comparison and are supplemented by two complete calendar years and a chronologically linked week. The added tests support the reported plan for ordinary diurnal grid-side storage in the studied system, while longer-duration or seasonal storage would require a different horizon and intertemporal formulation.

**Key evidence supporting this response**

• The annual audit covers all 366 complete Year A days and 364 complete Year B days, not one generic daily profile

• Proposed ESS retains the lowest mean shedding in both years, with mean daily TOTEX of 51.91 and 51.56 thousand CNY/day

• A separate seven-day calculation carries SOC between days; the manuscript does not equate this test with full-year chronological co-optimization

**Changes in the revised manuscript:**

Added complete Year A/Year B evaluation and a seven-day interday-SOC comparison, and clarified what the daily cyclic-SOC model can and cannot support.

**Where this appears in the revised manuscript**

• Section 3.6, Figs 16–18 and Table 14, pp. 58–59, Lines 911–924: 366-day Year A, 364-day Year B, and seven-day interday-SOC validation.

• Discussion, Sections 4.4–4.5 (Section 4.4: p. 76, Lines 1190–1193; Section 4.5: p. 77, Lines 1204–1212): limits of daily cyclic SOC, seasonal carryover, aging mechanisms, and longer chronology.

*Excerpt from revised manuscript Table 14: “Annual and cross-year validation results for the three 33-bus planning strategies.”*

| **Year** | **Strategy** | **Days** | **Mean daily TOTEX (10³ CNY/day)** | **Worst** $\boldsymbol{V}_{\boldsymbol{min}}$ **(p.u.)** | **Maximum daily UVE (p.u.·h)** | **Mean shed energy (MWh/day)** |
| --- | --- | --- | --- | --- | --- | --- |
| Year A | No ESS | 366 | 83.59 | 0.94138 | 0.234243 | 0.113811 |
| Year A | Proposed ESS | 366 | **51.91** | **0.95395** | **0.117952** | **0.002318** |
| Year B | No ESS | 364 | 81.70 | 0.94213 | 0.222593 | 0.101495 |
| Year B | Proposed ESS | 364 | **51.56** | **0.95369** | **0.117159** | **0.000686** |

*Reproduced from the revised manuscript: Fig 18. Effect of daily SOC resetting and interday SOC coupling on Fixed ESS and Proposed ESS.*

## Reviewer 1 — Comment 10

**Reviewer comment (reproduced verbatim)**

Major Comment 10: The degradation model is oversimplified

The manuscript uses a throughput-based ESS degradation model. While computationally convenient, battery degradation depends on:

Depth of discharge.

Temperature.

Charging/discharging rate.

State-of-charge history.

The authors should discuss whether the simplified degradation model may bias ESS sizing. A sensitivity comparison with a more detailed degradation representation would improve the credibility of the results.

**Response:**

We thank the reviewer for highlighting the risk of oversimplifying battery aging. Battery aging cannot generally be represented by energy throughput alone. The current manuscript therefore uses an explicit piecewise-linear DoD-based cycle-degradation model rather than a single constant throughput charge. **Equation** (5) selects a DoD segment with binary variables and applies the corresponding marginal cycle-life cost to the daily operating pattern. This retains the effect of cycling depth while remaining compatible with the mixed-integer conic operating model.

The model is still intentionally reduced. It does not resolve cell temperature, instantaneous C-rate damage, calendar aging, chemistry-specific state-of-health dynamics, or a full electrochemical model. SOC history affects the result through the modeled trajectory and depth segments, but not through a separate long-memory aging state. We now identify these omissions directly and explain the likely direction of the uncertainty: if high temperatures, high rates, or calendar aging increase lifetime cost beyond the modeled cycle term, a design with less energy throughput or a different power-to-energy ratio could become more attractive.

The revision adds two quantitative checks that can be supported by the available data. First, the **degradation-cost multiplier is varied from 0.50 to 1.50**; the retained design’s **TOTEX ranges from 51,767.10 to 51,901.10 CNY/day** across the evaluated points. Second, **allowable DoD is varied from 60% to 80%**, producing 52,070.93, 51,937.83, and 51,826.24 CNY/day. The duration-class comparison further contrasts the best two-, three-, and four-hour alternatives. These tests show how the planning outcome responds to cycle-cost and usable-energy assumptions within the declared model.

A comparison of temperature effects, C-rate effects, and calendar aging would require cell chemistry, thermal-state, duty-cycle, and long-term state-of-health data that are not available for this benchmark. Assigning unsupported coefficients would make an apparently detailed comparison less credible. We therefore report the supported cycle-cost, DoD, and duration sensitivities, state the omitted mechanisms, and avoid claims about lifetime-optimal electrochemical sizing.

The resulting interpretation is narrower but more useful. The reported **bus 24, 1 MW/2 MWh plan** is the best candidate under the PWL DoD-based cycle model and tested parameter ranges. It may change for a different chemistry, thermal environment, rate limit, calendar-aging model, or replacement policy. We now recommend re-estimating those inputs when applying the framework to a specific storage technology.

**Changes in the revised manuscript:**

Clarified the explicit piecewise-linear DoD-based cycle-degradation model, added degradation-cost and DoD sensitivities, and stated the omitted thermal, rate, calendar, and electrochemical mechanisms.

**Where this appears in the revised manuscript**

• Section 2.2.1, Eq (5), p. 22, Lines 332–342: piecewise-linear DoD-dependent cycle-degradation model.

• Section 3.8, Fig 22 and Table 18, pp. 62–64, Lines 964–979: degradation-cost, allowable-DoD, and duration sensitivities.

• Discussion, Section 4.5, p. 77, Lines 1204–1208: omitted temperature, C-rate, calendar-aging, and chemistry-specific mechanisms.

*Excerpt from revised manuscript Table 18: “Economic and storage-model sensitivity results.”*

| **Sensitivity factor** | **Level** | **TOTEX (CNY/day)** | **Design / evaluation basis** |
| --- | --- | --- | --- |
| **Degradation-cost multiplier** | 0.50 | 51767.10 | Proposed ESS (retained design) |
| Degradation-cost multiplier | 1.00 | 51826.24 | Proposed ESS (retained design) |
| Degradation-cost multiplier | 1.50 | 51901.10 | Proposed ESS (retained design) |
| **Allowable DoD** | 60% | 52070.93 | Proposed ESS: Bus 24, 1.00 MW / 2.00 MWh |
| Allowable DoD | 70% | 51937.83 | Proposed ESS: Bus 24, 1.00 MW / 2.00 MWh |
| Allowable DoD | 80% | 51826.24 | Proposed ESS: Bus 24, 1.00 MW / 2.00 MWh |

## Reviewer 1 — Comment 11

**Reviewer comment (reproduced verbatim)**

Major Comment 11: More benchmark cases are required to validate the proposed strategy

The comparison mainly includes:

No ESS

Fixed ESS

Proposed optimized ESS

However, additional benchmarks would strengthen the paper:

ESS optimization without uncertainty.

ESS optimization without voltage constraints.

ESS optimization without TOTEX.

Robust optimization or distributionally robust ESS planning.

These comparisons would isolate which component of the proposed framework contributes most to the improvement.

**Response:**

We are grateful for this recommendation because it prompted us to broaden the evidence beyond the original three-strategy comparison. The revised benchmark package is organized around four questions: the contribution of uncertainty representation; the distinction between physical voltage outcomes and their monetary representation; the economic drivers within TOTEX; and the robustness and stability of the planning result. This structure addresses the reviewer’s underlying attribution concern while keeping each comparison scientifically well defined.

First, Table 9 compares deterministic and K10 uncertainty-aware evaluation on the same three planning strategies. **Deterministic TOTEX is 49,542.30 CNY/day for No ESS, 48,757.22 for Fixed ESS, and 46,966.82 for Proposed ESS**; **the corresponding K10 values are 73,151.02, 58,983.43, and 51,826.24 CNY/day**. The preferred strategy remains the same, while the change in absolute cost and adverse-condition exposure identifies the contribution of representing historical uncertainty. The K=3/5/10/20 comparison and CVaR sensitivity further show how scenario fidelity and risk preference affect that evidence.

Second, **Tables** 12–13 separate monetary security penalties from penalty-independent physical voltage measures, while **Tables** 7–8 examine the contribution of other flexible resources. This lets readers compare non-security operating cost, security penalties, Vmin, UVH, Dmax, UVE, MVM, and load shedding rather than infer engineering performance from one scalar objective. This evidence directly addresses whether the observed voltage benefit is merely a penalty-coefficient effect. It is not mathematically identical to an exact reoptimization without voltage constraints: removing physical security constraints changes the admissible operating region, so such a case could alter dispatch, siting, and sizing rather than simply subtract a cost term.

Third, **Table** 12 exposes the economic drivers within TOTEX. Proposed ESS has 49,482.58 CNY/day of non-security operating cost, 1,584.94 CNY/day of security penalties, and 758.72 CNY/day of annualized storage cost, for **51,826.24 CNY/day total**. An OPEX-only planning objective would make storage capacity a free resource and would therefore answer an economically different question; it is not a neutral ablation of TOTEX. The reported decomposition instead shows whether the ranking is driven by operations, security exposure, or capital recovery without removing the investment discipline that defines the planning problem.

Fourth, stability is assessed through scenario-count fidelity, K20 and full-history confirmation, complete Year A/Year B evaluation, interday-SOC validation, resource ablations, and economic, DoD, duration, voltage-threshold, degradation, and CVaR sensitivities. These are empirical robustness checks within the historical-scenario paradigm; they are not a distributionally robust optimization (DRO) model. A formal robust or DRO benchmark is a different uncertainty paradigm requiring an uncertainty or ambiguity set, a robustness budget or probability-distance choice, a dual reformulation, and a worst-case interpretation. Introducing it as if it were a switch on the current model would obscure rather than isolate the comparison.

Taken together, the added evidence substantially clarifies which modeling components support the result and where the remaining boundary lies. The study does not claim equivalence to a no-voltage-constraint reoptimization or to formal DRO, and it does not exhaust all planning paradigms. Those formulations remain valuable complementary extensions, whereas the present conclusions are supported by the empirical uncertainty, physical-security, economic-decomposition, and stability evidence actually evaluated.

**Key evidence supporting this response**

• The deterministic and K10 uncertainty-aware comparisons are reported for all three strategies on the common topology

• TOTEX components are separated from Vmin, UVH, Dmax, UVE, MVM, and shedding so that security evidence does not depend only on penalties

• Scenario, cross-year, interday, economic, storage-model, risk, and voltage-threshold checks bound the stability claim

**Changes in the revised manuscript:**

Added deterministic and K10 comparisons, resource ablations, cost/security decomposition, physical voltage measures, and risk and scenario-count sensitivities; explained why a new robust formulation was not represented as an ablation.

**Where this appears in the revised manuscript**

• Sections 3.2–3.4 and Tables 7–13 (Section 3.2: pp. 47–48, Lines 738–752; Section 3.3: pp. 54–55, Lines 848–867; Section 3.4: pp. 55–58, Lines 868–904): deterministic/K10 benchmarks, resource ablations, cost decomposition, and physical security metrics.

• Section 3.6, Figs 16–18 and Table 14 (Section 3.6: pp. 58–59, Lines 911–924; Section 3.8: pp. 62–64, Lines 964–979): sensitivity checks.

• Discussion, Sections 4.2–4.3 (Section 4.2: p. 74, Lines 1141–1148; Section 4.3: pp. 75–76, Lines 1170–1179): interpretation of uncertainty and penalty-independent security evidence.

*Relevant results from revised manuscript Table 9: “Economic comparison of the three planning strategies under deterministic and K10 uncertainty-aware evaluations.”*

| Strategy | Annualized ESS cost (CNY/day) | **Deterministic evaluation — OPEX (CNY/day)** | **Deterministic evaluation — TOTEX (CNY/day)** | **K10 uncertainty-aware evaluation — OPEX (CNY/day)** | **K10 uncertainty-aware evaluation — TOTEX (CNY/day)** |
| --- | --- | --- | --- | --- | --- |
| No ESS | 0.00 | 49542.30 | 49542.30 | 73151.02 | 73151.02 |
| Fixed ESS | 303.49 | 48453.73 | 48757.22 | 58679.94 | 58983.43 |
| Proposed ESS | 758.72 | **46208.10** | **46966.82** | **51067.52** | **51826.24** |

## Reviewer 1 — Comment 12

**Reviewer comment (reproduced verbatim)**

Major Comment 12: Reproducibility and data availability require improvement

The manuscript states that supporting information contains the underlying data. However, detailed information necessary for reproduction is still unclear.

The authors should provide:

Complete input files.

Optimization parameters.

Scenario generation code.

ESS cost parameters.

Solver settings.

Network modification details.

Since the work is computational modeling, reproducibility is essential.

**Response:**

We appreciate the reviewer’s emphasis on reproducibility. Readers of a computational planning study should have more than summary tables. The revised manuscript and shared package provide the model inputs, historical-series processing, representative-scenario information, storage planning domain, algorithm settings, solver settings, network modifications, and the source values underlying every main figure and table. The S1 Dataset caption at the end of the manuscript explicitly links the supporting data to **Tables** 1–19, S1 Table, and **Figs** 1–22.

The public version associated with this revision is archived as Zenodo v1.0.1 at https://doi.org/10.5281/zenodo.21875850. The development repository is https://github.com/mouzhenhua8/uncertainty-aware-bilevel-ess-planning. The versioned Zenodo record is the stable reference for the submitted revision, while the repository provides a convenient route for browsing the code and directory structure. Author-generated code is licensed under MIT, and author-generated data and documentation under CC BY 4.0. Third-party inputs retain their original terms.

The input information includes the modified IEEE 33-bus network, the retained topology, resource buses, candidate ESS locations, power and duration grid, tariffs, voltage criteria, economic parameters, and the released 240-bus feeder and candidate set. The historical-data preparation identifies the OPSD Denmark source, the 2016/2017 Year A/Year B split, complete-day selection, 72-dimensional daily representation, scenario counts, input-risk features, representative-set rules, membership-based weights, and cross-year use.

The optimization description now gives MATLAB R2024b, YALMIP, Gurobi 13.0.2, the target MIP gap of at most 1%, the 900 s time limit where applied, and the post-solution physical-residual tolerance. It also reports the PSO, GA, and DE populations, method parameters, common seeds, candidate limits, proposal limit, and stopping rules. Because the final 33-bus design is based on all 211 alternatives, the shared objective surface can be used to confirm both the selected candidate and the search-comparison metrics.

The figure and table source files contain the values used in the manuscript rather than only rendered images. Network and scenario notes explain the main preprocessing decisions. The Data Availability statement gives the same access information so that it remains visible after publication. These additions do not imply that every software environment will reproduce bit-identical solver timings, but they provide the scientific inputs, parameters, code, and outputs needed to reproduce and examine the reported analyses.

**Key evidence supporting this response**

• The public reproducibility record is available at GitHub and archived as Zenodo v1.0.1 (doi:10.5281/zenodo.21875850)

• Author-generated code is licensed under MIT; author-generated data and documentation are licensed under CC BY 4.0

• The S1 Dataset links processed inputs, representative scenarios, complete planning results, and source data for the reported figures and tables

**Changes in the revised manuscript:**

Released the complete code-and-data record, documented inputs and settings, added stable access and license information, and linked the S1 Dataset to all main figures and tables.

**Where this appears in the revised manuscript**

• Sections 2.1.2–2.1.3 and 2.2.2 (Section 2.1.2: pp. 14–16, Lines 213–236; Section 2.1.3: p. 16, Lines 237–243; Section 2.2.2: pp. 24–30, Lines 365–470): scenario construction and weights.

• Section 2.3 (design domain and enumeration: pp. 39–40, Lines 612–621 and 627–629; solver and benchmark settings: pp. 43–44, Lines 678–690): solver, enumeration, and PSO/GA/DE settings.

• Data Availability and Supporting information (Data Availability: pp. 84–85, Lines 1301–1312; Supporting information: p. 96): GitHub, Zenodo v1.0.1 DOI, licenses, S1 Dataset, and S1 Table.

We again thank Reviewer 1 for the careful and constructive comments, which have helped us substantially improve the clarity, rigor, and presentation of the manuscript.

Sincerely,

The Authors

# Reviewer 2

## Reviewer 2 — Comment 1

**Reviewer comment (reproduced verbatim)**

1. Please provide a more detailed explanation of the tail-preserving scenario reduction process, specifically how tail scenarios are selected and weighted. Clarifying the rationale behind the number of Monte Carlo samples (N=600) and reduced scenarios (K=5) would also be beneficial.

**Response:**

Thank you for asking us to clarify this point. The uncertainty construction has been replaced since the earlier version mentioned in the comment. The current study does not use $N = 600$ Monte Carlo draws or select $K = 5$ as its principal set. It begins with **366 complete historical Year A days**, each represented as a joint 72-dimensional load–PV–wind vector, and compares $K \in\{3, 5, 10, 20\}$.

The representative sets follow the frozen input-side protocol. The combined input-risk score uses standardized peak net load, low renewable availability, maximum absolute net-load ramp, and the fixed-topology voltage-stress proxy. For each season $s$, $H_{s}$ is the top $\left\lceil0.10 N_{s} \right\rceil$ historical days by that score, where $N_{s}$ denotes the number of complete Year A days in season $s$. $K = 3$ uses three global medoids; $K = 5$ uses one ordinary medoid per season plus the annual combined-input-risk maximum; $K = 10$ uses one ordinary and one high-risk medoid per season plus the annual high-load/low-renewable and annual high-ramp maxima; and $K = 20$ retains the $K = 10$ core plus ten constrained seasonal ordinary medoids under the frozen Hamilton allocation. Every historical day is assigned exactly once to its nearest eligible representative, and $\pi_{k}=\frac{n_{k}}{N_{A}}=\frac{n_{k}}{366}$, with no probability floor. The full-history worst-voltage date is audited only after selection: it is absent from $K = 3$ and retained as an actual representative trajectory in $K = 5$, $K = 10$, and $K = 20$.

$K = 10$ **is the principal setting** because it retains the worst-voltage day and the selected bus 24, 1 MW/2 MWh plan with a moderate computation time. Its maximum TOTEX and UVE errors are **12.49% and 42.69%**, compared with **1.40% and 11.13%** for $K = 20$; $K = 20$ requires **2771.3 s rather than 911.7 s**. We therefore present $K = 10$ **as a computational compromise** and use $K = 20$ **and full-history results as complementary checks**, not as evidence that ten scenarios are sufficient in every application.

**Key evidence supporting this response**

• For each season $s$, $H_{s}$ contains the top $\left\lceil0.10 N_{s} \right\rceil$ days by the four-feature input-risk score; membership weights are $\pi_{k}=\frac{n_{k}}{N_{A}}=\frac{n_{k}}{366}$

• $K \in\{3, 5, 10, 20\}$ sets are audited against full history for TOTEX, UVE, minimum-voltage error, exact worst-date retention, and runtime

• $K = 10$ is the principal computational compromise; $K = 20$ and full-history results are complementary higher-fidelity checks, not a universal sufficiency claim

**Changes in the revised manuscript:**

Replaced the former $N = 600$/$K = 5$ description and added the historical selection, input-risk stratification, membership weighting, exact-date-retention auditing, K sensitivity, errors, and runtime rationale.

Where this appears in the revised manuscript

• Section 2.2.2, Eqs (10)–(19), pp. 24–30, Lines 365–470: four-feature input risk, seasonal pools, K-specific selection, assignment, and membership-based weights.

• Section 3.1, Figs 6–7 and Tables 4–6, pp. 45–47, Lines 708–737: fidelity, exact worst-date retention, runtime, and design stability.

• Discussion, Section 4.2, p. 74, Lines 1141–1148: as the computational compromise and /full-history results as complementary checks.

*Reproduced from the revised manuscript: Fig 6. Historical Year A daily trajectories and the input-risk-stratified K=10 representative set for load, photovoltaic, and wind profiles.*

*Relevant results from revised manuscript Table 4: “Scenario-reduction fidelity and computational performance for K = 3, 5, 10, and 20.”*

| **K** | **Max TOTEX error (%)** | **Max UVE error (%)** | **Max \|**$\boldsymbol{V}_{\boldsymbol{min}}$ **error\| (p.u.)** | **Worst historical day retained** | **Runtime (s)** |
| --- | --- | --- | --- | --- | --- |
| 3 | 15.60 | 64.73 | 0.02052 | No | **187.8** |
| 5 | 47.65 | 149.85 | **7.12×10⁻⁵** | Yes | 448.4 |
| 10 | 12.49 | 42.69 | 6.66×10⁻⁴ | Yes | 911.7 |
| 20 | **1.40** | **11.13** | 6.35×10⁻⁴ | Yes | 2771.3 |

## Reviewer 2 — Comment 2

**Reviewer comment (reproduced verbatim)**

2.While the fixed-topology assumption is justified for attribution, consider adding a brief discussion on the potential implications or limitations of this assumption in real-world dynamic network environments, perhaps referencing future work where co-optimization with topology changes could be explored.

**Response:**

We thank the reviewer for this focused suggestion. We have expanded the discussion of when the fixed-topology assumption is practical and what it excludes. It represents situations in which the normal feeder state is prescribed by protection, maintenance, regulation, or operating policy, and it also provides a common network for attributing differences to the storage plan. The same branch states are therefore used for No ESS, Fixed ESS, and Proposed ESS after the one-time topology-identification step.

In a network with dynamic reconfiguration, switching could change loss gradients, weak-bus locations, congestion, and voltage sensitivity. The preferred storage bus, power, or duration could consequently change, and coordinated operation could produce benefits not captured here. We now state that the fixed-topology result can underestimate switching–storage synergies and that the bus 24 result is conditional on the retained 33-bus configuration.

The future extension is described as joint optimization with branch-status decisions, radiality and connectivity constraints, switching limits and costs, and protection-compatible operating rules. This would be a different counterfactual from the common-topology attribution used in this paper, so the present conclusions have been limited accordingly.

**Changes in the revised manuscript:**

Expanded the real-world interpretation and limitations of fixed topology and described the requirements for joint topology–storage planning.

**Where this appears in the revised manuscript**

• Section 2.1.1 and Fig 2, pp. 12–13, Lines 179–212 (Fig 2 caption: p. 12, Lines 189–190): retained topology and common-topology attribution protocol.

• Discussion, Section 4.4, p. 76, Lines 1180–1185: implications and possible underestimated switching–storage synergies.

• Discussion, Section 4.5, p. 77, Lines 1209–1212: joint topology–storage planning as a future extension.

*Reproduced from the revised manuscript: Fig 2. One-time topology identification for the IEEE 33-bus feeder: (a) initial feeder and switching actions; (b) fixed radial topology used in subsequent simulations.*

## Reviewer 2 — Comment 3

**Reviewer comment (reproduced verbatim)**

3. In the discussion, emphasize more strongly how the proposed framework, rather than just the numerical results, can be generalized and applied to different distribution systems and planning horizons.

**Response:**

We appreciate the reviewer’s emphasis on this distinction. We agree that the transferable element is the sequence of planning and evaluation steps rather than the numerical size selected for one feeder. The revised Discussion now separates the general framework from the case-specific inputs. For another radial distribution system, the same structure can retain a prescribed topology, form joint daily vectors from local demand and renewable records, construct season-specific empirical high-risk pools, compare representative-set sizes, evaluate a declared storage design domain through DistFlow-SOCP operating models, and report economic and physical voltage measures separately.

What must change are the feeder model, candidate buses, device capabilities, historical records, tariffs, costs, voltage criteria, and planning horizon. The released 240-bus study illustrates this distinction: it uses the same attribution logic and economy–security reporting, but a different network, candidate set, model size, and location comparison. It is evidence that the structure can be instantiated beyond the 33-bus feeder, not a claim that one set of parameters transfers unchanged.

Planning horizons can likewise be adapted. The present paper combines representative 24-hour operation, complete Year A/Year B evaluation, and a seven-day interday-SOC calculation. A seasonal or long-duration application would require longer chronological coupling, calendar-aging assumptions, and possibly a different scenario representation. We revised **Sections** 4.1 and 4.5 and the Conclusions so that the framework’s reusable steps and the case-specific conclusions are not conflated.

**Key evidence supporting this response**

• The released radial feeder has 240 buses, 239 retained branches, 16 candidate locations, and 155 finite alternatives including no-build

• The reference design improves voltage exposure, loading, overload, and loss relative to No ESS and fixed-location heuristics, while TOTEX is 65.56 CNY/day above No ESS

• This supports tested-scale instantiation and a security improvement at small incremental cost, not parameter-free transfer to arbitrary feeders

**Changes in the revised manuscript:**

Strengthened the discussion of which framework elements transfer to other feeders and horizons and which inputs must be re-estimated.

**Where this appears in the revised manuscript**

• Introduction and Table 1 (Introduction: pp. 4–5, Lines 63–75; Table 1 caption: p. 9, Lines 152–153): framework-level contribution separated from case-specific components.

• Section 3.7, Figs 19–21 and Tables 15–17, pp. 59–62, Lines 925–963: instantiation of the attribution and economy–security logic on the released 240-bus feeder.

• Discussion, Sections 4.1 and 4.5, and Conclusions (Section 4.1: pp. 73–74, Lines 1130–1140; Section 4.5: p. 77, Lines 1194–1212; Conclusions: pp. 79–80, Lines 1240–1256): transferable steps, inputs requiring re-estimation, and horizon limitations.

*Reproduced from the revised manuscript: Fig 20. Reference-versus-fixed-location comparison on the released 240-node feeder using Reference, load-center, and feeder-head locations.*

## Reviewer 2 — Comment 4

**Reviewer comment (reproduced verbatim)**

4. A thorough proofread for minor grammatical errors, typographical mistakes, and awkward phrasings is recommended. Ensuring consistent notation and breaking down overly long sentences would improve readability.

**Response:**

We thank the reviewer for encouraging a thorough language and notation review. We proofread the complete manuscript and revised sentence structure, punctuation, terminology, and notation. Long sentences in the Introduction, Methods, and Discussion were divided where this improved readability. Terms for the three strategies, the fixed topology, historical years, scenario counts, storage power and energy, TOTEX, UVE, and CVaR are now used consistently. Figure and table callouts were also checked against their captions.

We paid particular attention to equations and their surrounding definitions. Variables introduced after the Stage II DistFlow equations are defined in the text; the squared-current relation and load-shedding/power-factor expressions were corrected; equation numbering is continuous; and the notation used in the prose, tables, and captions is aligned with the equations. The revision also avoids overly broad method claims and uses shorter sentences to distinguish what was calculated from what is inferred.

**Changes in the revised manuscript:**

Proofread the manuscript throughout and standardized grammar, sentence structure, notation, numbering, and figure/table references.

**Where this appears in the revised manuscript**

• Section 2.2.3, especially Eqs (25)–(28) and (34)–(35) (Eqs (25)–(28): pp. 34–35, Lines 537–554; Eqs (34)–(35): pp. 37–38, Lines 583–591): revised variable definitions, notation, and operational equations.

• Figure/table captions and terminology throughout the revised manuscript (representative caption locations: Fig 2, p. 12, Lines 189–190; Tables 5–6, p. 46, Lines 729–733; Fig 22/Table 18, p. 62, Lines 970–972; Supporting information, p. 96): standardized notation and aligned callouts.

## Reviewer 2 — Comment 5

**Reviewer comment (reproduced verbatim)**

5.While this is practically grounded for grid-side storage, please add a brief paragraph in the Discussion (Section 4.5) analyzing how relaxing this rigid rule might affect the CAPEX-OPEX trade-off and voltage-security outcomes.

**Response:**

We thank the reviewer for drawing attention to the power–energy coupling assumption. We added the requested discussion to **Section** 4.4. The current planning domain uses rigid duration classes and a fixed power–energy relation within each class, which is practical for grid-side product definitions and keeps the finite candidate set transparent. Relaxing that rule would allow power and energy capacity to vary independently or continuously.

This added flexibility could change the CAPEX–OPEX balance in either direction. A higher power-to-energy ratio may be favored where short voltage-support events dominate, because discharge power can be increased without purchasing energy capacity that is seldom used. A lower ratio and longer duration may be favored where renewable shifting, repeated stress hours, or interday energy balance dominate. The selected bus could also change because power injection sensitivity and energy-deficit duration vary spatially.

The sensitivity study provides a discrete indication of this trade-off: the **best two-, three-, and four-hour designs have TOTEX values of 51,826.24, 51,888.42, and 51,887.63 CNY/day**, and the **discount-rate cases at 4% and 6% select the four-hour alternative**. These close values suggest that continuous sizing could refine the near-optimal ranking. It would also enlarge the search space and require clear bounds and investment curves. We now identify continuous independent power/energy sizing as an extension rather than assuming that the current duration rule is universally optimal.

**Key evidence supporting this response**

• The best two-, three-, and four-hour class minima are 51,826.24, 51,888.42, and 51,887.63 CNY/day

• The 4% and 6% discount-rate cases select the four-hour alternative, showing that the discrete duration ranking can change

• Continuous independent power/energy sizing was not solved; the reported sensitivity motivates that extension without claiming its outcome

**Changes in the revised manuscript:**

Added a Section 4.4 discussion of independent power/energy sizing and its likely effects on investment, operating cost, voltage support, and computation.

**Where this appears in the revised manuscript**

• Section 2.3, pp. 39–40, Lines 612–621 and 627–629: discrete siting–power–duration domain and fixed duration classes.

• Section 3.8, Fig 22 and Table 18, pp. 62–64, Lines 964–979: two-, three-, and four-hour class minima and financial sensitivities that alter the H2/H4 ranking.

• Discussion, Section 4.4, p. 76, Lines 1186–1189: implications and limitations of relaxing the fixed power–energy relation.

*Reproduced from the revised manuscript: Fig 22. Combined economic, ESS-model, risk, voltage-threshold, and representative-scenario sensitivity analysis.*

## Reviewer 2 — Comment 6

**Reviewer comment (reproduced verbatim)**

6. In Equations (25) and (26), there are minor notation formatting issues. Please ensure that the text following Equation (26) explicitly defines each active variable symbol cleanly. In Equation (34), the expression appears to have a typographical duplication. Please correct Equation (35) too, it contains a minor typesetting error with overlapping or misplaced symbols. Please format the power factor relationship clearly.

**Response:**

Thank you for identifying these notation problems. We corrected the Stage II DistFlow presentation in the current manuscript. The text following **Equations** (25)–(26) now introduces the branch-flow and voltage quantities before listing the active optimization variables, and the symbols associated with **Equations** (26)–(28) are defined cleanly rather than being crowded into the displayed expression.

The duplicated term in **Equation** (34) was removed, and the equation now gives the intended squared-current definition without overlap. **Equation** (35) was reset so that the load-shedding variables and the power-factor relationship are legible and mathematically separated. The surrounding text states the applicable buses and hours and explains how active and reactive curtailed demand are related. We also checked the capacitor-bank constraints and the concluding operational-constraint paragraph for consistent symbols.

These are typesetting and definition corrections; they do not change the solved formulation or numerical results. The tracked manuscript shows the revised expressions and definitions at the locations below.

**Changes in the revised manuscript:**

Corrected and clarified the variable definitions around Equations (25)–(28), the squared-current expression in Equation (34), and the load-shedding and power-factor notation in Equation (35).

**Where this appears in the revised manuscript**

• Section 2.2.3, Eqs (25)–(28), pp. 34–35, Lines 537–554: branch-flow variables and surrounding definitions.

• Section 2.2.3, Eqs (34)–(35), pp. 37–38, Lines 583–591: corrected squared-current, load-shedding, and power-factor expressions.

## Reviewer 2 — Comment 7

**Reviewer comment (reproduced verbatim)**

·7. The modified IEEE 33-bus system is radial. Please expand briefly in Section 4.5 on how the computational complexity of the lower-level DistFlow-SOCP models scales when transitioning to larger IEEE test systems.

**Response:**

We thank the reviewer for asking for a clearer account of computational scaling. We have expanded Section 4.5 and added a 240-bus case. On a radial feeder, continuous DistFlow variables and branch constraints grow approximately with buses, branches, hours, and scenarios. Actual solution time can grow faster because mixed-integer decisions, cone structure, candidate count, conditioning, and solver branching also matter. Upper-level cost is roughly the number of candidate evaluations multiplied by the cost of each declared multi-scenario evaluation.

The 240-bus formulation reports **139,000 rows, 259,848 columns, 693,648 nonzeros, and 83,136 conic or quadratic constraints**. Per-case K8 multi-scenario evaluation runtimes are **14.04-31.10 s**. Each case contains **192 scenario-hour points**; the 192-per-strategy and 768-overall counts are AC back-substitution audit points, not separate optimization runs. The **maximum SOCP-to-AC voltage mismatch is 2.13 × 10⁻⁸ p.u., and the maximum physical residual is 9.99 × 10⁻⁷**. These values give a concrete larger-system reference without extrapolating from the 33-bus timing alone.

We nevertheless avoid claiming unrestricted scalability. The 240-bus study uses a fixed radial topology and a pre-specified set of 16 candidate locations. Larger candidate domains, more scenarios, longer chronology, joint switching, or finer discrete sizing could dominate the computation. The paper now describes screening, parallel candidate evaluation, decomposition, and more selective scenario use as possible directions for larger studies, while limiting the present conclusion to executability at the tested scale.

**Key evidence supporting this response**

• The 240-bus model contains 139,000 rows, 259,848 columns, 693,648 nonzeros, and 83,136 conic/quadratic constraints

• Per-case K8 evaluation takes 14.04–31.10 s; the maximum SOCP-to-AC voltage mismatch is 2.13×10⁻⁸ p.u. and the maximum residual is 9.99×10⁻⁷

• The 192-per-strategy and 768-total values are AC-audit scenario-hour points, and the claim is limited to executability at the tested scale

**Changes in the revised manuscript:**

Expanded the scaling explanation and supported it with model size, per-case solve time, AC-audit scenario-hour count, and numerical-consistency results from the released 240-bus feeder.

**Where this appears in the revised manuscript**

• Section 3.7 and Table 17, pp. 61–62, Lines 958–963: 240-bus model size, per-case solve time, AC-audit scenario-hour count, and numerical consistency.

• Discussion, Section 4.5, p. 77, Lines 1194–1208: dependence on branches, periods, scenarios, candidates, and mixed-integer/conic structure, with claims bounded to the tested scale.

*Relevant results from revised manuscript Table 17: “Numerical consistency and computational performance of the 33-bus and released 240-bus systems.”*

| **System** | **Metric** | **Reported value** |
| --- | --- | --- |
| 33-bus | Maximum physical residual | 9.73×10⁻⁹ |
| 33-bus | Selected-design K10 evaluation runtime | 1286.78 s |
| 240-bus | Model size (rows / columns / nonzeros) | 139000 / 259848 / 693648 |
| 240-bus | Second-order-cone / quadratic constraints | 83136 |
| 240-bus | Per-case K8 evaluation runtime | 14.04–31.10 s |
| 240-bus | Maximum SOCP-to-AC voltage mismatch | 2.13×10⁻⁸ p.u. |
| 240-bus | Maximum physical residual | 9.99×10⁻⁷ |
| 240-bus | AC-audit scenario-hour points | 192 per strategy; 768 total |

We again thank the Academic Editor and Reviewers for their careful and constructive comments. We believe that these revisions have substantially improved the clarity, reproducibility, validation, and scope of the manuscript.

Sincerely,

The Authors
