# Supplementary material for: Uncertainty-aware bilevel energy storage planning for active distribution networks: A fixed-topology DistFlow-based MISOCP framework with voltage-security assessment
Source: PLoS One. 2026 Oct 9;21(10):e0353192. doi: 10.1371/journal.pone.0353192 (PMC13652237; doi:10.1371/journal.pone.0353192)
Supplement: Supplementary file 3 [file pone.0353192.s005.docx]

**Response to Reviewers**

Manuscript Number: **PONE-D-26-30578**

Title: **Uncertainty-aware bilevel energy storage planning for active distribution networks: A fixed-topology DistFlow-based MISOCP framework with voltage-security assessment**

We thank the Academic Editor and Reviewer 2 for the careful and constructive assessment. We revised the manuscript to address the four comments through a focused ESS-aware topology robustness check, more precise terminology, targeted language and notation corrections, and clearer limits on the scope of the conclusions. All reported experimental values were preserved, and the revisions are identified in the tracked manuscript.

**Response to Reviewer 2**

**Comment 1**

**Reviewer comment**

Reframe the contribution and conclusions as conditional on a baseline fixed topology, or add an ESS-aware topology comparison.

**Response 1:** We thank the reviewer for identifying this evidence boundary. The common baseline radial topology is retained for the No ESS, Fixed ESS, and Proposed ESS comparison because it provides controlled attribution: differences among those strategies arise from the storage plans rather than from a moving network configuration. We agree that the contribution and conclusions should be conditional on that protocol.

We therefore added a focused ESS-aware topology robustness check. The retained Proposed ESS was fixed at bus 24 (1.00 MW / 2.00 MWh, 2 h), and the same Stage-I topology-identification procedure used for the baseline reference network was repeated with the ESS active. The baseline open-branch set is **[18, 22, 31, 33, 34]**, whereas the ESS-aware set is **[18, 22, 26, 33, 34]**. Thus, branch 26 changed from closed to open and branch 31 from open to closed, giving a Hamming distance of 2.

The ESS-aware topology was then fixed and evaluated using the same K10 operational model used in the main comparison. Relative to the selected Proposed ESS on the baseline topology, TOTEX increased from **51,826.24** to **53,762.99** CNY/day (**+3.74%**). Vmin increased from **0.954051** to **0.955298** p.u., UVH decreased from **0.2486** to **0.1913** h, and UVE decreased from **0.001740** to **0.001516** p.u.·h. Load shedding increased from **0.004297** to **0.006224** MWh, and network loss increased from **1.3973** to **1.7063** MWh.

We interpret this result as a mixed economy-security trade-off rather than uniform improvement. The revised Abstract, Section 3.9, and Discussion state that the check holds the ESS design fixed and does not constitute joint topology-storage planning or establish a global topology optimum.

**Amendments in the Manuscript**

Added Section 3.9 and Table 20 to report an ESS-aware topology robustness check; added a concise robustness result to the Abstract; clarified the scope and limitation of topology-related conclusions in Discussion Section 4.4.

**Location in the revised manuscript**

Abstract (introduction of the ESS-aware topology check); Section 3.9 and Table 20; Discussion Section 4.4.

**Comment 2**

**Reviewer comment**

Revise terminology and narrow claims about bilevel optimization, validation, confirmation, scalability, and generalizability.

**Response 2:** We agree and have narrowed these terms. Section 2.3 now defines bilevel as the nested planning-operation structure in which the upper layer selects one discrete ESS plan and the lower layer evaluates its optimized operational response. The reported 33-bus solution is obtained by exhaustive enumeration over the declared finite domain; the manuscript does not present a new general-purpose bilevel algorithm.

We revised uses of ‘validation’ and ‘confirmation’ where those terms could overstate the evidence provided by the corresponding analyses. The cross-year and seven-day analyses are described as robustness or chronological checks, K20 as a higher-fidelity scenario-set check, the released 240-bus analysis as an engineering-scale case study, and Fig 21 as a severe-stress test. The Section 2.3 statement that the lower-level model is solved to optimality was replaced with wording tied to the stated numerical solution criteria, and optimal OPEX was replaced with resulting OPEX.

The revised Discussion distinguishes tested-scale evidence for the reported 33-bus and 240-bus cases from proof of scalability to thousand-bus systems or general performance across arbitrary feeder types. The Abstract now qualifies the findings by the tested systems and assumptions, while the Conclusions retain their case-specific boundary.

**Amendments in the Manuscript**

Added the manuscript-specific bilevel definition in Section 2.3; revised the lower-level value statement; renamed Sections 3.6 and 3.7; revised related captions and claims; qualified the Abstract and retained the case-specific conclusion.

**Location in the revised manuscript**

Abstract; Sections 1, 2.1–2.3; Section 3 (opening paragraph); Sections 3.1, 3.6–3.7; Tables 1, 2, 5, and 14; Fig 21; Discussion Sections 4.1 and 4.5; Section 5 (Conclusions).

**Comment 3**

**Reviewer comment**

Perform a careful language, notation, equation, and unit edit.

**Response 3:** We completed a targeted language, notation, equation-reference, and unit edit without changing the experimental values. The single Abbreviations inconsistency UVDmax was corrected to Dmax, consistent with Equation (44), the main text, and the result tables. The inconsistent ESS-rating notation in Table 2 was removed to avoid introducing an undefined relationship and to maintain consistency with the defined ESS power-duration relationship.

We corrected ESS-rating presentation to 1.00 MW / 2.00 MWh where needed, daily expenditure to CNY/day, checked cumulative undervoltage exposure in p.u.·h, the 14.04–31.10 s numerical range, and the isolated Eqs. reference to Equations. Scientific notation, equation references, ESS ratings, and units were checked for consistency, while all other Word equation objects and reported numerical values were preserved.

**Amendments in the Manuscript**

Corrected $D_{max}$ in Abbreviations, removed the inconsistent ESS-rating notation in Table 2, repaired the duplicated contribution wording, standardized the specified units and equation references, and verified scientific-notation and equation-formatting consistency.

**Location in the revised manuscript**

Abstract; Section 1; Table 2; Section 3.4.1; Section 3.4.2 (Table 12 note); Discussion Section 4.5; Abbreviations (Dmax, consistent with Section 2.3, Equation (44)); Supporting information (S1 Dataset description).

**Comment 4**

**Reviewer comment**

The work could become a useful and reproducible case study if the authors more rigorously separate what is demonstrated by the fixed-topology, finite-scenario experiment from what is claimed about general ESS planning.

**Response 4:** We agree and have made this separation explicit. What is demonstrated is case-specific: within the declared 211-alternative 33-bus domain, the K10 historical scenario set, the baseline radial topology, and the stated cost model, the selected bus-24 design provides the reported economy-security result. The cross-year, seven-day, K20, released 240-bus, and ESS-aware-topology analyses are presented as complementary checks with clearly defined roles rather than universal proof.

The manuscript also states the limits of what is demonstrated. The manuscript does not establish a universal ESS size or location, savings for all feeders, field performance, scalability to arbitrary network sizes, general performance across feeder types, a globally optimal post-ESS topology, or joint topology-storage optimality. The broader contribution is therefore framed as a reproducible planning and attribution framework supported by numerical evidence in the tested cases.

**Amendments in the Manuscript**

Qualified the Abstract and retained the case-specific boundary in the Conclusions; retained the finite-domain description in Section 2.3; clarified tested-scale applicability in Section 4.5; and bounded the new topology sensitivity result in Sections 3.9 and 4.4.

**Location in the revised manuscript**

Abstract (K10 and baseline fixed-topology scope); Section 2.3; Section 3.9 and Table 20; Discussion Sections 4.1, 4.4–4.5; Section 5 (Conclusions).

We again thank the Academic Editor and Reviewers for their careful and constructive comments. We believe that these revisions have substantially improved the clarity, reproducibility, validation, and scope of the manuscript.

Sincerely,

The Authors
