# Supplementary material for: Uncertainty-aware bilevel energy storage planning for active distribution networks: A fixed-topology DistFlow-based MISOCP framework with voltage-security assessment
Source: PLoS One. 2026 Oct 9;21(10):e0353192. doi: 10.1371/journal.pone.0353192 (PMC13652237; doi:10.1371/journal.pone.0353192)
Supplement: Peer review history

# Peer review: Decision Letter 0

**DOI:** 10.1371/journal.pone.0353192.r001

**Authors:** Songpo Yang

17 Jul 2026

Dear Dr. wang,

Thank you for submitting your manuscript to PLOS ONE. After careful consideration, we feel that it has merit but does not fully meet PLOS ONE’s publication criteria as it currently stands. Therefore, we invite you to submit a revised version of the manuscript that addresses the points raised during the review process.

Please submit your revised manuscript Sep 15 2026 11:59PM. If you will need more time than this to complete your revisions, please reply to this message or contact the journal office at plosone@plos.org. When you're ready to submit your revision, log on to https://www.editorialmanager.com/pone/ and select the 'Submissions Needing Revision' folder to locate your manuscript file.

- A letter that responds to each point raised by the academic editor and reviewer(s). You should upload this letter as a separate file labeled 'Response to Reviewers'.
- A marked-up copy of your manuscript that highlights changes made to the original version. You should upload this as a separate file labeled 'Revised Manuscript with Track Changes'.
- An unmarked version of your revised paper without tracked changes. You should upload this as a separate file labeled 'Manuscript'.

If you would like to make changes to your financial disclosure, please include your updated statement in your cover letter. Guidelines for resubmitting your figure files are available below the reviewer comments at the end of this letter.

If applicable, we recommend that you deposit your laboratory protocols in protocols.io to enhance the reproducibility of your results. Protocols.io assigns your protocol its own identifier (DOI) so that it can be cited independently in the future. For instructions see: https://journals.plos.org/plosone/s/submission-guidelines#loc-laboratory-protocols. Additionally, PLOS ONE offers an option for publishing peer-reviewed Lab Protocol articles, which describe protocols hosted on protocols.io. Read more information on sharing protocols at https://plos.org/protocols?utm_medium=editorial-email&utm_source=authorletters&utm_campaign=protocols.

As the corresponding author, your ORCID iD is verified in the submission system and will appear in the published article. PLOS supports the use of ORCID, and we encourage all coauthors to register for an ORCID iD and use it as well. Please encourage your coauthors to verify their ORCID iD within the submission system before final acceptance, as unverified ORCID iDs will not appear in the published article. *Only* the individual author can complete the verification step; PLOS staff *cannot* verify ORCID iDs on behalf of authors.

We look forward to receiving your revised manuscript.

Kind regards,

Songpo Yang

Academic Editor

PLOS One

Journal Requirements:

When submitting your revision, we need you to address these additional requirements.

1. Please ensure that your manuscript meets PLOS ONE's style requirements, including those for file naming. The PLOS ONE style templates can be found at

https://journals.plos.org/plosone/s/file?id=wjVg/PLOSOne_formatting_sample_main_body.pdfand https://journals.plos.org/plosone/s/file?id=ba62/PLOSOne_formatting_sample_title_authors_affiliations.pdf

2. Please note that PLOS One has specific guidelines on code sharing for submissions in which author-generated code underpins the findings in the manuscript. In these cases, we expect all author-generated code to be made available without restrictions upon publication of the work. Please review our guidelines at https://journals.plos.org/plosone/s/materials-and-software-sharing#loc-sharing-code and ensure that your code is shared in a way that follows best practice and facilitates reproducibility and reuse.

3. Please provide a complete Data Availability Statement in the submission form, ensuring you include all necessary access information or a reason for why you are unable to make your data freely accessible. If your research concerns only data provided within your submission, please write "All data are in the manuscript and/or supporting information files" as your Data Availability Statement.

4. Please include captions for your Supporting Information files at the end of your manuscript, and update any in-text citations to match accordingly. Please see our Supporting Information guidelines for more information: http://journals.plos.org/plosone/s/supporting-information.

5. If the reviewer comments include a recommendation to cite specific previously published works, please review and evaluate these publications to determine whether they are relevant and should be cited. There is no requirement to cite these works unless the editor has indicated otherwise.

[Note: HTML markup is below. Please do not edit.]

Reviewers' comments:

Reviewer's Responses to Questions

**Comments to the Author**

1. Is the manuscript technically sound, and do the data support the conclusions?

Reviewer #1: Partly

Reviewer #2: Yes

**********

2. Has the statistical analysis been performed appropriately and rigorously?

Reviewer #1: No

Reviewer #2: Yes

**********

3. Have the authors made all data underlying the findings in their manuscript fully available?

The PLOS Data policy

Reviewer #1: No

Reviewer #2: Yes

**********

4. Is the manuscript presented in an intelligible fashion and written in standard English?

Reviewer #1: Yes

Reviewer #2: Yes

**********

Reviewer #1: Review Due Information

Event: Review Due

Journal/Organizer: PLOS ONE

Review Title: Uncertainty-aware bilevel energy storage planning for active distribution networks: A fixed-topology DistFlow-SOCP framework with voltage-security assessment

Due Date: 17 July 2026

End Date: 18 July 2026

Time Zone: US-Eastern

I reviewed the manuscript as a PLOS ONE reviewer. The paper has a relevant topic and a reasonable framework, but several methodological and validation issues need clarification before it can be accepted. I would recommend Major Revision. Below are 12 focused major comments (not superficial comments) that can be directly used in the reviewer report.

The manuscript proposes an uncertainty-aware bilevel ESS planning framework combining scenario reduction, fixed-topology DistFlow-SOCP operation, TOTEX evaluation, and voltage-security indicators on a modified IEEE 33-bus system.

Comment 1: Novelty compared with existing bilevel ESS planning studies needs stronger justification

The manuscript claims an integrated framework combining uncertainty modeling, fixed-topology attribution, DistFlow-SOCP, TOTEX, and voltage-security assessment. However, many of these components have already been investigated individually in previous studies. The authors should more clearly explain what fundamental methodological advancement is introduced beyond combining existing techniques.

In particular, the novelty should be distinguished from previous stochastic ESS planning, robust optimization, and bilevel distribution network expansion studies. A quantitative comparison table including recent ESS planning methods (objective function, uncertainty treatment, network model, optimization approach, and limitations) would strengthen the contribution.

Major Comment 2: The assumption of fixed topology limits the practical applicability of the proposed framework

The manuscript emphasizes fixed topology to avoid changing baselines and isolate ESS benefits. While this is useful for attribution, active distribution networks increasingly rely on joint optimization of ESS placement, network reconfiguration, and flexible resources.

The authors should discuss:

Under what practical conditions is topology assumed fixed?

How would the optimal ESS location change if reconfiguration is allowed?

Could the proposed approach underestimate the benefit of coordinated ESS and switching actions?

The limitation section acknowledges this issue, but further sensitivity analysis is required.

Major Comment 3: The uncertainty generation methodology requires more detailed validation

The proposed method relies on Monte Carlo sampling and scenario reduction (600 samples reduced to 5 scenarios). However, the manuscript does not sufficiently demonstrate whether five scenarios are enough to represent uncertainty.

The authors should provide:

Comparison between original Monte Carlo results and reduced scenarios.

Error evaluation after scenario reduction.

Sensitivity analysis using different numbers of scenarios (e.g., K = 3, 5, 10, 20).

Explanation of why the selected tail scenario probability floor is appropriate.

Currently, the conclusion that tail risks are preserved is not sufficiently demonstrated.

Major Comment 4: The renewable and load uncertainty models appear highly synthetic

The uncertainty model uses generated PV, wind, and load trajectories. However, realistic distribution planning requires historical or forecast-based uncertainty characteristics.

The authors should clarify:

Are the uncertainty distributions derived from real operational data?

How are seasonal variations considered?

How are extreme renewable events represented?

Since the main contribution depends strongly on uncertainty effects, unrealistic uncertainty assumptions may significantly influence the ESS value estimation.

Major Comment 5: The economic assumptions for ESS investment need stronger justification

The ESS planning result strongly depends on CAPEX, degradation cost, discount rate, and lifetime assumptions. The manuscript uses:

Power cost = 1500 CNY/kW

Energy cost = 1800 CNY/kWh

Discount rate = 8%

Lifetime = 10 years

However, these assumptions are not sufficiently justified.

The authors should provide:

Sources of ESS cost parameters.

Sensitivity analysis for future battery cost reduction.

Impact of different lifetime assumptions.

Influence of degradation cost coefficients.

Without this analysis, the economic conclusions may not be transferable.

Major Comment 6: The voltage-security improvement requires more rigorous interpretation

The manuscript reports improvements in undervoltage hours, depth, and accumulated undervoltage energy. For example, stochastic ESS reduces UVH from 17 h to 14 h and improves voltage-related indicators.

However, the actual voltage violations appear relatively small (for example, minimum voltage deviations are close to the threshold). The authors should discuss:

Whether these improvements are practically significant.

Whether the voltage-security benefit justifies ESS investment.

Comparison with alternative voltage control methods such as OLTC, inverter reactive power control, or capacitor switching.

Major Comment 7: The optimization algorithm comparison is insufficient

The manuscript compares PSO, DE, and GA. However, these algorithms are heuristic methods and their performance depends strongly on parameter settings.

The authors should provide:

Population size.

Iteration number.

Parameter tuning procedure.

Stopping criteria.

Number of independent runs.

Additionally, comparison with deterministic optimization methods or mathematical programming approaches would strengthen confidence in the obtained ESS sizing solution.

Major Comment 8: The scalability of the proposed bilevel framework is unclear

The study is demonstrated only on a modified IEEE 33-bus feeder. Modern distribution networks may contain hundreds or thousands of buses.

The authors should provide computational complexity analysis:

How does computation time increase with network size?

How many upper-level optimization evaluations are required?

Can the method handle multi-day or seasonal horizons?

The current validation does not demonstrate practical scalability.

Major Comment 9: The 24-hour scheduling horizon may underestimate long-term ESS behavior

The lower-level operation considers only a daily 24-hour horizon. However, ESS planning decisions involve:

Seasonal renewable variation.

Long-duration energy shifting.

Battery degradation over years.

Annual economic performance.

The authors should justify why a single-day horizon is sufficient for investment planning or provide additional multi-day/seasonal validation.

Major Comment 10: The degradation model is oversimplified

The manuscript uses a throughput-based ESS degradation model. While computationally convenient, battery degradation depends on:

Depth of discharge.

Temperature.

Charging/discharging rate.

State-of-charge history.

The authors should discuss whether the simplified degradation model may bias ESS sizing. A sensitivity comparison with a more detailed degradation representation would improve the credibility of the results.

________________________________________

Major Comment 11: More benchmark cases are required to validate the proposed strategy

The comparison mainly includes:

No ESS

Fixed ESS

Proposed optimized ESS

However, additional benchmarks would strengthen the paper:

ESS optimization without uncertainty.

ESS optimization without voltage constraints.

ESS optimization without TOTEX.

Robust optimization or distributionally robust ESS planning.

These comparisons would isolate which component of the proposed framework contributes most to the improvement.

Major Comment 12: Reproducibility and data availability require improvement

The manuscript states that supporting information contains the underlying data. However, detailed information necessary for reproduction is still unclear.

The authors should provide:

Complete input files.

Optimization parameters.

Scenario generation code.

ESS cost parameters.

Solver settings.

Network modification details.

Since the work is computational modeling, reproducibility is essential.

Overall Reviewer Recommendation

Recommendation: Major Revision

The manuscript addresses an important problem in active distribution network planning and presents a potentially useful framework. The integration of uncertainty-aware ESS planning with voltage-security metrics is interesting. However, the current version requires stronger validation, clearer novelty positioning, improved uncertainty justification, and more comprehensive sensitivity/scalability analysis before the conclusions can be considered sufficiently supported.

Reviewer #2: This manuscript presents a valuable contribution to the field of energy storage planning in active distribution networks, particularly through its uncertainty-aware bilevel framework and robust voltage-security assessment. The suggested revisions are primarily aimed at enhancing clarity, providing further detail on certain methodological aspects, and refining the language:

1. Please provide a more detailed explanation of the tail-preserving scenario reduction process, specifically how tail scenarios are selected and weighted. Clarifying the rationale behind the number of Monte Carlo samples (N=600) and reduced scenarios (K=5) would also be beneficial.

2.While the fixed-topology assumption is justified for attribution, consider adding a brief discussion on the potential implications or limitations of this assumption in real-world dynamic network environments, perhaps referencing future work where co-optimization with topology changes could be explored.

3. In the discussion, emphasize more strongly how the proposed framework, rather than just the numerical results, can be generalized and applied to different distribution systems and planning horizons.

4. A thorough proofread for minor grammatical errors, typographical mistakes, and awkward phrasings is recommended. Ensuring consistent notation and breaking down overly long sentences would improve readability.

5.While this is practically grounded for grid-side storage, please add a brief paragraph in the Discussion (Section 4.5) analyzing how relaxing this rigid rule might affect the CAPEX-OPEX trade-off and voltage-security outcomes.

6. In Equations (25) and (26), there are minor notation formatting issues. Please ensure that the text following Equation (26) explicitly defines each active variable symbol cleanly. In Equation (34), the expression appears to have a typographical duplication. Please correct Equation (35) too, it contains a minor typesetting error with overlapping or misplaced symbols. Please format the power factor relationship clearly.

7. The modified IEEE 33-bus system is radial. Please expand briefly in Section 4.5 on how the computational complexity of the lower-level DistFlow-SOCP models scales when transitioning to larger IEEE test systems.

**********

what does this mean?). If published, this will include your full peer review and any attached files.

If you choose “no”, your identity will remain anonymous but your review may still be made public.

**Do you want your identity to be public for this peer review?** For information about this choice, including consent withdrawal, please see our Privacy Policy

Reviewer #1: No

Reviewer #2: No

**********

[NOTE: If reviewer comments were submitted as an attachment file, they will be attached to this email and accessible via the submission site. Please log into your account, locate the manuscript record, and check for the action link "View Attachments". If this link does not appear, there are no attachment files.]

To ensure your figures meet our technical requirements, please review our figure guidelines: https://journals.plos.org/plosone/s/figures

You may also use PLOS’s free figure tool, NAAS, to help you prepare publication quality figures: https://journals.plos.org/plosone/s/figures#loc-tools-for-figure-preparation.

NAAS will assess whether your figures meet our technical requirements by comparing each figure against our figure specifications.

# Author comment: Author response to Decision Letter 1

**DOI:** 10.1371/journal.pone.0353192.r002

19 Aug 2026

Thank you to the Academic Editor and the reviewers for their careful and constructive comments. We have revised the manuscript extensively in response to all comments and have provided a detailed, point-by-point response in the separately uploaded “Response to Reviewers” file. The response document reproduces each reviewer comment, provides our corresponding response, summarizes the changes made to the revised manuscript, and gives specific page and line references to the tracked manuscript. Relevant figures, tables, and quantitative evidence are also included in the response document where appropriate to facilitate evaluation of the revision. All reviewer and editorial comments have been addressed individually.

Attachment Submitted filename: Response.docx

# Peer review: Decision Letter 1

**DOI:** 10.1371/journal.pone.0353192.r003

**Authors:** Songpo Yang

11 Sep 2026

Uncertainty-aware bilevel energy storage planning for active distribution networks: A fixed-topology DistFlow-based MISOCP framework with voltage-security assessment

PLOS One

Dear Dr.  wang,

Thank you for submitting your manuscript to PLOS One. After careful consideration, we feel that it has merit but does not fully meet PLOS One’s publication criteria as it currently stands. Therefore, we invite you to submit a revised version of the manuscript that addresses the points raised during the review process.

Please submit your revised manuscript by Oct 11 2026 11:59PM. If you will need more time than this to complete your revisions, please reply to this message or contact the journal office at plosone@plos.org. When you're ready to submit your revision, log on to https://www.editorialmanager.com/pone/ and select the 'Submissions Needing Revision' folder to locate your manuscript file.

Please include the following items when submitting your revised manuscript:

- A letter that responds to each point raised by the academic editor and reviewer(s). You should upload this letter as a separate file labeled 'Response to Reviewers'.
- A marked-up copy of your manuscript that highlights changes made to the original version. You should upload this as a separate file labeled 'Revised Manuscript with Track Changes'.
- An unmarked version of your revised paper without tracked changes. You should upload this as a separate file labeled 'Manuscript'.

If you would like to make changes to your financial disclosure, please include your updated statement in your cover letter. Guidelines for resubmitting your figure files are available below the reviewer comments at the end of this letter.

If applicable, we recommend that you deposit your laboratory protocols in protocols.io to enhance the reproducibility of your results. Protocols.io assigns your protocol its own identifier (DOI) so that it can be cited independently in the future. For instructions see: https://journals.plos.org/plosone/s/submission-guidelines#loc-laboratory-protocols. Additionally, PLOS One offers an option for publishing peer-reviewed Lab Protocol articles, which describe protocols hosted on protocols.io. Read more information on sharing protocols at https://plos.org/protocols?utm_medium=editorial-email&utm_source=authorletters&utm_campaign=protocols.

As the corresponding author, your ORCID iD is verified in the submission system and will appear in the published article. PLOS supports the use of ORCID, and we encourage all coauthors to register for an ORCID iD and use it as well. Please encourage your coauthors to verify their ORCID iD within the submission system before final acceptance, as unverified ORCID iDs will not appear in the published article. *Only* the individual author can complete the verification step; PLOS staff *cannot* verify ORCID iDs on behalf of authors.

We look forward to receiving your revised manuscript.

Kind regards,

Songpo Yang

Academic Editor

PLOS One

Journal Requirements:

1. If the reviewer comments include a recommendation to cite specific previously published works, please review and evaluate these publications to determine whether they are relevant and should be cited. There is no requirement to cite these works unless the editor has indicated otherwise.

2. Please review your reference list to ensure that it is complete and correct. If you have cited papers that have been retracted, please include the rationale for doing so in the manuscript text, or remove these references and replace them with relevant current references. Any changes to the reference list should be mentioned in the rebuttal letter that accompanies your revised manuscript. If you need to cite a retracted article, indicate the article’s retracted status in the References list and also include a citation and full reference for the retraction notice.

[Note: HTML markup is below. Please do not edit.]

Reviewer's Responses to Questions

**Comments to the Author**

Reviewer #1: All comments have been addressed

Reviewer #2: All comments have been addressed

**********

2. Is the manuscript technically sound, and do the data support the conclusions?

Reviewer #1: Yes

Reviewer #2: Yes

**********

3. Has the statistical analysis been performed appropriately and rigorously?

Reviewer #1: (No Response)

Reviewer #2: Yes

**********

4. Have the authors made all data underlying the findings in their manuscript fully available?

The PLOS Data policy

Reviewer #1: (No Response)

Reviewer #2: Yes

**********

5. Is the manuscript presented in an intelligible fashion and written in standard English?

Reviewer #1: (No Response)

Reviewer #2: Yes

**********

Reviewer #1: (No Response)

Reviewer #2: Before the manuscript can be considered further, I recommend that the authors:

1. Reframe the contribution and conclusions as conditional on a baseline fixed topology, or add an ESS-aware topology comparison.

2. Revise terminology and narrow claims about bilevel optimization, validation, confirmation, scalability, and generalizability.

3. Perform a careful language, notation, equation, and unit edit.

4. The work could become a useful and reproducible case study if the authors more rigorously separate what is demonstrated by the fixed-topology, finite-scenario experiment from what is claimed about general ESS planning.

**********

what does this mean?). If published, this will include your full peer review and any attached files.

If you choose “no”, your identity will remain anonymous but your review may still be made public.

**Do you want your identity to be public for this peer review?** For information about this choice, including consent withdrawal, please see our Privacy Policy

Reviewer #1: No

Reviewer #2: No

**********

[NOTE: If reviewer comments were submitted as an attachment file, they will be attached to this email and accessible via the submission site. Please log into your account, locate the manuscript record, and check for the action link "View Attachments". If this link does not appear, there are no attachment files.]

To ensure your figures meet our technical requirements, please review our figure guidelines: https://journals.plos.org/plosone/s/figures

You may also use PLOS’s free figure tool, NAAS, to help you prepare publication quality figures: https://journals.plos.org/plosone/s/figures#loc-tools-for-figure-preparation.

NAAS will assess whether your figures meet our technical requirements by comparing each figure against our figure specifications.

# Author comment: Author response to Decision Letter 2

**DOI:** 10.1371/journal.pone.0353192.r004

14 Sep 2026

Dear Editor and Reviewers,

Thank you for the valuable comments and suggestions. We have carefully revised the manuscript and addressed all reviewer comments.

A detailed point-by-point response to each comment has been provided in the uploaded Response to Reviewers file.

We hope that the revised manuscript is now suitable for publication in PLOS ONE.

Sincerely,

The authors

Attachment Submitted filename: response_auresp_2.docx

# Peer review: Decision Letter 2

**DOI:** 10.1371/journal.pone.0353192.r005

**Authors:** Songpo Yang

23 Sep 2026

Uncertainty-aware bilevel energy storage planning for active distribution networks: A fixed-topology DistFlow-based MISOCP framework with voltage-security assessment

PONE-D-26-30578R2

Dear Dr. Wang,

We’re pleased to inform you that your manuscript has been judged scientifically suitable for publication and will be formally accepted for publication once it meets all outstanding technical requirements.

Within one week, you’ll receive an e-mail detailing the required amendments. When these have been addressed, you’ll receive a formal acceptance letter and your manuscript will be scheduled for publication.

An invoice will be generated when your article is formally accepted. Please note, if your institution has a publishing partnership with PLOS and your article meets the relevant criteria, all or part of your publication costs will be covered. Please make sure your user information is up-to-date by logging into Editorial Manager at Editorial Manager® and clicking the ‘Update My Information' link at the top of the page. For questions related to billing, please contact billing support.

If your institution or institutions have a press office, please notify them about your upcoming paper to help maximize its impact. If they’ll be preparing press materials, please inform our press team as soon as possible -- no later than 48 hours after receiving the formal acceptance. Your manuscript will remain under strict press embargo until 2 pm Eastern Time on the date of publication. For more information, please contact onepress@plos.org.

Kind regards,

Songpo Yang

Academic Editor

PLOS One

Additional Editor Comments (optional):

Reviewers' comments:

Reviewer's Responses to Questions

**Comments to the Author**

Reviewer #2: All comments have been addressed

**********

2. Is the manuscript technically sound, and do the data support the conclusions?

Reviewer #2: (No Response)

**********

3. Has the statistical analysis been performed appropriately and rigorously?

Reviewer #2: (No Response)

**********

4. Have the authors made all data underlying the findings in their manuscript fully available?

The PLOS Data policy

Reviewer #2: (No Response)

**********

5. Is the manuscript presented in an intelligible fashion and written in standard English?

Reviewer #2: (No Response)

**********

Reviewer #2: (No Response)

**********

what does this mean?). If published, this will include your full peer review and any attached files.

If you choose “no”, your identity will remain anonymous but your review may still be made public.

**Do you want your identity to be public for this peer review?** For information about this choice, including consent withdrawal, please see our Privacy Policy

Reviewer #2: No

**********

# Editor report: Acceptance letter

**DOI:** 10.1371/journal.pone.0353192.r006

**Authors:** Songpo Yang

PONE-D-26-30578R2

PLOS One

Dear Dr. wang,

I'm pleased to inform you that your manuscript has been deemed suitable for publication in PLOS One. Congratulations! Your manuscript is now being handed over to our production team.

At this stage, our production department will prepare your paper for publication. This includes ensuring the following:

* All references, tables, and figures are properly cited

* All relevant supporting information is included in the manuscript submission,

* There are no issues that prevent the paper from being properly typeset

You will receive further instructions from the production team, including instructions on how to review your proof when it is ready. Please keep in mind that we are working through a large volume of accepted articles, so please give us a few days to review your paper and let you know the next and final steps.

Lastly, if your institution or institutions have a press office, please let them know about your upcoming paper now to help maximize its impact. If they'll be preparing press materials, please inform our press team within the next 48 hours. Your manuscript will remain under strict press embargo until 2 pm Eastern Time on the date of publication. For more information, please contact onepress@plos.org.

You will receive an invoice from PLOS for your publication fee after your manuscript has reached the completed accept phase. If you receive an email requesting payment before acceptance or for any other service, this may be a phishing scheme. Learn how to identify phishing emails and protect your accounts at https://explore.plos.org/phishing.

If we can help with anything else, please email us at customercare@plos.org.

Thank you for submitting your work to PLOS One and supporting open access.

Kind regards,

PLOS One Editorial Office Staff

on behalf of

Dr. Songpo Yang

Academic Editor

PLOS One
